# Learning a generalized graph transformer for protein function prediction in dissimilar sequences

**DOI:** 10.1093/gigascience/giae093

**Published:** 2024-12-05

**Authors:** Yiwei Fu, Zhonghui Gu, Xiao Luo, Qirui Guo, Luhua Lai, Minghua Deng

**Affiliations:** School of Mathematical Sciences, Peking University, Beijing 100871, China; Peking-Tsinghua Center for Life Sciences, Peking University, Beijing 100871, China; Department of Computer Science, University of California, Los Angeles, CA 90024, USA; Center for Quantitative Biology, Peking University, Beijing 100871, China; Peking-Tsinghua Center for Life Sciences, Peking University, Beijing 100871, China; Center for Quantitative Biology, Peking University, Beijing 100871, China; School of Mathematical Sciences, Peking University, Beijing 100871, China; Center for Quantitative Biology, Peking University, Beijing 100871, China; Center for Statistical Science, Peking University, Beijing 100871, China

**Keywords:** protein function prediction, low sequence identity, domain adaptation, adversarial learning, graph transformer

## Abstract

**Background:**

In the face of a growing disparity between high-throughput sequence data and low-throughput experimental studies, the emerging field of deep learning stands as a promising alternative. Generally, many data-driven approaches are capable of facilitating fast and accurate predictions of protein functions. Nevertheless, the inherent statistical nature of deep learning techniques may limit their generalization capabilities when applied to novel nonhomologous proteins that diverge significantly from existing ones.

**Results:**

In this work, we herein propose a novel, generalized approach named Graph Adversarial Learning with Alignment (GALA) for protein function prediction. Our GALA method integrates a graph transformer architecture with an attention pooling module to extract embeddings from both protein sequences and structures, facilitating unified learning of protein representations. Particularly noteworthy, GALA incorporates a domain discriminator conditioned on both learnable representations and predicted probabilities, which undergoes adversarial learning to ensure representation invariance across diverse environments. To optimize the model with abundant label information, we generate label embeddings in the hidden space, explicitly aligning them with protein representations. Benchmarked on datasets derived from the PDB database and Swiss-Prot database, our GALA achieves considerable performance comparable to several state-of-the-art methods. Even more, GALA demonstrates wonderful biological interpretability by identifying significant functional residues associated with Gene Ontology terms through class activation mapping.

**Conclusions:**

GALA, which leverages adversarial learning and label embedding alignment to acquire domain-invariant protein representations, exhibits outstanding generalizability in function prediction for proteins from previously unseen sequence space. By incorporating the structures predicted by AlphaFold2, GALA demonstrates significant potential for function annotation in newly discovered sequences. A detailed implementation of our GALA is available at https://github.com/fuyw-aisw/GALA.

Key Points We propose GALA, an innovative and generalized approach for protein function prediction, leveraging adversarial learning and label embedding alignment to ensure representation invariance across diverse environments and dissimilar protein sequences.

Comprehensive experimental evaluations efficiently demonstrate that GALA outperforms several state-of-the-art methods, showcasing exceptional generalizability and interpretability. This positions GALA as well suited for protein function prediction in dissimilar sequences.

## Introduction

Proteins serve as the primary catalysts, structural components, signaling messengers, and molecular machines in biological tissues, playing a significant role in diverse biological processes [1]. Additionally, protein function prediction is a crucial challenge in comprehending the roles of proteins within biological systems, which holds of great significance implications for disease research, pharmaceutical discovery, and various domains of biotechnology and bioinformatics. The advancement of high-throughput sequencing technology has led to the creation of extensive protein sequence databases [2–5], yet a notable proportion of these proteins lack functional annotations. Experimentally determining the functional properties of protein sequences is not only labor-intensive but also time-consuming [6]. In response to this challenge, a wide range of computational frameworks has been proposed for protein function annotation [7–10].

Traditional sequence-alignment based methods [11, 12] are utilized to transfer the functions from similar annotated sequences or functional domains to query sequences, assuming that proteins with similar sequences and structures tend to share congnate functions [13]. For instance, Blast [11] is a basic method to transfer annotations directly from homologous sequences with labeled protein functions, which cannot make confident prediction on proteins without annotated homologous sequences in the real scenarios.

Furthermore, machine learning–based methods are developed leveraging existing information, such as amino acid sequences [8,14–17], protein–protein interactions [9,18–22], evolutionary relations [23], experimentally resolved or predicted protein structures [10,24–27], literature [28] and aforementioned multisource information combined [29]. In general, the amino acid sequences are readily available, whereas other characteristics of proteins, such as protein–protein interactions and protein structures, are often confined to a limited subset of proteins. This has facilitated the emergence of a plethora of sequence-based methods. For example, TALE+ [16] utilizes protein sequence inputs jointly embedded with hierarchical function labels to enhance protein function prediction without taking structural information into consideration. Furthermore, DeepGOPlus [17] combines a deep convolutional neural network (CNN) model with sequence similarity-based predictions to forecast protein functions based solely on sequences. Given that that protein structures have a direct relationship with functions, leveraging protein structures for function prediction may have a natural advantage over these sequence-based methods. Among the notable structure-based methods, DeepFRI [10] pioneers the use of protein structures generated by homology modeling for reinforcement, achieving comparable performance with good interpretability. More importantly, I-TASSER-MTD [30] is explicitly designed to model the structures and functions of multidomain proteins. Notably, although some protein structures have not been experimentally resolved, tools like AlphaFold2 [31], RoseTTAFold [32], and ESMFold [33] have demonstrated remarkable success in protein structure prediction. Furthermore, Struct2GO [26] validates the hypothesis that AlphaFold-predicted structures could enhance protein function annotation performance. Additionally, NetGO 3.0 [34] has introduces a novel logistic regression (LR)–ESM component, building upon NetGO 2.0 [29], to improve large-scale functional annotations. HEAL [27] utilizes a hierarchical graph transformer integrated with graph contrastive learning to optimize the similarity between different views represented by the graph. However, these deep learning–based methods may rely to some extent on homology information of sequences and models, potentially compromising their ability to transfer protein function prediction information from known to unknown dissimilar sequences, resulting in less satisfactory performance. MetaGO [35] is proposed to predict Gene Ontology (GO) of nonhomologous proteins by integrating 3 complementary pipelines: global and local structure alignments, sequence and sequence-profile matches, and protein–protein interaction (PPI) network mapping. However, the quality of PPI network, including data noise and completeness, may influence the performance of function prediction to some extent. In conclusion, there are promising prospects for the development of highly generalized prediction frameworks that demonstrate superior performance on dissimilar target datasets compared to their source datasets.

To address the aforementioned challenges and formalize a generalized framework, we propose a novel domain adaptation approach named Graph Adversarial Learning with Alignment (GALA) for protein function prediction. To thoroughly explore protein structure and capture essential residues from diverse environments, we introduce a graph transformer with an attention mechanism for representation learning. The transformer first generates meta-node embeddings to interact with other residues, followed by aggregating node embeddings for better protein representations. To enhance generalizability, we introduce a domain discriminator conditioned on both representations and predictions, which is trained adversarially to minimize the discrepancy between the source and target domains in the embedding space. In addition, to improve the discriminability of protein representations, we generate label embeddings in the latent space and enforce source representations to approach their corresponding label embeddings compared to other embeddings. In this way, we can produce discriminative and domain-invariant protein representations for more accurate function prediction.

To evaluate the performance of GALA, we compare it with several baseline methods, including Blast [11], DeepGOPlus[17], TALE+ [16], DeepFRI [10], Struct2GO [26], and HEAL [27], across various settings. We retrain these models utilizing our partitioned training sets and then evaluate their performance on the protein test sets in 3 functional aspects: molecular function (MF), biological process (BP), and cellular component (CC). To demonstrate the efficiency of GALA, we derive 2 versions of the model: GALA-PDB and GALA. The former is trained with a subset of proteins, while the latter incorporates AlphaFold2-predicted protein structures into training. Our model has achieved outstanding performance across all 3 aspects. Furthermore, we evaluate their performance on distinct specificity GO terms, and GALA proves to be robust to GO terms with varying specificity, particularly for rare GO terms. On the test set of AlphaFold2-predicted protein structures, GALA significantly outperforms all other methods. What’s more, GALA demonstrates exceptional performance on the nonhomologous PDBch test set. Notably, GALA excels on the newly annotated test set, underscoring its robustness over time. More importantly, our method GALA showcases exceptional generalizability and interpretability in identifying critical residues, rending it highly suitable for protein function prediction in dissimilar sequences. Finally, we perform an ablation study on our method GALA to evaluate the contribution of each module.

## Methods

### Problem definition

We begin by outlining the problem setting and notations. Previous protein function prediction approaches [27] typically assume that both training and test samples originate from the same distribution, a condition that cannot be guaranteed when novel sequences are discovered in the real–world scenarios. Toward this end, we study a relatively underexplored but more practical setting of domain-adaptive protein function prediction. Here, we have access to a labeled source domain $\mathcal {D}^s=\lbrace (G_i^s,y_i^s)\rbrace _{i=1}^{n_s}$ with $n_s$ protein graphs and an unlabeled target domain $\mathcal {D}^t=\lbrace (G_j^t)\rbrace _{j=1}^{n_t}$ with $n_t$ graphs. $\mathcal {D}^s$ and $\mathcal {D}^t$ share the same label space, that is, $\mathcal {Y}=\lbrace 1,2,\dots ,C\rbrace$ with different distributions in the data space. Therefore, our objective is to minimize the discrepancy among diverse domains within the embedding space, thus enhancing the model’s generalizability and enabling the seamless transfer of protein function label information from the source domain to the target domain.

To characterize the spatial structure, we represent each protein using a graph $G = (\mathcal {V}, \mathcal {E})$, where $\mathcal {V}$ and $\mathcal {E}$ represent the node and edge sets, respectively. Specifically, the node set $\mathcal {V}$ comprises the amino acid residue sequence of a graph with $|\mathcal {V}|$ residues. Regarding the edge set, it is derived from the $C_{\alpha }$-$C_{\alpha }$ contact map. We define 2 amino acid residues as adjacent if the distance between their $C_{\alpha }$ atoms is less than $10 \, {\mathring{\rm A}}$. Subsequently, we add an edge between adjacent residues and construct an adjacency matrix $A \in \mathcal {R}^{|\mathcal {V}|\times |\mathcal {V}|}$. Additionally, the node feature matrix $X \in \mathcal {R}^{|\mathcal {V}|\times F}$ is obtained from 2 sources: (i) a one-hot residue encoder encoded by amino acid symbols and (ii) the ESM-1b protein language model [36], which produces residue embeddings to capture intrinsic protein sequence knowledge. These embeddings are then concatenated to form the feature matrix.

### An overview of the proposed GALA

**Figure 1:**
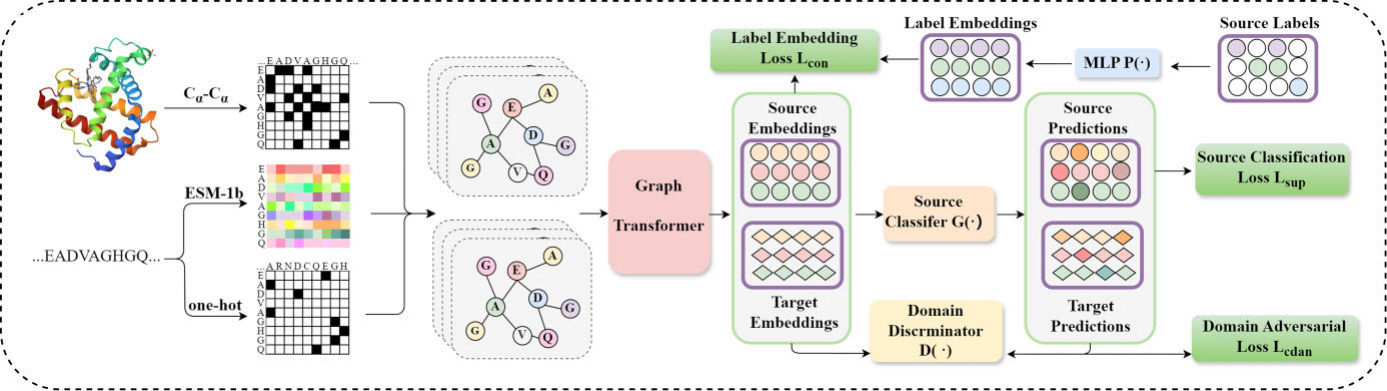
Overview of our proposed method GALA. GALA first adopts a GCN-based encoder to aggregate local niche information and obtain node-level feature embeddings for each graph. Subsequently, a multihead meta-nodes graph transformer is introduced to thoroughly explore protein structure, utilizing attention pooling module to aggregate graph-level representations. To better represent protein graphs, a 2-layer multilayer perception is applied to generate label embeddings in the latent space for labeled source data and then embed graph representations with label information for better function prediction. To enhance the generalizability of GALA, a domain adversarial discriminator is applied to narrow the discrepancy between source and target domains and align various domains with low sequence identity.

In this article, we propose a new approach named GALA for protein function prediction in dissimilar sequences. Our GALA utilizes the graph transformer to acquire graph-level embeddings that capture spatial semantics and essential information about key residues. In addition, a domain classifier is introduced conditioned on both representations and predictions, facilitating the acquisition of domain-invariant features by adversarial learning. Finally, label embedding alignment is adopted to enhance the discriminability of protein representations. For a more comprehensive understanding, please refer to the detailed information provided in Fig. 1.

### Graph transformer for representation learning

We first employ a graph convolutional network (GCN) to capture the overall structure of the graph [37, 38]. In this process, the node embeddings are gradually updated by aggregating information from the nodes’ neighborhood in the last layer. The embeddings are updated with the following layer-wise rule.


(1) \begin{eqnarray*}
H^{l+1}=\sigma (\tilde{D}^{-\frac{1}{2}}\tilde{A}\tilde{D}^{-\frac{1}{2}}H^{l}W^{l}).
\end{eqnarray*}

Here, $\tilde{A}$ is the adjacency matrix of protein graph $G$ with added self-loops. $\tilde{D}$ is the degree matrix and $W^l$ is a layer-specific weight matrix that can be learnable. Furthermore, $\sigma (\cdot )$ is an activation function and $ReLU(\cdot )=max(0,\cdot )$ is applied during training. $H^l$ is the embedding matrix in the $l$th layer and $H^0=X$. After $N$ layers, we generate the hidden embedding matrix $H\triangleq H^N\in \mathcal {R}^{|\mathcal {V}|\times D}$, where $D$ is the dimension of hidden embeddings. After applying the GCN, we generate node-level embeddings for each graph.

To effectively integrate residue neighborhood information and represent protein structural information, we are required to aggregate node-level representations for each graph. In particular, we introduce $K$ meta-nodes, denoted as learnable features $q_1,\dots ,q_K$, to interact with node embeddings and then capture the protein structure information. Inspired by the graph transformer [39], we obtain key and value embedding vectors $\mathcal {K}\in \mathcal {R}^{|\mathcal {V}|\times D}$ and $\mathcal {V}\in \mathcal {R}^{|\mathcal {V}|\times D}$ from another 2 graph convolution networks leveraging the graph structure, and the concatenated meta-node representation $\mathcal {Q}=(q_1,\dots ,q_k)\in \mathcal {R}^{K\times D}$ performs as query vector. We calculate the similarity between $\mathcal {Q}$ and $\mathcal {K}$ to obtain weights, which are then used to weight value vector $\mathcal {V}$, and finally derive the meta-node embedding matrix $\Gamma \in \mathcal {R}^{K\times D}$ using the following formula:


(2) \begin{eqnarray*}
\Gamma &= softmax\left(\frac{\mathcal {Q}\cdot \mathcal {K}^T}{\sqrt{D}}\right)\cdot \mathcal {V},
\end{eqnarray*}

(3) \begin{eqnarray*}
\mathcal {K}&= GCN^1(H,A),\quad \mathcal {V} = GCN^2(H,A).
\end{eqnarray*}

Instead of computing a single attention, we can further utilize multihead attention [40]. This involves repeating the above formula for $h$ times with distinct parameters, resulting in $h$ different representation subspaces, denoted as $\Gamma _1, \dots , \Gamma _h$. Subsequently, we concatenate these $h$ derived meta-node embeddings and transform them to a multihead meta-node embedding using a fully connected network. In other words,


(4) \begin{eqnarray*}
U = FC^1(\left[\Gamma _1,\dots ,\Gamma _h\right]),
\end{eqnarray*}

where $FC^1$ denotes a multilayer perceptron (MLP), and thus $U\in \mathcal {R}^{K\times D}$ is a multihead meta-nodes embedding matrix, which represents structure information in the protein graph.

To aggregate local niche information, we adopt an attention module, which summarizes these multihead meta-node representations into a graph-level representation in an adaptive fashion. Specifically, we utilize a query vector $\mathcal {Q}^P\in \mathcal {R}^D$ and 2 transformation matrices $\mathcal {K}^P\in \mathcal {R}^{D\times D}$ and $\mathcal {V}^P\in \mathcal {R}^{D\times D}$, and then the graph representation $z$ is derived by the following formula:


(5) \begin{eqnarray*}
z = softmax\left(\frac{\mathcal {Q}^P\cdot (U\cdot \mathcal {K}^P)^T}{\sqrt{D}}\right)\cdot U\cdot \mathcal {V}^P.
\end{eqnarray*}

Finally, we construct a source classifier $G$ to establish a projection between the graph representation $z$ and the label $y$, and the predicted positive probability is denoted as $\hat{y} = G(z)$. A binary cross-entropy loss objective function for multilabel classification of labeled source data is described as follows:


(6) \begin{eqnarray*}
\mathcal {L}_{sup}=-\frac{1}{M\cdot C}\sum \limits _{c=1}^C\sum \limits _{m=1}^M\left(y_{mc}log(\hat{y}_{mc})+(1-y_{mc})log(1-\hat{y}_{mc})\right).
\end{eqnarray*}

where *M* is the sample size of a mini-batch, and *C* is the number of classes. What’s more, $y_{mc}$ and $\hat{y}_{mc}$ denote the ground truth and predicted probability for the $c$th function of $m$th sample, respectively.

### Adversarial learning for domain alignment

Previous methods [10] typically overlook domain alignment to some extent, assuming that the source and target domains inherently share the same distribution. As a result, annotating novel proteins that differ from known functional proteins poses considerable challenges. The key to addressing this issue lies in minimizing the gap between the source and target domains and subsequently learn domain-invariant features to achieve cross-domain protein functional annotation. In this context, we introduce a domain discriminator to learn domain-invariant graph representations to transfer annotations from the source domain to the target domain.

Specifically, we leverage adversarial learning [41] to obtain domain-invariant graph representations, which can be formulated as an optimization problem involving source classifier $G$ and domain discriminator $D$ across the source and target domains. We randomly sample a mini-batch of $M$ graphs from source and target data, respectively, and a binary cross-entropy loss is employed to distinguish whether a sample is from the source domain or the target domain. Let $z$ and $\hat{y}$ denote the outputs of feature extractor $F$ and source classifier $G$, respectively. The adversarial learning loss is formulated as follows:


(7) \begin{eqnarray*}
\mathcal {L}_{adv}=&-\frac{1}{M}\sum \limits _{i=1}^Mw(H(\hat{y}_i^s))\log D(T(z_i^s,\hat{y}_i^s)) \\
&-\frac{1}{M}\sum \limits _{j=1}^Mw(H(\hat{y}_j^t))\log \left(1-D(T(z_j^t,\hat{y}_j^t))\right),
\end{eqnarray*}

where $z_i^s$ and $z_j^t$ denote graph embeddings of graph $i$ and graph $j$ from the source data and target data. Moreover, $\hat{y}_i^s$ and $\hat{y}_j^t$ are predicted probabilities of graphs, and $D$ denotes domain discriminator. $T(z_i^s, \hat{y}_i^s) = \frac{1}{\sqrt{d}}(R_z z_i^s) \odot (R_{\hat{y}} \hat{y}_i^s)$ represents the explicit randomized multilinear map of dimension $d$. Here, $\odot$ denotes the element-wise product, and $R_z$ and $R_{\hat{y}}$ are random matrices sampled only once and held constant throughout training. Each element $R_{i,j}$ follows a symmetric distribution with univariance. The mapping is used to capture multiplicative interactions between feature representation and classifier prediction, which is important to learn domain-invariant features. The entropy-aware weight, denoted as $w(H(\hat{y}_i^s)) = 1 + e^{-H(\hat{y}_i^s)}$, is employed to adjust the weights of samples. This aims to prioritize the discriminator’s focus on examples that are easier to transfer, as indicated by more certain predictions. Through adversarial learning, we align source and target domains, which is beneficial for obtaining domain-invariant representations and consequently improving model generalizability.

### Label embedding alignment

Inspired by TALE [16], we introduce a label embedding alignment module, which first generates label embeddings for source data in the representation space and then aligns graph and label embeddings in the hidden space, aiding the learning of semantics from labeled source data. Specifically, we employ a 2-layer MLP $P(\cdot )$ to project each label representation $y$ into a label embedding $b$ with the same dimension as $z$ (i.e., $b = P(y)$). The subsequent goal is to align $z$ and $b$ for each protein, so that label information is contained in graph embedding. Comprehensively, we sample a mini-batch of $M$ proteins, each of which produces graph embedding $z$ and label embedding $b$, and then the loss function for label embedding alignment is written as follows:


(8) \begin{eqnarray*}
\mathcal {L}_{con}=-\frac{1}{M}\sum \limits _{i=1}^{M} log\frac{e^{z_i^s*b^s_i/\tau }}{\sum \limits _{j\ne i} e^{z_i^s*b^s_j/\tau }},
\end{eqnarray*}

where $\tau$ denotes a temperature parameter and $*$ calculates the cosine similarity between graph and label embeddings of labeled source data. After the alignment, we can effectively generate protein embeddings that enrich label information.

### Total loss and model training

The final loss function is derived by combining the above losses as


(9) \begin{eqnarray*}
\mathcal {L} = \mathcal {L}_{sup} + \mathcal {L}_{adv} + \mathcal {L}_{con}.
\end{eqnarray*}

We train the proposed model using Adam [42] with learning rate 1e-4 and adopt SGD to train the domain discriminator with momentum 0.9 and learning rate 0.03. All modules are trained utilizing a single A100-PCIE 80 GB graphics processing unit (GPU), with training times of approximately 2 hours using a batch size of 64. Moreover, the running times for several protein cases are provided in Supplementary Table S6.

## Dataset

In our experiments, we utilize the same dataset, named PDBch, from DeepFRI [10] work, which consists of 36,641 experimentally solved protein structures from the PDB database [2] and their associated GO terms sourced from SIFTS [43]. To ensure dissimilarity between our training and test sets, we employ the MMseqs [44] sequence clustering tool with a sequence identity threshold of 30%. What’s more, the training, validation, and test sets are then selected from different clusters, with an approximate ratio of 8:1:1, ensuring that the sequence identity between samples from different sets is below 30%. While the sequence identity among different sets is low, the pivotal issue we are addressing is the transfer of GO terms from the training set to the test set. After acquiring the sets, we proceed to assign functional labels to each protein sequence based on the GO terms compiled by Gligorijević et al. [10]. These functional labels are categorized into 3 distinct groups: MF, BP, and CC [45]. Each category serves as an independent prediction task during the training process.

We conduct additional experiment to assess whether recent advancements in protein structure prediction contribute to enhancing domain adaptation. Gligorijević et al. [10] construct the SMch dataset through collecting homology models of the PDBch dataset with at least 1 annotation from the SWISS-MODEL repository [46]. Following Gu et al. [27], we select 41,997 proteins from the SMch dataset with low-frequency GO terms (proteins with information content [IC] >10 from the PDBch dataset) and retrieve their structures predicted by AlphaFold2 (AF2) from the AlphaFold protein structure database [31]. When selecting a portion from the SWISS-MODEL repository to construct the AFch set, we take into account the highly imbalanced distributions of GO term labels in the PDBch set. For low-frequency GO terms in the PDBch set, we specifically choose proteins with the corresponding GO term labels from the SWISS-MODEL respository to form the AFch set. This approach helps mitigate the imbalance in PDBch labels to some extent. Detailed information about datasets can be found at Table 1 and Supplementary Section 1.

**Table 1:** Number of sequences in the datasets

	Number of sequences		
Datasets	Training set	Validation set	Test set
PDBch set	29,304	3,660	3,665
AFch set	34,135	3,881	3,981

Utilizing the frequency of each GO term in the combined training set (PDBch and AFch), we compute the IC for each GO term within this set. Higher information content indicates a more specialized GO term.


(10) \begin{eqnarray*}
IC(GO_{i})=-\log _2(P(GO_i)).
\end{eqnarray*}

## Evaluation metrics

Protein function prediction poses a significant challenge as it involves a highly imbalanced multilabel classification task. Complicating matters, the GO terms associated with these functions are not merely juxtaposed; instead, they exhibit a directed acyclic graph structure. To address the inherent bias and acknowledge the intricate relationships between these terms during model evaluation, we adopt several metrics proposed by the critical assessment of functional annotation (CAFA) algorithms challenge [13]. These metrics are widely acknowledged and utilized to assess the performance of protein function prediction models.

The first metric is function-centric AUPR, which is calculated by averaging the sum of all $AUPR_f$, where $f$ denotes a certain GO term. AUPR can be formulated as follows:


(11) \begin{eqnarray*}
AUPR_f&=\sum \limits _{t}(rc_f(t)-rc_f(t-1))\times pr_f(t),
\end{eqnarray*}

(12) \begin{eqnarray*}
AUPR&=\frac{1}{N_f}\sum \limits _f AUPR_f,
\end{eqnarray*}

where $t$ is a cutoff value ranging from 0 to 1 with step size 0.01, $rc_f$ and $pr_f$ represent recall and precision score for the GO term $f$. Furthermore, $N_f$ is the number of GO terms.

The second metric is protein-centric $F_{max}$, which is defined as


(13) \begin{eqnarray*}
AvgPr(t)&=\frac{1}{m(t)}\sum \limits _{i=1}^{m(t)}pr_i(t),
\end{eqnarray*}

(14) \begin{eqnarray*}
AvgRc(t)&=\frac{1}{n}\sum \limits _{i=1}^{n}rc_i(t),
\end{eqnarray*}

(15) \begin{eqnarray*}
F_{max}&=\underset{t}{\text{max}}\left\lbrace \frac{2\cdot AvgPr(t)\cdot AvgRc(t)}{ AvgPr(t)+AvgRc(t)}\right\rbrace ,
\end{eqnarray*}

where $m(t)$ is the number of proteins on which at least 1 prediction is made above threshold $t$, $rc_i(t)$ and $pr_i(t)$ represent recall and precision score of protein $i$ at threshold $t$, and $n$ is the number of proteins.

Given the highly imbalanced nature of the GO term labels, we introduce the Matthews correlation coefficient (MCC), computed under a threshold that yields a maximum protein-centric measure $F_{max}$ [8]. MCC value is calculated as follows:


(16) \begin{eqnarray*}
MCC=\frac{TP\cdot TN - FP\cdot FN}{\sqrt{(TP+FP)(TP+FN)(TN+FP)(TN+FN)}},
\end{eqnarray*}

where TP is the number of true positives, FN is the number of false negatives, FP is the number of false positives, and TN is the number of true positives.

Additionally, we propose $S_{min}$ to denote the semantic distance between predicted and true annotations, considering information content of GO terms. $S_{min}$ is computed using the following formula:


(17) \begin{eqnarray*}
S_{min}=\underset{\text{t}}{\min }\sqrt{ru(t)^2+mi(t)^2},
\end{eqnarray*}

where $ru(t)$ represents the average uncertainty under threshold *t* and $mi(t)$ is the average misinformation:


(18) \begin{eqnarray*}
ru(t)&=\frac{1}{n}\sum \limits _{i=1}^n\sum \limits _{c\in T_i-P_i(t)} IC(c|Pa(c)),
\end{eqnarray*}

(19) \begin{eqnarray*}
mi(t)&=\frac{1}{n}\sum \limits _{i=1}^n\sum \limits _{c\in P_i(t)-T_i} IC(c|Pa(c)).
\end{eqnarray*}

Here, $T_i$ and $P_i(t)$ represent the ground truth and predicted labels for protein *i*, respectively. Also, $Pa(c)$ represents the parents of term *c*, and the information content is computed based on the posterior probability of term *c*, that is, $IC(c|P(c))=-\log (P(c|Pa(c)))$.

## Results

To thoroughly assess the efficiency of our proposed method, GALA, we conduct a comprehensive comparison with several baseline methods, which include a sequence alignment-based method (Blast) and 5 deep learning–based methods (DeepGOPlus, TALE+, DeepFRI, Struct2GO, and HEAL) under several test sets. To facilitate differentiation, the model obtained using our method trained solely on the PDBch training set is called GALA-PDB, while the model trained on both the PDBch and AFch training sets is called GALA. Given the lower sequence identity between the training and test sets, our objective is to evaluate the performance of these methods when training and test sets are dissimilar. For a fair comparison, all the compared methods are retrained on both the PDBch and AFch training sets. Table 2 presents the training set and input information for the compared methods. Subsequently, their performance is evaluated on our designated sets.

**Table 2:** Several baseline methods for protein function prediction

Methods	Training set	Input information
Blast	—	Sequence
DeepGOPlus	PDBch + AFch training set	Sequence
TALE+	PDBch + AFch training set	Sequence
DeepFRI	PDBch + AFch training set	Sequence and Structure
Struct2GO	PDBch + AFch training set	Sequence and Structure
HEAL	PDBch + AFch training set	Sequence and Structure
GALA-PDB	PDBch training set	Sequence and Structure
GALA	PDBch + AFch training set	Sequence and Structure

### Performance of protein function prediction and domain adaptation

**Figure 2:**
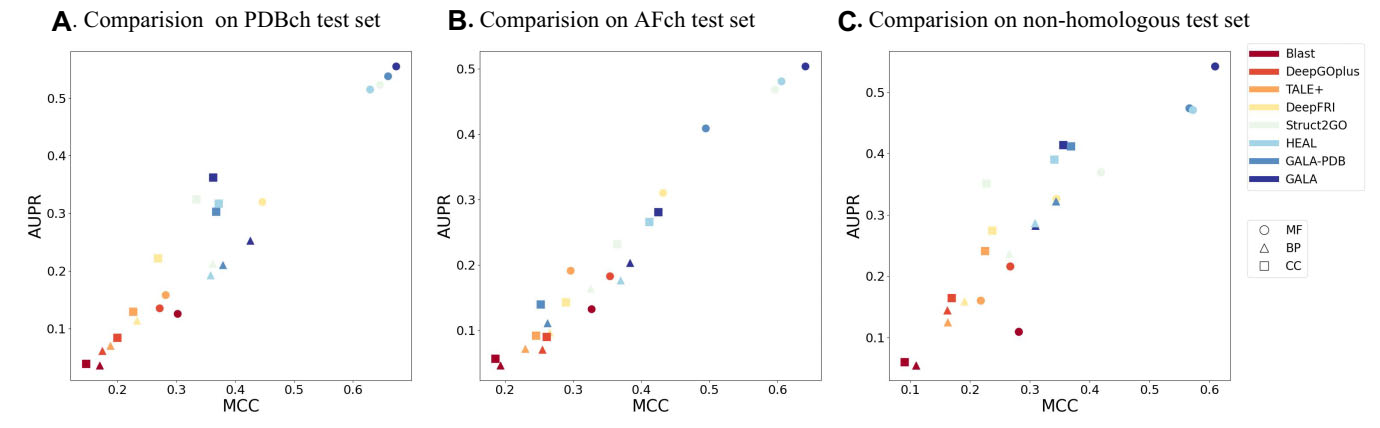
Comparison of GALA with several baseline methods based on 2 pairwise metrics, AUPR and MCC. In these figures, squares represent testing on MF GO terms, triangles represent testing on BP GO terms, and circles represent testing on CC GO terms. The left subfigure (A) shows the comparison between GALA and baseline methods on the PDBch test set, the subfigure (B) presents the comparison on the AFch test set, and the right subfigure (C) illustrates the comparison on the nonhomologous PDBch test set.

Table 3 provides a summary of the overall results for all 6 protein prediction methods across 3 GO domains (MF, BP, and CC), and we show the best performance in bold and the second best performance underlined for better comparison. As we train GALA solely with the PDBch training test, we name it GALA-PDB. GALA-PDB achieves AUPR scores of 0.5386, 0.2104, and 0.3032 and $F_{max}$ scores of 0.6710, 0.5519, and 0.3712 on MF, BP, and CC tasks, respectively. GALA-PDB performs exceptionally well in MF and BP terms, outperforming Blast and other deep learning–based methods, including DeepGOPlus, TALE+, DeepFRI, Struct2GO, and HEAL, in terms of AUPR, $F_{max},S_{min}$, and MCC. However, it shows slightly lower performance than Struct2GO and HEAL on the CC task, while still yielding comparable results. Despite the smaller training set and less available information to the model for GALA-PDB, it performs on par with other methods in areas such as feature extraction, protein function prediction, and domain knowledge transfer. In fact, it may even outshine other methods slightly. When incorporating the AFch training set into the training process, the resulting GALA model achieves AUPR scores of 0.5553, 0.2529, and 0.3625, as well as $F_{max}$ scores of 0.6730, 0.5833, and 0.3854, for 3 GO prediction tasks, respectively. Furthermore, it demonstrates superior performance compared to GALA-PDB and comprehensively outperforms other baseline methods in predicting 3 different GO tasks, assessed through 4 distinct evaluation metrics AUPR, $F_{max},S_{min}$, and MCC. As shown in Fig. 2A, 2 versions of our method, GALA and GALA-PDB, exhibit superior performance compared to other baseline methods when tested on all GO terms.

**Table 3:** Comparison of our model with several baseline methods on the PDBch test set^a^

	AUPR ($\uparrow$)			$F_{max}$ ($\uparrow$)			Smin ($\downarrow$)			MCC ($\uparrow$)		
Methods	MF	BP	CC	MF	BP	CC	MF	BP	CC	MF	BP	CC
Blast	0.1260	0.0363	0.0395	0.4304	0.3815	0.2087	2.5372	10.4218	1.3022	0.3025	0.1701	0.1467
DeepGOplus	0.1356	0.0618	0.0844	0.3844	0.3810	0.3034	2.3199	10.1569	1.2450	0.2716	0.1742	0.1999
TALE+	0.1584	0.0701	0.1299	0.3834	0.3784	0.3152	2.3258	10.0628	1.1999	0.2820	0.1885	0.2268
DeepFRI	0.3206	0.1144	0.2225	0.4923	0.4274	0.3249	1.9660	9.7596	1.1112	0.4460	0.2332	0.2688
Struct2GO	0.5234	0.2124	0.3248	0.6417	0.5376	0.3741	1.4829	8.7021	0.9759	0.6453	0.3615	0.3344
HEAL	0.5156	0.1924	0.3176	0.6376	0.5218	0.3830	1.5213	9.1056	0.9892	0.6288	0.3582	**0.3715**
GALA-PDB	0.5386	0.2104	0.3032	0.6710	0.5519	0.3712	**1.4134**	8.8459	1.0193	0.6592	0.3794	0.3676
GALA	**0.5553**	**0.2529**	**0.3625**	**0.6730**	**0.5833**	**0.3854**	1.4212	**8.3964**	**0.9343**	**0.6730**	**0.4253**	0.3621

^a^Bold indicates the best performance, while the underlining represents the second best performance.

Evidently, we observe that our method significantly enhances protein function prediction when compared to several state-of-the-art methods. It demonstrates strong performance even when the sequence identity between the training and test sets is minimal. What’s more, it adeptly transfers information from the source domain, composed of the training set, to the target domain, which consists of the test set that is less similar to the source domain. The generalization ability of our method GALA is reflected to some extent.

### Performance on GO terms with different specificity

The protein function prediction task is indeed a multiclassification problem, with MF comprising 489 terms, BP comprising 1,943 terms, and CC comprising 320 terms. It is imperative to address the issue of class imbalance, which can potentially lead to misleading classifications, particularly in deep learning–based methods. In the Dataset section, we introduce information content metric to assess the specificity of different GO terms, representing the rarity of each GO term. Combining MF, BP, and CC terms, we apply a categorization based on information content, utilizing thresholds of 5 and 10. This categorization leads to the division of GO terms into 3 groups: IC < 5, 5 < IC < 10, and IC > 10. More importantly, this stratification allows for a more nuanced analysis of predictive performance across GO terms with varying degrees of specificity.

**Figure 3:**
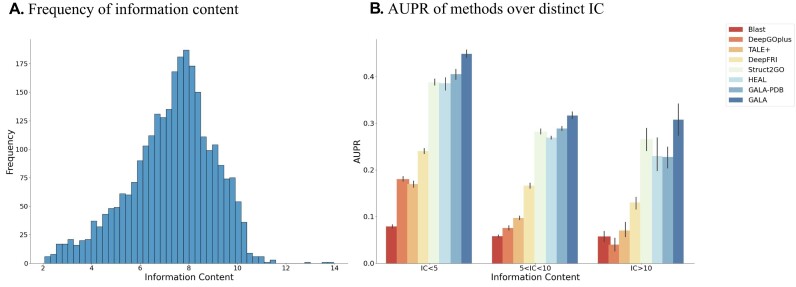
The left subfigure (A) shows the frequency of information content (IC) for protein functions over collection of 3 categories (MF, BP, and CC) in the combination of the PDBch training set and AFch training set. The right subfigure (B) shows AUPR of different methods over distinct IC.

As shown in Fig. 3, the left subfigure illustrates the distribution of information content in the combination of PDBch and AFch training sets. Simultaneously, the right subfigure depicts the performance of different methods across 3 categories of GO terms, utilizing 10 bootstrap iterations on all test proteins. As the information content value increases, all methods exhibit a consistent downward trend. For commonly occurring terms (IC < 5), GALA and DeepFRI achieve average AUPR scores of 0.4396 and 0.2330, respectively. In the mid-range terms (5 < IC < 10), GALA and DeepFRI attain average AUPR scores of 0.3269 and 0.1668, respectively. Meanwhile, on highly specific terms (IC > 10), GALA and DeepFRI achieve average AUPR scores of 0.2885 and 0.1309, respectively. Overall, GALA outperforms all other methods significantly across the 3 categories of GO terms. Moreover, as GO terms become more specific, GALA exhibits a much slower decrease in performance compared to other methods (Supplementary Section 2). Indeed, this observation to a certain extent demonstrates the robustness and superiority of GALA in predicting specific GO terms, aligning with our requirements for accurate and nuanced predictions in the context of protein function prediction.

### Performance on AlphaFold2-predicted structures

For proteins with experimentally resolved structures or highly similar annotated proteins, predicting their functions is relatively straightforward. Therefore, our focus and practical significance lie in proteins with unknown structures that lack homology to existing proteins. Extending functional annotation from well-characterized protein domains to those that are unknown can significantly impact the discovery and understanding of novel proteins in biomedicine and other fields. To assess the model’s generalization ability, particularly its performance on proteins with unknown structures, we conduct experiments on the AFch test set whose structures are predicted by AlphaFold2. This set serves as a valuable measure of the model’s capability to generalize.

**Figure 4:**
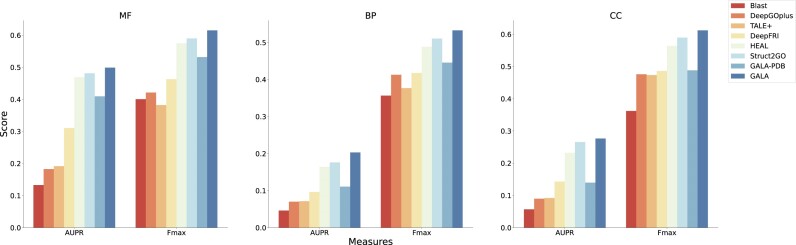
The figure shows AUPR and $F_{max}$ scores of different methods on the AFch test set. GALA outperforms all other methods significantly in molecular function (MF), biological process (BP), and cellular component (CC) tasks.

During the dataset preprocessing stage, we selectively remove all protein sequences from the AFch test set with a sequence identity of more than 30% with the combined training set (comprising PDBch and AFch training sets). As illustrated in Fig. 4, GALA outperforms all other methods significantly in MF, BP, and CC tasks. The AUPR scores for MF, BP, and CC are 0.5040, 0.2034, and 0.2813, respectively, while the corresponding $F_{max}$ scores are 0.6140, 0.5340, and 0.6133 (Supplementary Table S5). The results are depicted in Fig. 2B and Fig. 4, highlighting the effectiveness of our approach. Notably, GALA-PDB performs poorly due to the exclusion of the AFch set during training, while GALA demonstrates superior performance compared to other methods.

### Performance on nonhomologous proteins

Given that protein structures diverge much more slowly than sequences, certain proteins with dissimilar sequences can still possess similar structures, indicating distant homology [47]. Furthermore, we utilize both sequence identity and TM-score to identify nonhomologous proteins in the PDBch test set. Specifically, proteins in the PDBch test set with a sequence identity of less than 30% and a TM-score [48, 49] below 0.5 from sequences in the PDBch training set are classified as nonhomologous. This stricter test set is referred to as the nonhomologous PDBch test set. The performance comparison of GALA and several baseline methods are shown in Table 4. Notably, GALA demonstrates exceptional performance on the nonhomologous PDBch test set. Furthermore, as illustrated in Fig. 2C, GALA outperforms several baseline methods on the nonhomologous PDBch test set, based on the pairwise metrics AUPR and MCC.

**Table 4:** Comparison of our model with several baseline methods on the nonhomologous PDBch test set^a^

	AUPR ($\uparrow$)			$F_{max}$ ($\uparrow$)			Smin ($\downarrow$)			MCC ($\uparrow$)		
Methods	MF	BP	CC	MF	BP	CC	MF	BP	CC	MF	BP	CC
Blast	0.1095	0.0545	0.0601	0.3045	0.2417	0.1955	2.7853	10.5673	1.1936	0.2815	0.1099	0.0909
DeepGOplus	0.2159	0.1445	0.1646	0.3638	0.3014	0.3001	2.6467	10.3173	1.1699	0.2674	0.1620	0.1695
TALE+	0.1602	0.1246	0.2413	0.3100	0.2832	0.3227	2.6626	10.3933	1.1678	0.2175	0.1624	0.2251
DeepFRI	0.3264	0.1588	0.2749	0.3761	0.3188	0.3131	2.4628	9.9593	1.1309	0.3441	0.1908	0.2368
Struct2GO	0.3694	0.2362	0.3511	0.4554	0.3674	0.3690	2.2184	9.5941	**1.0258**	0.4187	0.2656	0.2274
HEAL	0.4709	0.2868	0.3905	0.5024	0.4093	0.3843	**1.9050**	9.4427	1.0462	0.5719	0.3085	0.3410
GALA-PDB	0.4742	**0.3221**	0.4121	**0.5147**	0.4213	0.3734	1.9398	9.1878	1.0344	0.5667	**0.3437**	**0.3685**
GALA	**0.5423**	0.2821	**0.4142**	0.5082	**0.4385**	**0.3951**	1.9259	**9.0331**	1.0599	**0.6092**	0.3091	0.3558

^a^Bold indicates the best performance, while the underlining represents the second best performance.

### Performance on proteins newly annotated in the Swiss-Prot database since 2021

**Figure 5:**
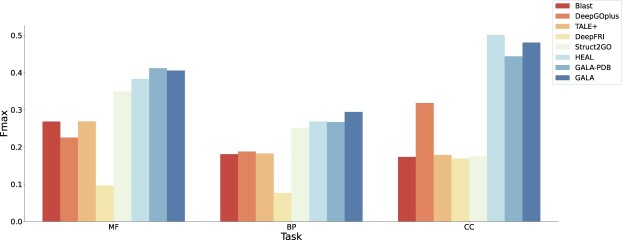
The figure shows $F_{max}$ scores of different methods on the test set, composed of newly annotated proteins in the Swiss-Prot database since 2021.

Conducting temporal validation is crucial as it provides a comprehensive assessment of our method’s robustness over time. Specifically, we collect proteins newly annotated and reviewed in the Swiss-Prot database [4, 50] between January 2021 and June 2024, resulting in a total of 7,799 proteins. We then select proteins with sequence identity less than 30% of those in the combined PDBch and AFch training set, forming a newly annotated test set comprising 2,270 proteins with predicted structures in the AlphaFold database. Consequently, we evaluate the performance of different methods on this newly annotated test set, as shown in Fig. 5. Significantly, GALA exhibits outstanding performance on the newly annotated test set, confirming its robustness over time.

### Key residues identification and analysis

To demonstrate the biological interpretability of GALA, we employ Grad-CAM [51] to identify the key residues contributing to the corresponding GO annotation function, effectively discerned by GALA. In our context, we utilize the output of the final graph convolution layer represented as $F\in \mathcal {R}^{L\times D}$, where $L$ denotes the number of protein residues and $D$ is the dimension of the feature space, as the feature map for this purpose. Then we take the derivative of the protein function $y_{l}$ with respect to $F$ as the gradient weight $W^l_{i,j}$:


(20) \begin{eqnarray*}
W^l_{i,j}=\frac{\partial y_l}{\partial F_{i,j}}
\end{eqnarray*}

The contribution score of the $i$th residue to the $l$th function $CAM^l_i$ can be obtained as


**Figure 6:**
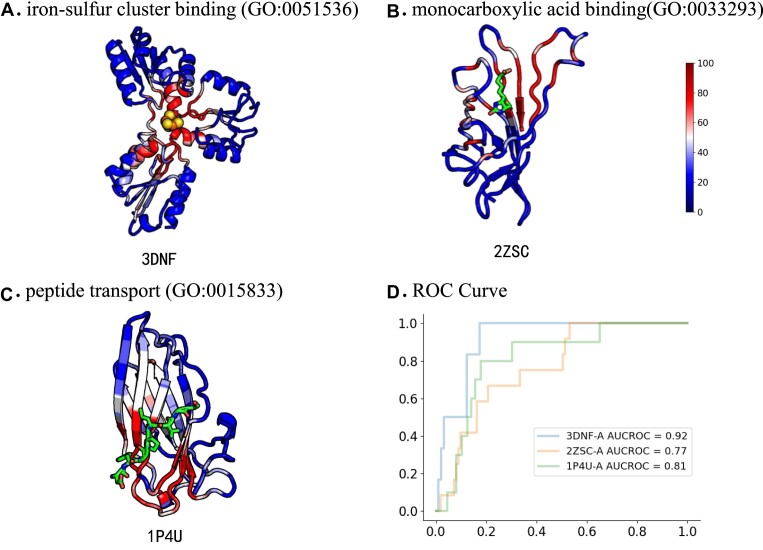
Four examples of the Grad-CAM activation profiles mapped onto the experimentally solved structures. (A), (B), and (C) are protein structures colored by the contribution scores computed by Grad-CAM. (D) ROC curves indicate that contribution scores computed by Grad-CAM overlap with binding sites retrieved from the BioLiP database.

(21) \begin{eqnarray*}
CAM^l_i=Relu(\frac{\sum _{j=1}^DW^l_{i,j}\cdot F_{i,j}}{D})
\end{eqnarray*}

which is subsequently normalized to fall within the range of 0 to 100, and then we generate heatmaps to illustrate the contribution scores. Furthermore, we project the heatmap onto the protein structure and observe sites with a strong signal, as depicted in Fig. 6.

For MF-GO terms, we provide 2 cases where the generated heatmaps align with experimentally confirmed binding sites. In the first example, 3DNF (Fig. 6 A, Supplementary Fig. S2), a protein associated with the function of iron–sulfur cluster binding (GO:0051536), exhibits strong signals in key residues binding with the iron–sulfur cluster. The second example, 2ZSC (Fig. 6 B, Supplementary Fig. S3), a protein involved in monocarboxylic acid binding (GO:0033293), reveals regions of strong signal surrounding its binding sites. For BP-GO terms, an example is presented, namely, 1P4U (Fig. 6C, Supplementary Fig. S4), with the function of peptide transport (GO:0015833). The residues of 1P4U within the peptide binding interface demonstrate a significant Grad-CAM signal.

We proceed to extract the binding sites of the 3 proteins from the BioLiP database [52]. What’s more, we compare the high-contribution residues identified by Grad-CAM with those experimentally verified in the binding sites. As illustrated in Fig. 6D, the area under the receiver operating characteristic curve (AUC-ROC) demonstrates that our model possesses an excellent capability to capture functional residues, providing strong evidence for its biological interpretability.

### Ablation study

To investigate the effectiveness of various modules in GALA to its enhanced performance, we design an ablation study. In this study, we systematically introduce various modules incrementally to construct advanced models. Specifically, we denote the models as M1 (corresponding to GALA-PDB), M2, M3, M4, and M5 (representing the complete GALA model). This experimental design allows us to analyze and understand the contribution of each module to the overall improvement in performance.

In M1, the PDBch set is solely employed as the training set. The network undergoes training with domain adaptation from the source domain to the target domain, coupled with the utilization of contrastive loss, which aligns the protein embedding with the label embedding in the latent space. The key distinction between M1 and M5 lies in whether the AFch set is included in the training process. Moving on to M2, this model is trained with the combined set of PDBch and AFch without domain alignment module and label embedding alignment. Additionally, M3 incorporates transfer loss from source data to target data on the basis of M2. Building upon M3, M5 introduces alignment between protein and label embeddings from labeled source data, which are implemented on the foundation of M3, further refining the model’s ability to capture and transfer information across different domains.To demonstrate the effectiveness of domain alignment module, we exclude the adversarial loss from training, as compared to M5.

Table 5 presents AUPR, $F_{max},S_{min}$ values for 5 models across 3 GO aspects on the PDBch test set. Upon comparing M1 and M5, the significance of incorporating the AFch set into model training becomes evident, resulting in improved performance across all 3 GO terms. This observation suggests that the protein structures predicted by AlphaFold2 can enhance the efficiency of protein function prediction. Further comparisons between M2, M3, M4, and M5 reveal a progressive enhancement in the performance of GALA. Notably, the inclusion of the domain alignment module and the protein-label embedding alignment module contribute to this improvement, which can be proved by experimental results on the MF and BP aspects. While we acknowledge potential conflicts in the Fmax score for predicting CC terms across different methods, we posit that AUPR and Smin metrics for CC terms validate the utility of our diverse modules. Moreover, the 3 metrics employed for predicting MF and BP terms collectively underscore the comprehensive effectiveness of those modules.

**Table 5:** Ablation study of GALA on PDBch test set^a^

	Modules			AUPR($\uparrow$)			$F_{max}(\uparrow$ )			$S_{min}(\downarrow$ )		
	AFch	adv	cl	MF	BP	CC	MF	BP	CC	MF	BP	CC
M1		✓	✓	0.5386	0.2104	0.3032	0.6710	0.5519	0.3712	1.4134	8.8459	1.0193
M2	✓			0.5269	0.1903	0.3160	0.6426	0.5292	0.3925	1.5131	8.9711	0.9875
M3	✓	✓		0.5338	0.2043	0.3207	0.6522	0.5372	0.3911	1.4947	8.9335	0.9866
M4	✓		✓	0.5482	0.2387	0.3234	0.6692	0.5736	0.3800	1.4662	8.4983	0.9682
M5	✓	✓	✓	0.5553	0.2529	0.3625	0.6730	0.5833	0.3854	1.4212	8.3964	0.9343

^a^AFch, adv, and cl correspond to training with the AFch set, adversarial learning for domain alignment, and label embedding alignment.

## Discussion

In this study, we have proposed GALA for protein prediction and generalization to proteins with dissimilar sequences, leveraging both sequence and structural data as input. For proteins that diverge from known counterparts and lack experimentally resolved structures, utilizing AlphaFold2 to predict structures and then feeding them into our model aligns seamlessly with real-world scenarios. GALA employs adversarial learning and label embedding alignment to extract domain-invariant representations, thereby augmenting the model’s generalization ability. Notably, the model outperforms several state-of-the-art methods, showcasing superior generalization capabilities to novel proteins dissimilar to known ones. GALA also demonstrates the close relationship between protein functions and pivotal residues, accentuating the interpretability and generalization ability of our model.

Moving ahead, we plan to incorporate the hierarchical directed acyclic structure of GO terms to refine the training process, while many methods take an extra postprocessing step during model evaluation to prevent hierarchy violations. Furthermore, as sequencing technology advances and protein structure–related methods develop, an increasing amount of protein-related information will become accessible. The integration of protein–protein interactions into the model can offer richer information for protein functional annotation, thereby enhancing the overall robustness and generalizability of the model.

## Additional Files

**Supplementary Files**. (1) Detailed information about the construction of datasets, (2) description of several baseline methods, (3) AUPR comparison for GO terms on the PDBch test set, (4) performance on the PDBch test set under a different specificity, (5) performance on the AFch test set, (6) runtime for several cases, and (7) plots for interpretability of key residues.

**Supplementary Fig. S1**. Frequency of IC for protein functions over collection of 3 categories (MF, BP, and CC) in the combination of the PDBch training set.

**Supplementary Fig. S2**. Contribution score computed by Grad-CAM of protein 3DNF with function of iron–sulfur cluster binding (GO:0051536).

**Supplementary Fig. S3**. Contribution score computed by Grad-CAM of protein 2ZSC with function of monocarboxylic acid binding (GO:0033293).

**Supplementary Fig. S4**. Contribution score computed by Grad-CAM of protein 1P4U with function of peptide transport (GO:0015833).

**Supplementary Table S1**. AUPR comparison for MF-GO terms on the PDBch test set.

**Supplementary Table S2**. AUPR comparison for BP-GO terms on the PDBch test set.

**Supplementary Table S3**. AUPR comparison for CC-GO terms on the PDBch test set.

**Supplementary Table S4**. Performance of GALA and other baseline methods on the PDBch test set under a different specificity.

**Supplementary Table S5**. Performance of GALA and other baseline methods on the AFch test set.

**Supplementary Table S6**. The running times for several cases.

giae093_Supplementary_Files

giae093_GIGA-D-24-00109_Original_Submission

giae093_GIGA-D-24-00109_Revision_1

giae093_GIGA-D-24-00109_Revision_2

giae093_GIGA-D-24-00109_Revision_3

giae093_Response_to_Reviewer_Comments_Original_Submission

giae093_Response_to_Reviewer_Comments_Revision_1

giae093_Response_to_Reviewer_Comments_Revision_2

giae093_Reviewer_1_Report_Original_Submission Xiaogen Zhou -- 5/7/2024

giae093_Reviewer_1_Report_Revision_1 Xiaogen Zhou -- 7/16/2024

giae093_Reviewer_2_Report_Original_Submission Amarda Shehu -- 5/11/2024

giae093_Reviewer_2_Report_Revision_1 Amarda Shehu -- 8/12/2024

giae093_Reviewer_3_Report_Original_Submission Cen Wan, Ph.D. -- 5/13/2024

giae093_Reviewer_3_Report_Revision_1 Cen Wan, Ph.D. -- 7/17/2024

## Abbreviations

AUC-ROC: area under the receiver operating characteristic curve; BP: biological process; CAFA: critical assessment of functional annotation; CC: cellular component; CNN: convolutional neural network; GALA: Graph Adversarial Learning with Alignment; GCN: graph convolutional network; GO: Gene Ontology; GPU: graphics processing unit; LR: logistic regression; MCC: Matthews correlation coefficient; MF: molecular function; MLP: multilayer perceptron; PPI: protein–protein interaction.

## Data Availability

Supporting datasets for this article are sourced from DeepFRI [10]. The first dataset, named PDBch, is selected from the PDB database and clustered using MMseqs [53] at a sequence identity of 30%. The training, validation, and test sets are chosen from different clusters with an approximate ratio of 8:1:1. As for the second dataset, AFch, we initially select 41,997 proteins from SWISS-MODEL and then partition them into training, validation, and test sets similar to the PDBch dataset. For more detailed information, refer to the Dataset section. An archival copy of the code and other data further supporting this work are openly available in the *GigaScience* repository, GigaDB [54]. Furthermore, a link to DOME-ML (Data, Optimization, Model, and Evaluation in Machine Learning) annotations is available via GigaDB [54].

## Availability of Supporting Source Code and Requirements

- Project name: GALA
- Project homepage: https://github.com/fuyw-aisw/GALA
- Operating system(s): Platform independent
- Programming language: Python
- Other requirements: not applicable
- License: MIT license
- RRID: SCR_025194
- Docker package: fuyw99/gala
