# Supplementary material for: Learning a generalized graph transformer for protein function prediction in dissimilar sequences
Source: Gigascience. 2024 Dec 5;13:giae093. doi: 10.1093/gigascience/giae093 (PMC11734293; doi:10.1093/gigascience/giae093)
Supplement: giae093_Supplementary_Files [file giae093_supplementary_files.zip › GALA_SI_revision_3.pdf]

# Supplementary Materials - Learning A Domain Adaptive Framework for Protein Function Prediction

Yiwei Fu [1,†], Zhonghui Gu [2,†], Xiao Luo [3,], Luhua Lai [2,4,*], and Minghua Deng[1,4,5,*]

[1]School of Mathematics Sciences, Peking University, Beijing, 100871, China
[2]Peking-Tsinghua Center for Life Sciences, Peking University, Beijing, 100871, China
[3]Department of Computer Science, University of California, Los Angeles, 90024, USA
[4]Center for Quantitative Biology, Peking University, Beijing, 100871, China
[5]Center for Statistical Science, Peking University, Beijing, 100871, China
E-mail: dengmh@pku.edu.cn or lhlai@pku.edu.cn

## 1 Dataset

In our experiments, we utilize the same dataset, named PDBch, from DeepFRI (Gligorijević *et al.*, 2021) work, which consists of 36,641 experimentally solved protein structures from the PDB database (Berman *et al.*, 2000) and their associated Gene Ontology (GO) terms sourced from SIFTS (Dana *et al.*, 2019). All protein chains in the PDB database are downloaded, and the sequences are clustered at 95% sequence identity. Then, a representative PDB chain that has at least one functional annotation and high-resolution structure is selected into PDBch dataset. The number of sequences in the PDBch dataset differs slightly from the information provided in the DeepFRI. This variation is attributed to the removal of the structure of 5JM5 from the PDB database. Additionally, there are some issues, such as unknown residues for entire chains, in the structures of 6BBM, 6GML, and 6GMH. Therefore, the total number of PDBch set is a little smaller than data from DeepFRI.

To ensure dissimilarity between our training and test sets, we employ the MMseqs (Mirdita *et al.*, 2021) sequence clustering tool with a sequence identity threshold of 30%. What's more, the training, validation, and test sets are then selected from different clusters, with an approximate ratio of 8:1:1, ensuring that the sequence identity between samples from different sets is below 30%. While the sequence identity among different sets is low, the pivotal issue we need to address is the transfer of Gene Ontology (GO) terms from the training set to the test set.

After acquiring the sets, we proceed to assign functional labels to each protein sequence based on the Gene Ontology terms (GO terms) compiled by Gligorijević et al (Gligorijević *et al.*, 2021). These functional labels are categorized into three distinct groups: Molecular Function (MF), Biological Process (BP), and Cellular Components (CC) (Ashburner *et al.*, 2000). Each category serves as an independent prediction task during the training process.

We conduct additional experiment to assess whether recent advancements in protein structure prediction contribute to enhancing domain adaptation. Gligorijević et al (Gligorijević *et al.*, 2021) constructed the SMch dataset through collecting homology protein models of the PDBch dataset from the SWISS-MODEL repository (Waterhouse *et al.*, 2018). These proteins with at least one annotation are kept, and similar sequences are removed at 95% sequence identity. Following Gu et al. (Gu *et al.*, 2023), we select 41,997 proteins from the SMch dataset with low-frequency GO terms (proteins with IC $>$10 from the PDBch dataset), and retrieve their structures predicted by AlphaFold2 (AF2) from the AlphaFold Protein Structure Database (Varadi *et al.*, 2022). This collection forms the AFch dataset. Subsequently, we partition the AFch dataset into training, validation, and test sets, which are then trained alongside the PDBch dataset.To ensure rigorous separation, we establish strict rules for set division: the sequence identity between the training set in AFch and the

test set in PDBch is less than 30%, furthermore, the sequence identity between the test set in AFch and the training set in PDBch is also maintained below 30%. Our objective is to guarantee that the sequence identity of the combined training sets (PDBch and AFch) and the sequence identity of the combined test sets (PDBch and AFch) both remain below the 30% threshold.

Utilizing the frequency of each GO term in the combined training set (PDBch and AFch), we compute the information content values(IC) for GO terms within this set. Higher information content is indicative of more specialized GO terms.

$$IC(GO_i) = -log_2(P(GO_i)). \tag{1}$$

Following this, we evaluate the model's performance on these specialized GO terms—an essential aspect of assessing the overall quality of the model.

## 2 Baseline methods

**Blast** (Altschul *et al.*, 1990). Following Gligorijević et al. (Gligorijević *et al.*, 2021), in case of function transferred from homologous sequences, we remove all sequences similar to the test sequences from the training set using an E-value threshold of 1e-3. Then, we employ the blastp program to identify the sequence with the highest score from both the PDBch and AFch training sets. Subsequently, the predicted annotations of this sequence are adjusted by the sequence identity to the query sequence, yielding the final predicted annotations.

**DeepGOPlus** (Kulmanov and Hoehndorf, 2021). DeepGOPlus is a hybrid method that combines the sequence homology-based DIAMOND Blast with a 1D convolutional neural network, similar to DeepGO. For our evaluation, we retrain DeepGOPlus using the PDBch and AFch training sets, fixing the weight at 0.8 to combine the DIAMOND Blast score with the neural network output. score.

**TALE+** (Cao and Shen, 2021). TALE+ is a protein function annotation method based on the integration of transformers and DIAMOND (Buchfink *et al.*, 2015). This approach involves embedding protein function labels alongside features within a unified latent space. Like the protocol of aforemetioned baseline Blast, sequences in the training set are processed, when DIAMOND blast is utilized for function transferring. We retrain TALE network using the combined training set and subsequently evaluate its performance on our test set.

**DeepFRI** (Gligorijević *et al.*, 2021). DeepFRI is a Graph Convolutional Network designed for predicting protein functions by leveraging sequence features extracted from a protein language model and protein structures. In our evaluation, we retrain the model using our newly established split set to rigorously test our hypothesis.

**Struct2GO** (Ma *et al.*, 2022). Struct2GO is built upon DeepFRI with the aim of testing the hypothesis that incorporating AlphaFold-predicted structures could enhance the accuracy of protein function prediction. The model is retrained using PDBch and AFch training sets, followed by a comprehensive evaluation of its performance.

**HEAL** (Gu *et al.*, 2023). HEAL is an innovative deep learning model that captures structural semantics through a hierarchical graph transformer. It introduces a variety of super-nodes mimicking functional motifs to effectively interact with nodes in the protein graph. In our evaluation, the model undergoes retraining with the combined training set to further enhance its performance.

## 3 AUPR comparison for GO terms on PDBch test set

AUPR is a function-centric measure, calculated as the mean of AUPR for each GO term. To provide more detailed information about each GO term, we calculate the mean, median, 25th percentile, 75th percentile, and coefficient of variation of AUPR for all GO terms, and then compare these statistics with baseline methods. The results are presented in the following three tables, categorized by MF, BP, and CC.

| Methods | Blast | DeepGOPlus | TALE+ | DeeepFRI | Struct2GO | HEAL | GALA-PDB | GALA |
|---|---|---|---|---|---|---|---|---|
| Mean (↑) | 0.126 | 0.136 | 0.158 | 0.321 | 0.523 | 0.516 | 0.539 | 0.555 |
| Median (↑) | 0.070 | 0.057 | 0.100 | 0.268 | 0.537 | 0.558 | 0.622 | 0.621 |
| 25th percentile (↑) | 0.018 | 0.015 | 0.019 | 0.068 | 0.254 | 0.242 | 0.259 | 0.259 |
| 75th percentile (↑) | 0.167 | 0.180 | 0.234 | 0.520 | 0.797 | 0.768 | 0.810 | 0.816 |
| Coefficient of Variation (↓) | 128.464 | 134.440 | 109.654 | 86.531 | 58.238 | 59.610 | 59.120 | 56.839 |

Table S1: AUPR comparison for MF-GO terms on PDBch test set.

| Methods | Blast | DeepGOPlus | TALE+ | DeeepFRI | Struct2GO | HEAL | GALA-PDB | GALA |
|---|---|---|---|---|---|---|---|---|
| Mean (↑) | 0.036 | 0.062 | 0.070 | 0.114 | 0.212 | 0.192 | 0.210 | 0.253 |
| Median (↑) | 0.011 | 0.024 | 0.031 | 0.056 | 0.139 | 0.119 | 0.131 | 0.177 |
| 25th percentile (↑) | 0.002 | 0.008 | 0.009 | 0.016 | 0.031 | 0.038 | 0.040 | 0.051 |
| 75th percentile (↑) | 0.041 | 0.069 | 0.096 | 0.136 | 0.307 | 0.279 | 0.304 | 0.387 |
| Coefficient of Variation (↓) | 178.867 | 163.783 | 137.500 | 145.604 | 107.952 | 107.66 | 106.368 | 96.373 |

Table S2: AUPR comparison for BP-GO terms on PDBch test set.

| Methods | Blast | DeepGOPlus | TALE+ | DeeepFRI | Struct2GO | HEAL | GALA-PDB | GALA |
|---|---|---|---|---|---|---|---|---|
| Mean (↑) | 0.039 | 0.084 | 0.130 | 0.222 | 0.325 | 0.318 | 0.303 | 0.362 |
| Median(↑) | 0.014 | 0.046 | 0.062 | 0.140 | 0.294 | 0.251 | 0.222 | 0.314 |
| 25th percentile(↑) | 0.002 | 0.014 | 0.012 | 0.038 | 0.094 | 0.082 | 0.065 | 0.131 |
| 75th percentile (↑) | 0.047 | 0.104 | 0.189 | 0.327 | 0.486 | 0.487 | 0.468 | 0.545 |
| Coefficient of Variation (↓) | 181.881 | 138.665 | 126.586 | 104.026 | 80.561 | 83.040 | 87.953 | 74.879 |

Table S3: AUPR comparison for CC-GO terms on PDBch test set.

## 4 Performance on PDBch test set under different specificity

Each sequence has been labeled with 489 Molecular Function (MF) terms, 1943 Biological Process (BP) terms, and 320 Cellular Component (CC) terms.

Table S4: Performance of GALA and other baseline methods on PDBch test set under different specificity

| Methods | AUPR | | |
|---|---|---|---|
| | IC<5 | 5<IC<10 | IC>10 |
| Blast | 0.0787 | 0.0579 | 0.0573 |
| DeepGOplus | 0.1805 | 0.0755 | 0.0400 |
| TALE+ | 0.1694 | 0.0970 | 0.0701 |
| DeepFRI | 0.2403 | 0.1659 | 0.1302 |
| Struct2GO | 0.3878 | 0.2819 | 0.2657 |
| HEAL | 0.3854 | 0.2689 | 0.2295 |
| GALA-PDB | 0.4050 | 0.2883 | 0.2276 |
| GALA | 0.4487 | 0.3165 | 0.3074 |

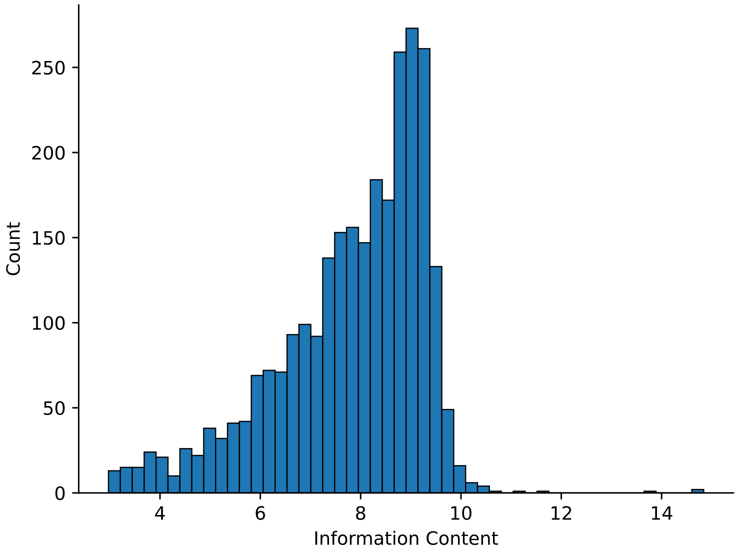


Figure S1: The figure shows the frequency of Information Content(IC) for protein functions over collection of three categories (MF, BP and CC) in the combination of PDBch training set.

## 5 Performance on the AFch test set

Table S5: Performance of GALA and other baseline methods on AFch test set

| Methods | AUPR | | | Fmax | | |
|---|---|---|---|---|---|---|
| | MF | BP | CC | MF | BP | CC |
| Blast | 0.1329 | 0.0463 | 0.0569 | 0.4006 | 0.3564 | 0.3620 |
| DeepGOplus | 0.1827 | 0.0702 | 0.0899 | 0.4212 | 0.4127 | 0.4758 |
| TALE+ | 0.1917 | 0.0716 | 0.0922 | 0.3822 | 0.3768 | 0.4735 |
| DeepFRI | 0.3107 | 0.0964 | 0.1430 | 0.4630 | 0.4175 | 0.4862 |
| Struct2GO | 0.4690 | 0.1640 | 0.2322 | 0.5749 | 0.4885 | 0.5643 |
| HEAL | 0.4813 | 0.1762 | 0.2657 | 0.5900 | 0.5104 | 0.5898 |
| GALA-PDB | 0.4095 | 0.1108 | 0.1397 | 0.5320 | 0.4455 | 0.4880 |
| GALA | 0.4990 | 0.2031 | 0.2766 | 0.6151 | 0.5326 | 0.6121 |

## 6 Runtime for several cases

The runtime for a protein can be divided into two aspects: protein graph construction and model inference. If the protein structure has been experimentally resolved or openly predicted by AlphaFold2, we can use these information as model input. Otherwise, we need to use AlphaFold2 to predict the structure ourselves at the beginning. Additionally, residue embeddings extracted from the protein language model ESM-1b also contribute to protein graph construction. After obtaining the protein structure and residue embeddings of a protein, we can annotate its function using our model GALA. Specifically, we randomly select 5 proteins with a length of approximately 500 residues. The running times for these cases are shown in the following table. If the protein structure is known, then its runtime for molecular function (MF) task consists of the time required for residue embeddings by ESM-1b plus the time for model inference on MF. If the protein structure is unknown, the runtime of the protein for MF task includes the time for structure prediction by AlphaFold2, residue embeddings extracted from ESM-1b, and model inference on MF.

| Protein | Length | Protein Graph Construction(s) | | Model Inference(s) | | |
|---|---|---|---|---|---|---|
| | | Structure Prediction | Residue Embeddings | MF | BP | CC |
| Q8Y210-A | 481 | 1446 | 18.80 | 0.13 | 0.19 | 0.14 |
| Q87VX7-A | 500 | 1632 | 19.18 | 0.14 | 0.20 | 0.22 |
| O49485-A | 544 | 1957 | 19.22 | 0.13 | 0.19 | 0.11 |
| Q09811-A | 536 | 1971 | 17.47 | 0.11 | 0.17 | 0.12 |
| P31411-D | 457 | 1275 | 19.15 | 0.12 | 0.15 | 0.14 |

Table S6: The running times for several cases.

# 7 Plots for interpretability of key redisues

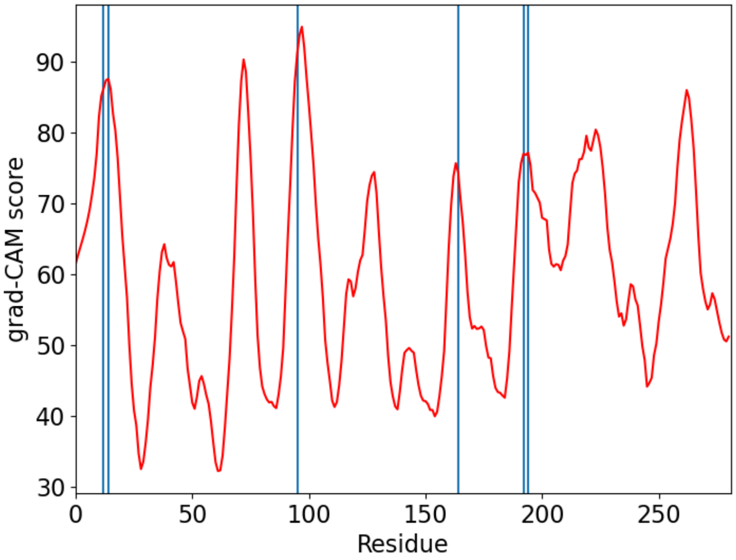


Figure S2: Contribution score computed by Grad-CAM of protein 3DNF with function of iron-sulfur cluster binding (GO:0051536). The red line is the contribution score and the blue lines are the binding sites exacted from the BioLib database. The coincidence of the blue lines and the red line peaks indicates that GALA captures the information of binding sites.

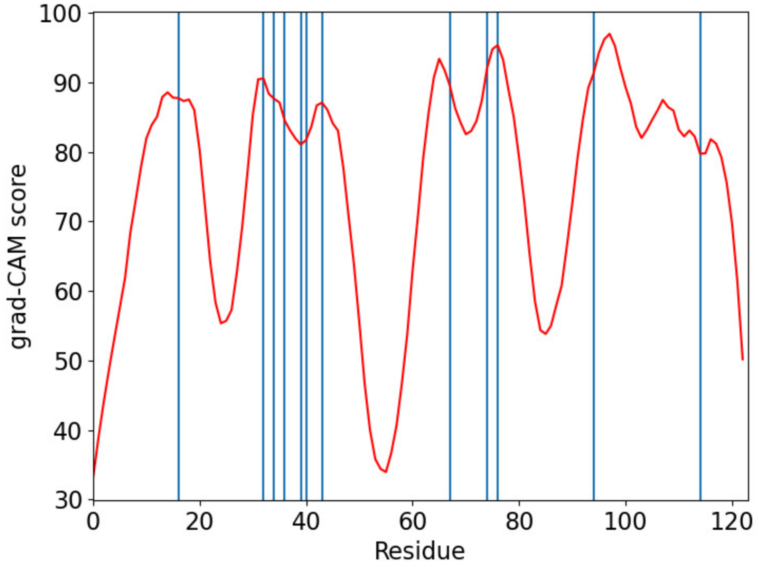


Figure S3: Contribution score computed by Grad-CAM of protein 2ZSC with function of monocarboxylic acid binding (GO:0033293). The red line is the contribution score and the blue lines denote the binding sites exacted from the BioLib database. The coincidence of the blue lines and the red line peaks indicates that GALA captures the information of binding sites.

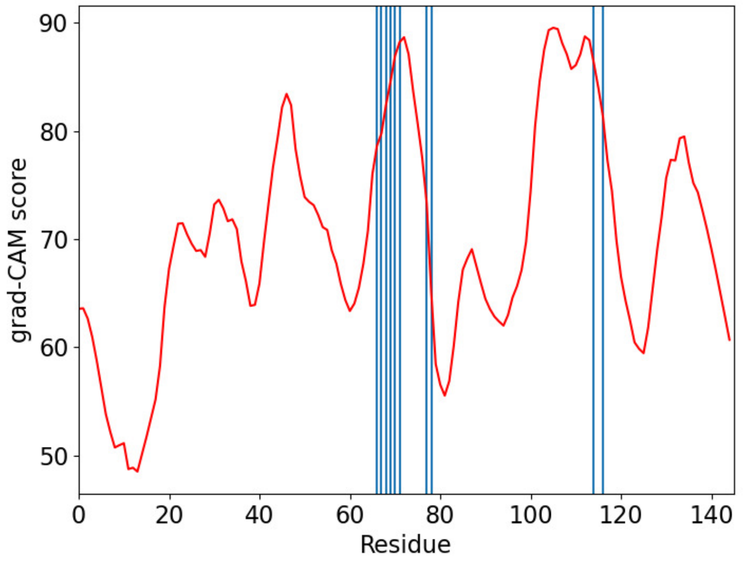


Figure S4: Contribution score computed by Grad-CAM of protein 1P4U with function of peptide transport (GO:0015833). The red line is the contribution score and the blue lines denote the binding sites exacted from the BioLib database. The coincidence of the blue lines and the red line peaks indicates that GALA captures the information of binding sites.

## References


Altschul, S. F. *et al.* (1990). Basic local alignment search tool. *Journal of Molecular Biology*, **215**(3), 403–410.

Ashburner, M. *et al.* (2000). Gene ontology: tool for the unification of biology. *Nature Genetics*, **25**(1), 25–29.

Berman, H. M. *et al.* (2000). The protein data bank. *Nucleic Acids Research*, **28**(1), 235–242.

Buchfink, B. *et al.* (2015). Fast and sensitive protein alignment using diamond. *Nature methods*, **12**(1), 59–60.

Cao, Y. and Shen, Y. (2021). Tale: Transformer-based protein function annotation with joint sequence–label embedding. *Bioinformatics*, **37**(18), 2825–2833.

Dana, J. M. *et al.* (2019). Sifts: updated structure integration with function, taxonomy and sequences resource allows 40-fold increase in coverage of structure-based annotations for proteins. *Nucleic Acids Research*, **47**(D1), D482–D489.

Gligorijević, V. *et al.* (2021). Structure-based protein function prediction using graph convolutional networks. *Nature Communications*, **12**(1), 3168.

Gu, Z. *et al.* (2023). Hierarchical graph transformer with contrastive learning for protein function prediction. *Bioinformatics*, **39**(7), btad410.

Kulmanov, M. and Hoehndorf, R. (2021). Deepgoplus: improved protein function prediction from sequence. *Bioinformatics*, **37**(8), 1187.

Ma, W. *et al.* (2022). Enhancing protein function prediction performance by utilizing alphafold-predicted protein structures. *Journal of Chemical Information and Modeling*, **62**(17), 4008–4017.

Mirdita, M. *et al.* (2021). Fast and sensitive taxonomic assignment to metagenomic contigs. *Bioinformatics*, **37**(18), 3029–3031.

Varadi, M. *et al.* (2022). Alphafold protein structure database: massively expanding the structural coverage of protein-sequence space with high-accuracy models. *Nucleic Acids Research*, **50**(D1), D439–D444.

Waterhouse, A. *et al.* (2018). Swiss-model: homology modelling of protein structures and complexes. *Nucleic Acids Research*, **46**(W1), W296–W303.
