# Supplementary material for: Learning a generalized graph transformer for protein function prediction in dissimilar sequences
Source: Gigascience. 2024 Dec 5;13:giae093. doi: 10.1093/gigascience/giae093 (PMC11734293; doi:10.1093/gigascience/giae093)
Supplement: giae093_GIGA-D-24-00109_Revision_3 [file giae093_giga-d-24-00109_revision_3.pdf]

GigaScience

Learning A Generalized Graph Transformer for Protein Function Prediction in Dissimilar Sequences

--Manuscript Draft--

**Manuscript Number:**	GIGA-D-24-00109R3
**Full Title:**	Learning A Generalized Graph Transformer for Protein Function Prediction in Dissimilar Sequences
**Article Type:**	Technical Note
**Funding Information:**	National Natural Science Foundation of China (32270689) — Professor Minghua Deng<br>Key Technologies Research and Development Program (2021YFF1200902) — Professor Minghua Deng
**Abstract:**	(see below)

Funding Information:

Funder	Recipient
National Natural Science Foundation of China (32270689)	Professor Minghua Deng
Key Technologies Research and Development Program (2021YFF1200902)	Professor Minghua Deng

Abstract:

Background

In the face of a growing disparity between high-throughput sequence data and low-throughput experimental studies, the emerging field of deep learning stands as a promising alternative. Generally, many data-driven approaches are capable of facilitating fast and accurate predictions of protein functions. Nevertheless, the inherent statistical nature of deep learning techniques may limit their generalization capabilities when applied to novel non-homologous proteins that diverge significantly from existing ones.

Results

In this work, we propose a novel, generalized approach named Graph Adversarial Learning with Alignment (GALA) for protein function prediction. Our GALA model integrates a graph transformer architecture with an attention pooling module to extract information from both protein sequences and structures, facilitating unified learning of protein structural representations. Particularly noteworthy, GALA incorporates a domain discriminator conditioned on both representations and predicted probabilities, which undergoes adversarial training to ensure representation invariance across diverse environments. To optimize the model with abundant label information, we generate label embeddings in the hidden space, explicitly aligning them with protein representations. Benchmarked on datasets derived from PDB database and Swiss-Prot database,our GALA achieves performance comparable to several state-of-the-art methods. Furthermore, GALA demonstrates outstanding interpretability by identifying key functional residues associated with GO terms through class activation mapping.

Conclusions

GALA, which leverages adversarial learning and label embedding alignment to acquire domain-invariant protein representations, exhibits outstanding generalizability in function prediction for proteins from previously unseen sequence space. By utilizing the structures predicted by AlphaFold2, GALA holds significant potential for function annotation in newly discovered sequences. Implementations of our GALA can be found at https://github.com/fuyw-aisw/GALA.

**Corresponding Author:**	yiwei Fu<br>Peking University<br>Beijing, --- Select One --- CHINA
**Corresponding Author Secondary Information:**	
**Corresponding Author's Institution:**	Peking University
**Corresponding Author's Secondary Institution:**	

Powered by Editorial Manager® and ProduXion Manager® from Aries Systems Corporation

**First Author:**	Yiwei Fu
**First Author Secondary Information:**	
**Order of Authors:**	Yiwei Fu
	Zhonghui Gu
	Xiao Luo
	Qirui Guo
	Luhua Lai
	Minghua Deng
**Order of Authors Secondary Information:**	
**Response to Reviewers:**	Hans Zauner, Ph.D.<br>GigaScience<br>Dear Editor:<br><br>Thanks for forwarding the valuable comments on our manuscript "Learning A Generalized Graph Transformer for Protein Function Prediction in Dissimilar Sequences" (GIGA-D-24-00109_R3). We have revised our manuscript accordingly.<br><br>1. The GigaDB dataset has been cited in the "Data Availability" section.<br><br>2. DOME-ML annotations have been mentioned accordingly.<br><br>3. The URLs for data sources have been correctly revised in the text.<br><br>4. ORCIDs have been added to the title page.<br><br>5. All tracked changes marked in red have been removed from the manuscript.<br><br>6.The LaTeX source files have been attached.<br><br>Additionally, the RRID from SciCrunch.org databases has been included in the "Availability of Supporting Source Code and Requirements" section, with the RRID being "SCR_025194".<br><br>We believe our work presents significant technical advances in protein analysis, and hope the paper is now acceptable for publication in GigaScience. Once again, we greatly appreciate your effort and time spent revising our manuscript.<br><br>With Best Regards,<br>Dr. Minghua Deng<br>Peking University
**Additional Information:**	
**Question**	**Response**
Are you submitting this manuscript to a special series or article collection?	No
**Experimental design and statistics**<br><br>Full details of the experimental design and statistical methods used should be given in the Methods section, as detailed in our Minimum Standards Reporting Checklist. Information essential to interpreting the data presented should be made available	Yes

in the figure legends.<br><br>Have you included all the information requested in your manuscript?	
**Resources**<br><br>A description of all resources used, including antibodies, cell lines, animals and software tools, with enough information to allow them to be uniquely identified, should be included in the Methods section. Authors are strongly encouraged to cite Research Resource Identifiers (RRIDs) for antibodies, model organisms and tools, where possible.<br><br>Have you included the information requested as detailed in our Minimum Standards Reporting Checklist?	Yes
**Availability of data and materials**<br><br>All datasets and code on which the conclusions of the paper rely must be either included in your submission or deposited in publicly available repositories (where available and ethically appropriate), referencing such data using a unique identifier in the references and in the "Availability of Data and Materials" section of your manuscript.<br><br>Have you have met the above requirement as detailed in our Minimum Standards Reporting Checklist?	Yes

Click here to access/download;LaTeX - Main Document (TeX file);main.tex

```
This is pdfTeX, Version 3.141592653-2.6-1.40.26 (TeX Live 2024)
(preloaded format=pdflatex 2024.8.2)  18 OCT 2024 09:18
entering extended mode
 restricted \write18 enabled.
 %&-line parsing enabled.
**main.tex
(./main.tex
LaTeX2e <2024-06-01> patch level 2
L3 programming layer <2024-05-27>
(./oup-contemporary.cls
Document Class: oup-contemporary 2023/06/12, v1.2
(c:/texlive/2024/texmf-dist/tex/latex/base/article.cls
Document Class: article 2024/02/08 v1.4n Standard LaTeX document class
(c:/texlive/2024/texmf-dist/tex/latex/base/size10.clo
File: size10.clo 2024/02/08 v1.4n Standard LaTeX file (size option)
)
\c@part=\count194
\c@section=\count195
\c@subsection=\count196
\c@subsubsection=\count197
\c@paragraph=\count198
\c@subparagraph=\count199
\c@figure=\count266
\c@table=\count267
\abovecaptionskip=\skip49
\belowcaptionskip=\skip50
\bibindent=\dimen141
) (c:/texlive/2024/texmf-dist/tex/latex/base/inputenc.sty
Package: inputenc 2024/02/08 v1.3d Input encoding file
\inpenc@prehook=\toks17
\inpenc@posthook=\toks18
) (c:/texlive/2024/texmf-dist/tex/latex/base/fontenc.sty
Package: fontenc 2021/04/29 v2.0v Standard LaTeX package
) (c:/texlive/2024/texmf-dist/tex/generic/iftex/ifpdf.sty
Package: ifpdf 2019/10/25 v3.4 ifpdf legacy package. Use iftex instead.
(c:/texlive/2024/texmf-dist/tex/generic/iftex/iftex.sty
Package: iftex 2022/02/03 v1.0f TeX engine tests
)) (c:/texlive/2024/texmf-dist/tex/latex/microtype/microtype.sty
Package: microtype 2024/03/29 v3.1b Micro-typographical refinements (RS)
(c:/texlive/2024/texmf-dist/tex/latex/graphics/keyval.sty
Package: keyval 2022/05/29 v1.15 key=value parser (DPC)
\KV@toks@=\toks19
) (c:/texlive/2024/texmf-dist/tex/latex/etoolbox/etoolbox.sty
Package: etoolbox 2020/10/05 v2.5k e-TeX tools for LaTeX (JAW)
\etb@tempcnta=\count268
)
\MT@toks=\toks20
\MT@tempbox=\box52
\MT@count=\count269
LaTeX Info: Redefining \noprotrusionifhmode on input line 1061.
LaTeX Info: Redefining \leftprotrusion on input line 1062.
\MT@prot@toks=\toks21
LaTeX Info: Redefining \rightprotrusion on input line 1081.
LaTeX Info: Redefining \textls on input line 1392.
```

```
\MT@outer@kern=\dimen142
LaTeX Info: Redefining \textmicrotypecontext on input line 2013.
\MT@listname@count=\count270
(c:/texlive/2024/texmf-dist/tex/latex/microtype/microtype-pdftex.def
File: microtype-pdftex.def 2024/03/29 v3.1b Definitions specific to
pdftex (RS)

LaTeX Info: Redefining \lsstyle on input line 902.
LaTeX Info: Redefining \lslig on input line 902.
\MT@outer@space=\skip51
)
Package microtype Info: Loading configuration file microtype.cfg.
(c:/texlive/2024/texmf-dist/tex/latex/microtype/microtype.cfg
File: microtype.cfg 2024/03/29 v3.1b microtype main configuration file
(RS)
)) (c:/texlive/2024/texmf-dist/tex/latex/euler/euler.sty
Package: euler 1995/03/05 v2.5
Package: `euler' v2.5 <1995/03/05> (FJ and FMi)
LaTeX Font Info:    Redeclaring symbol font `letters' on input line 35.
LaTeX Font Info:    Encoding `OML' has changed to `U' for symbol font
(Font)              `letters' in the math version `normal' on input line
35.
LaTeX Font Info:    Overwriting symbol font `letters' in version `normal'
(Font)                  OML/cmm/m/it --> U/eur/m/n on input line 35.
LaTeX Font Info:    Encoding `OML' has changed to `U' for symbol font
(Font)              `letters' in the math version `bold' on input line
35.
LaTeX Font Info:    Overwriting symbol font `letters' in version `bold'
(Font)                  OML/cmm/b/it --> U/eur/m/n on input line 35.
LaTeX Font Info:    Overwriting symbol font `letters' in version `bold'
(Font)                  U/eur/m/n --> U/eur/b/n on input line 36.
LaTeX Font Info:    Redeclaring math symbol \Gamma on input line 47.
LaTeX Font Info:    Redeclaring math symbol \Delta on input line 48.
LaTeX Font Info:    Redeclaring math symbol \Theta on input line 49.
LaTeX Font Info:    Redeclaring math symbol \Lambda on input line 50.
LaTeX Font Info:    Redeclaring math symbol \Xi on input line 51.
LaTeX Font Info:    Redeclaring math symbol \Pi on input line 52.
LaTeX Font Info:    Redeclaring math symbol \Sigma on input line 53.
LaTeX Font Info:    Redeclaring math symbol \Upsilon on input line 54.
LaTeX Font Info:    Redeclaring math symbol \Phi on input line 55.
LaTeX Font Info:    Redeclaring math symbol \Psi on input line 56.
LaTeX Font Info:    Redeclaring math symbol \Omega on input line 57.
\symEulerFraktur=\mathgroup4
LaTeX Font Info:    Overwriting symbol font `EulerFraktur' in version
`bold'
(Font)                  U/euf/m/n --> U/euf/b/n on input line 63.
LaTeX Info: Redefining \oldstylenums on input line 85.
\symEulerScript=\mathgroup5
LaTeX Font Info:    Overwriting symbol font `EulerScript' in version
`bold'
(Font)                  U/eus/m/n --> U/eus/b/n on input line 93.
LaTeX Font Info:    Redeclaring math symbol \aleph on input line 97.
LaTeX Font Info:    Redeclaring math symbol \Re on input line 98.
LaTeX Font Info:    Redeclaring math symbol \Im on input line 99.
```

```
LaTeX Font Info:    Redeclaring math delimiter \vert on input line 101.
LaTeX Font Info:    Redeclaring math delimiter \backslash on input line
103.
LaTeX Font Info:    Redeclaring math symbol \neg on input line 106.
LaTeX Font Info:    Redeclaring math symbol \wedge on input line 108.
LaTeX Font Info:    Redeclaring math symbol \vee on input line 110.
LaTeX Font Info:    Redeclaring math symbol \setminus on input line 112.
LaTeX Font Info:    Redeclaring math symbol \sim on input line 113.
LaTeX Font Info:    Redeclaring math symbol \mid on input line 114.
LaTeX Font Info:    Redeclaring math delimiter \arrowvert on input line
116.
LaTeX Font Info:    Redeclaring math symbol \mathsection on input line
117.
\symEulerExtension=\mathgroup6
LaTeX Font Info:    Redeclaring math symbol \coprod on input line 125.
LaTeX Font Info:    Redeclaring math symbol \prod on input line 125.
LaTeX Font Info:    Redeclaring math symbol \sum on input line 125.
LaTeX Font Info:    Redeclaring math symbol \intop on input line 130.
LaTeX Font Info:    Redeclaring math symbol \ointop on input line 131.
LaTeX Font Info:    Redeclaring math symbol \braceld on input line 132.
LaTeX Font Info:    Redeclaring math symbol \bracerd on input line 133.
LaTeX Font Info:    Redeclaring math symbol \bracelu on input line 134.
LaTeX Font Info:    Redeclaring math symbol \braceru on input line 135.
LaTeX Font Info:    Redeclaring math symbol \infty on input line 136.
LaTeX Font Info:    Redeclaring math symbol \nearrow on input line 153.
LaTeX Font Info:    Redeclaring math symbol \searrow on input line 154.
LaTeX Font Info:    Redeclaring math symbol \nwarrow on input line 155.
LaTeX Font Info:    Redeclaring math symbol \swarrow on input line 156.
LaTeX Font Info:    Redeclaring math symbol \Leftrightarrow on input line
157.
LaTeX Font Info:    Redeclaring math symbol \Leftarrow on input line 158.
LaTeX Font Info:    Redeclaring math symbol \Rightarrow on input line
159.
LaTeX Font Info:    Redeclaring math symbol \leftrightarrow on input line
160.
LaTeX Font Info:    Redeclaring math symbol \leftarrow on input line 161.
LaTeX Font Info:    Redeclaring math symbol \rightarrow on input line
163.
LaTeX Font Info:    Redeclaring math delimiter \uparrow on input line
166.
LaTeX Font Info:    Redeclaring math delimiter \downarrow on input line
168.
LaTeX Font Info:    Redeclaring math delimiter \updownarrow on input line
170.
LaTeX Font Info:    Redeclaring math delimiter \Uparrow on input line
172.
LaTeX Font Info:    Redeclaring math delimiter \Downarrow on input line
174.
LaTeX Font Info:    Redeclaring math delimiter \Updownarrow on input line
176.
LaTeX Font Info:    Redeclaring math symbol \leftharpoonup on input line
177.
LaTeX Font Info:    Redeclaring math symbol \leftharpoondown on input
line 178.
```

```
LaTeX Font Info:    Redeclaring math symbol \rightharpoonup on input line
179.
LaTeX Font Info:    Redeclaring math symbol \rightharpoondown on input
line 180
.
LaTeX Font Info:    Redeclaring math delimiter \lbrace on input line 182.
LaTeX Font Info:    Redeclaring math delimiter \rbrace on input line 184.
\symcmmigroup=\mathgroup7
LaTeX Font Info:    Overwriting symbol font `cmmigroup' in version `bold'
(Font)                  OML/cmm/m/it --> OML/cmm/b/it on input line 200.
LaTeX Font Info:    Redeclaring math accent \vec on input line 201.
LaTeX Font Info:    Redeclaring math symbol \triangleleft on input line
202.
LaTeX Font Info:    Redeclaring math symbol \triangleright on input line
203.
LaTeX Font Info:    Redeclaring math symbol \star on input line 204.
LaTeX Font Info:    Redeclaring math symbol \lhook on input line 205.
LaTeX Font Info:    Redeclaring math symbol \rhook on input line 206.
LaTeX Font Info:    Redeclaring math symbol \flat on input line 207.
LaTeX Font Info:    Redeclaring math symbol \natural on input line 208.
LaTeX Font Info:    Redeclaring math symbol \sharp on input line 209.
LaTeX Font Info:    Redeclaring math symbol \smile on input line 210.
LaTeX Font Info:    Redeclaring math symbol \frown on input line 211.
LaTeX Font Info:    Redeclaring math accent \grave on input line 245.
LaTeX Font Info:    Redeclaring math accent \acute on input line 246.
LaTeX Font Info:    Redeclaring math accent \tilde on input line 247.
LaTeX Font Info:    Redeclaring math accent \ddot on input line 248.
LaTeX Font Info:    Redeclaring math accent \check on input line 249.
LaTeX Font Info:    Redeclaring math accent \breve on input line 250.
LaTeX Font Info:    Redeclaring math accent \bar on input line 251.
LaTeX Font Info:    Redeclaring math accent \dot on input line 252.
LaTeX Font Info:    Redeclaring math accent \hat on input line 254.
) (c:/texlive/2024/texmf-dist/tex/latex/merriweather/merriweather.sty
Package: merriweather 2022/09/20 (Bob Tennent) Supports
Merriweather(Sans) font
s for all LaTeX engines.
(c:/texlive/2024/texmf-dist/tex/generic/iftex/ifxetex.sty
Package: ifxetex 2019/10/25 v0.7 ifxetex legacy package. Use iftex
instead.
) (c:/texlive/2024/texmf-dist/tex/generic/iftex/ifluatex.sty
Package: ifluatex 2019/10/25 v1.5 ifluatex legacy package. Use iftex
instead.
) (c:/texlive/2024/texmf-dist/tex/latex/base/textcomp.sty
Package: textcomp 2024/04/24 v2.1b Standard LaTeX package
) (c:/texlive/2024/texmf-dist/tex/latex/xkeyval/xkeyval.sty
Package: xkeyval 2022/06/16 v2.9 package option processing (HA)
(c:/texlive/2024/texmf-dist/tex/generic/xkeyval/xkeyval.tex
(c:/texlive/2024/te
xmf-dist/tex/generic/xkeyval/xkvutils.tex
\XKV@toks=\toks22
\XKV@tempa@toks=\toks23
)
\XKV@depth=\count271
```

```
File: xkeyval.tex 2014/12/03 v2.7a key=value parser (HA)
)) (c:/texlive/2024/texmf-dist/tex/latex/base/fontenc.sty
Package: fontenc 2021/04/29 v2.0v Standard LaTeX package
) (c:/texlive/2024/texmf-dist/tex/latex/fontaxes/fontaxes.sty
Package: fontaxes 2020/07/21 v1.0e Font selection axes
LaTeX Info: Redefining \upshape on input line 29.
LaTeX Info: Redefining \itshape on input line 31.
LaTeX Info: Redefining \slshape on input line 33.
LaTeX Info: Redefining \swshape on input line 35.
LaTeX Info: Redefining \scshape on input line 37.
LaTeX Info: Redefining \sscshape on input line 39.
LaTeX Info: Redefining \ulcshape on input line 41.
LaTeX Info: Redefining \textsw on input line 47.
LaTeX Info: Redefining \textssc on input line 48.
LaTeX Info: Redefining \textulc on input line 49.
)) (c:/texlive/2024/texmf-dist/tex/latex/mathastext/mathastext.sty
Package: mathastext 2024/07/27 v1.4b Use the text font in math mode (JFB)

Package mathastext Info: Starting the math mode configuration.
\mst@exists@muskip=\muskip17
\mst@forall@muskip=\muskip18
\mst@prime@muskip=\muskip19
\mst@do@nonletters=\toks24
\mst@undo@nonletters=\toks25
\mst@do@easynonletters=\toks26
\mst@undo@easynonletters=\toks27
\symmtoperatorfont=\mathgroup8
\symmtletterfont=\mathgroup9
(   mathastext:   ) ! and ?
(   mathastext:   ) punctuation: , . : ; and \colon
LaTeX Info: Redefining \relbar on input line 1201.
LaTeX Info: Redefining \rightarrowfill on input line 1202.
LaTeX Info: Redefining \leftarrowfill on input line 1205.
(   mathastext:   ) + and =
LaTeX Info: Redefining \Relbar on input line 1298.
(   mathastext:   ) adding = ; and + to \nfss@catcodes
(   mathastext:   ) parentheses ( ) [ ] and slash /
(   mathastext:   ) alldelims: < > \backslash \setminus | \vert \mid \{
\}
LaTeX Font Info:    Redeclaring math symbol \setminus on input line 1364.
LaTeX Info: Redefining \models on input line 1383.
(   mathastext:   ) \# \mathdollar \% \&
(   mathastext:   ) \imath and \jmath
LaTeX Font Info:    Overwriting math alphabet `\Mathnormalbold' in
version `nor
mal'
(Font)                  T1/Merriwthr-OsF/b/it --> T1/Merriwthr-OsF/b/it
on inpu
t line 2863.
LaTeX Font Info:    Overwriting math alphabet `\Mathnormalbold' in
version `bol
d'
(Font)                  T1/Merriwthr-OsF/b/it --> T1/Merriwthr-OsF/b/it
on inpu
```

```
t line 2863.
LaTeX Font Info:    Overwriting symbol font `mtletterfont' in version
`normal'
(Font)                  T1/Merriwthr-OsF/m/it --> T1/Merriwthr-OsF/m/it
on inpu
t line 2863.
LaTeX Font Info:    Overwriting symbol font `mtletterfont' in version
`bold'
(Font)                  T1/Merriwthr-OsF/m/it --> T1/Merriwthr-OsF/b/it
on inpu
t line 2863.
LaTeX Font Info:    Overwriting symbol font `mtoperatorfont' in version
`normal
'
(Font)                  T1/Merriwthr-OsF/m/n --> T1/Merriwthr-OsF/m/n on
input
line 2863.
LaTeX Font Info:    Overwriting symbol font `mtoperatorfont' in version
`bold'
(Font)                  T1/Merriwthr-OsF/m/n --> T1/Merriwthr-OsF/b/n on
input
line 2863.
LaTeX Font Info:    Overwriting math alphabet `\Mathbf' in version
`normal'
(Font)                  T1/Merriwthr-OsF/b/n --> T1/Merriwthr-OsF/b/n on
input
line 2863.
LaTeX Font Info:    Overwriting math alphabet `\Mathbf' in version `bold'
(Font)                  T1/Merriwthr-OsF/b/n --> T1/Merriwthr-OsF/b/n on
input
line 2863.
LaTeX Font Info:    Overwriting math alphabet `\Mathit' in version
`normal'
(Font)                  T1/Merriwthr-OsF/m/it --> T1/Merriwthr-OsF/m/it
on inpu
t line 2863.
LaTeX Font Info:    Overwriting math alphabet `\Mathit' in version `bold'
(Font)                  T1/Merriwthr-OsF/m/it --> T1/Merriwthr-OsF/b/it
on inpu
t line 2863.
LaTeX Font Info:    Overwriting math alphabet `\Mathsf' in version
`normal'
(Font)                  T1/MerriwthrSans-OsF/m/n --> T1/MerriwthrSans-
OsF/m/n o
n input line 2863.
LaTeX Font Info:    Overwriting math alphabet `\Mathsf' in version `bold'
(Font)                  T1/MerriwthrSans-OsF/m/n --> T1/MerriwthrSans-
OsF/b/n o
n input line 2863.
LaTeX Font Info:    Overwriting math alphabet `\Mathtt' in version
`normal'
(Font)                  T1/lmtt/m/n --> T1/lmtt/m/n on input line 2863.
LaTeX Font Info:    Overwriting math alphabet `\Mathtt' in version `bold'
(Font)                  T1/lmtt/m/n --> T1/lmtt/b/n on input line 2863.
```

```
(   mathastext:   ) Latin letters in the `normal', resp. `bold',
(   mathastext:   ) math versions are now set up to use the fonts
(   mathastext:   ) T1/Merriwthr-OsF/m/it, resp. T1/Merriwthr-OsF/b/it.
(   mathastext:   ) Other characters (digits, ...) and \log-like names
will be
(   mathastext:   ) typeset with the n shape.
(   mathastext:   ) \hbar
(   mathastext:   ) minus as endash
(   mathastext:   ) The italic option is in effect.
(   mathastext:   ) \HUGE has been (re)-defined.
(   mathastext:   ) mathastext has declared larger sizes for subscripts.
(   mathastext:   ) To keep LaTeX defaults, use option
`defaultmathsizes'.

Package mathastext Info: Loading is complete.  You can now use
\Mathastext to
(mathastext)             modify the normal and bold math versions.  Use
it
(mathastext)             with optional argument or use \MTDeclareVersion
to
(mathastext)             declare additional math versions.
) (c:/texlive/2024/texmf-dist/tex/latex/relsize/relsize.sty
Package: relsize 2013/03/29 ver 4.1
) (c:/texlive/2024/texmf-dist/tex/latex/ragged2e/ragged2e.sty
Package: ragged2e 2023/06/22 v3.6 ragged2e Package
\CenteringLeftskip=\skip52
\RaggedLeftLeftskip=\skip53
\RaggedRightLeftskip=\skip54
\CenteringRightskip=\skip55
\RaggedLeftRightskip=\skip56
\RaggedRightRightskip=\skip57
\CenteringParfillskip=\skip58
\RaggedLeftParfillskip=\skip59
\RaggedRightParfillskip=\skip60
\JustifyingParfillskip=\skip61
\CenteringParindent=\skip62
\RaggedLeftParindent=\skip63
\RaggedRightParindent=\skip64
\JustifyingParindent=\skip65
) (c:/texlive/2024/texmf-dist/tex/latex/xcolor/xcolor.sty
Package: xcolor 2023/11/15 v3.01 LaTeX color extensions (UK)
(c:/texlive/2024/texmf-dist/tex/latex/graphics-cfg/color.cfg
File: color.cfg 2016/01/02 v1.6 sample color configuration
)
Package xcolor Info: Driver file: pdftex.def on input line 274.
(c:/texlive/2024/texmf-dist/tex/latex/graphics-def/pdftex.def
File: pdftex.def 2024/04/13 v1.2c Graphics/color driver for pdftex
) (c:/texlive/2024/texmf-dist/tex/latex/graphics/mathcolor.ltx)
Package xcolor Info: Model `cmy' substituted by `cmy0' on input line
1350.
Package xcolor Info: Model `hsb' substituted by `rgb' on input line 1354.
Package xcolor Info: Model `RGB' extended on input line 1366.
Package xcolor Info: Model `HTML' substituted by `rgb' on input line
1368.
```

```
Package xcolor Info: Model `Hsb' substituted by `hsb' on input line 1369.
Package xcolor Info: Model `tHsb' substituted by `hsb' on input line
1370.
Package xcolor Info: Model `HSB' substituted by `hsb' on input line 1371.
Package xcolor Info: Model `Gray' substituted by `gray' on input line
1372.
Package xcolor Info: Model `wave' substituted by `hsb' on input line
1373.
) (c:/texlive/2024/texmf-dist/tex/latex/colortbl/colortbl.sty
Package: colortbl 2024/07/06 v1.0i Color table columns (DPC)
(c:/texlive/2024/texmf-dist/tex/latex/tools/array.sty
Package: array 2024/06/14 v2.6d Tabular extension package (FMi)
\col@sep=\dimen143
\ar@mcellbox=\box53
\extrarowheight=\dimen144
\NC@list=\toks28
\extratabsurround=\skip66
\backup@length=\skip67
\ar@cellbox=\box54
)
\everycr=\toks29
\minrowclearance=\skip68
\rownum=\count272
) (c:/texlive/2024/texmf-dist/tex/latex/graphics/graphicx.sty
Package: graphicx 2021/09/16 v1.2d Enhanced LaTeX Graphics (DPC,SPQR)
(c:/texlive/2024/texmf-dist/tex/latex/graphics/graphics.sty
Package: graphics 2024/05/23 v1.4g Standard LaTeX Graphics (DPC,SPQR)
(c:/texlive/2024/texmf-dist/tex/latex/graphics/trig.sty
Package: trig 2023/12/02 v1.11 sin cos tan (DPC)
) (c:/texlive/2024/texmf-dist/tex/latex/graphics-cfg/graphics.cfg
File: graphics.cfg 2016/06/04 v1.11 sample graphics configuration
)
Package graphics Info: Driver file: pdftex.def on input line 106.
)
\Gin@req@height=\dimen145
\Gin@req@width=\dimen146
) (c:/texlive/2024/texmf-dist/tex/latex/xpatch/xpatch.sty
(c:/texlive/2024/texm
f-dist/tex/latex/l3kernel/expl3.sty
Package: expl3 2024-05-27 L3 programming layer (loader)
(c:/texlive/2024/texmf-dist/tex/latex/l3backend/l3backend-pdftex.def
File: l3backend-pdftex.def 2024-05-08 L3 backend support: PDF output
(pdfTeX)
\l__color_backend_stack_int=\count273
\l__pdf_internal_box=\box55
))
Package: xpatch 2020/03/25 v0.3a Extending etoolbox patching commands
(c:/texlive/2024/texmf-dist/tex/latex/l3packages/xparse/xparse.sty
Package: xparse 2024-05-08 L3 Experimental document command parser
)) (c:/texlive/2024/texmf-dist/tex/latex/environ/environ.sty
Package: environ 2014/05/04 v0.3 A new way to define environments
(c:/texlive/2024/texmf-dist/tex/latex/trimspaces/trimspaces.sty
Package: trimspaces 2009/09/17 v1.1 Trim spaces around a token list
)
```

```
\@envbody=\toks30
) (c:/texlive/2024/texmf-dist/tex/latex/lastpage/lastpage.sty
Package: lastpage 2024/07/07 v2.1c lastpage: 2.09 or 2e? (HMM)
(c:/texlive/2024/texmf-dist/tex/latex/lastpage/lastpage2e.sty
Package: lastpage2e 2024/07/07 v2.1c Decide which 2e lastpage version to
use (H
MM)
(c:/texlive/2024/texmf-dist/tex/latex/lastpage/lastpagemodern.sty
Package: lastpagemodern 2024-07-07 v2.1c Refers to last page's name (HMM;
JPG)
\c@lastpagecount=\count274
)
)) (c:/texlive/2024/texmf-dist/tex/latex/graphics/rotating.sty
Package: rotating 2016/08/11 v2.16d rotated objects in LaTeX
(c:/texlive/2024/texmf-dist/tex/latex/base/ifthen.sty
Package: ifthen 2024/03/16 v1.1e Standard LaTeX ifthen package (DPC)
)
\c@r@tfl@t=\count275
\rotFPtop=\skip69
\rotFPbot=\skip70
\rot@float@box=\box56
\rot@mess@toks=\toks31
) (c:/texlive/2024/texmf-dist/tex/latex/graphics/lscape.sty
Package: lscape 2020/05/28 v3.02 Landscape Pages (DPC)
) (c:/texlive/2024/texmf-dist/tex/latex/tools/afterpage.sty
Package: afterpage 2023/07/04 v1.08 After-Page Package (DPC)
\AP@output=\toks32
\AP@partial=\box57
\AP@footins=\box58
) (c:/texlive/2024/texmf-dist/tex/latex/textpos/textpos.sty
Package: textpos 2022/07/23 v1.10.1
Package textpos Info: choosing support for LaTeX3 on input line 60.
\TP@textbox=\box59
\TP@holdbox=\box60
\TPHorizModule=\dimen147
\TPVertModule=\dimen148
\TP@margin=\dimen149
\TP@absmargin=\dimen150
Grid set 16 x 16 = 37.34424pt x 52.81541pt
\TPboxrulesize=\dimen151
\TP@ox=\dimen152
\TP@oy=\dimen153
\TP@tbargs=\toks33
TextBlockOrigin set to 0pt x 0pt
) (c:/texlive/2024/texmf-dist/tex/latex/url/url.sty
\Urlmuskip=\muskip20
Package: url 2013/09/16  ver 3.4  Verb mode for urls, etc.
) (c:/texlive/2024/texmf-dist/tex/latex/newfloat/newfloat.sty
Package: newfloat 2023/10/01 v1.2 Defining new floating environments (AR)
Package newfloat Info: `rotating' package detected.
) (c:/texlive/2024/texmf-dist/tex/latex/mdframed/mdframed.sty
Package: mdframed 2013/07/01 1.9b: mdframed
(c:/texlive/2024/texmf-dist/tex/latex/kvoptions/kvoptions.sty
```

```
Package: kvoptions 2022-06-15 v3.15 Key value format for package options
(HO)
(c:/texlive/2024/texmf-dist/tex/generic/ltxcmds/ltxcmds.sty
Package: ltxcmds 2023-12-04 v1.26 LaTeX kernel commands for general use
(HO)
) (c:/texlive/2024/texmf-dist/tex/latex/kvsetkeys/kvsetkeys.sty
Package: kvsetkeys 2022-10-05 v1.19 Key value parser (HO)
)) (c:/texlive/2024/texmf-dist/tex/latex/zref/zref-abspage.sty
Package: zref-abspage 2023-09-14 v2.35 Module abspage for zref (HO)
(c:/texlive/2024/texmf-dist/tex/latex/zref/zref-base.sty
Package: zref-base 2023-09-14 v2.35 Module base for zref (HO)
(c:/texlive/2024/texmf-dist/tex/generic/infwarerr/infwarerr.sty
Package: infwarerr 2019/12/03 v1.5 Providing info/warning/error messages
(HO)
) (c:/texlive/2024/texmf-dist/tex/generic/kvdefinekeys/kvdefinekeys.sty
Package: kvdefinekeys 2019-12-19 v1.6 Define keys (HO)
) (c:/texlive/2024/texmf-dist/tex/generic/pdftexcmds/pdftexcmds.sty
Package: pdftexcmds 2020-06-27 v0.33 Utility functions of pdfTeX for
LuaTeX (HO
)
Package pdftexcmds Info: \pdf@primitive is available.
Package pdftexcmds Info: \pdf@ifprimitive is available.
Package pdftexcmds Info: \pdfdraftmode found.
) (c:/texlive/2024/texmf-dist/tex/generic/etexcmds/etexcmds.sty
Package: etexcmds 2019/12/15 v1.7 Avoid name clashes with e-TeX commands
(HO)
) (c:/texlive/2024/texmf-dist/tex/latex/auxhook/auxhook.sty
Package: auxhook 2019-12-17 v1.6 Hooks for auxiliary files (HO)
)
Package zref Info: New property list: main on input line 767.
Package zref Info: New property: default on input line 768.
Package zref Info: New property: page on input line 769.
)
\c@abspage=\count276
Package zref Info: New property: abspage on input line 67.
) (c:/texlive/2024/texmf-dist/tex/latex/needspace/needspace.sty
Package: needspace 2010/09/12 v1.3d reserve vertical space
)
\mdf@templength=\skip71
\c@mdf@globalstyle@cnt=\count277
\mdf@skipabove@length=\skip72
\mdf@skipbelow@length=\skip73
\mdf@leftmargin@length=\skip74
\mdf@rightmargin@length=\skip75
\mdf@innerleftmargin@length=\skip76
\mdf@innerrightmargin@length=\skip77
\mdf@innertopmargin@length=\skip78
\mdf@innerbottommargin@length=\skip79
\mdf@splittopskip@length=\skip80
\mdf@splitbottomskip@length=\skip81
\mdf@outermargin@length=\skip82
\mdf@innermargin@length=\skip83
\mdf@linewidth@length=\skip84
\mdf@innerlinewidth@length=\skip85
```

```
\mdf@middlelinewidth@length=\skip86
\mdf@outerlinewidth@length=\skip87
\mdf@roundcorner@length=\skip88
\mdf@footenotedistance@length=\skip89
\mdf@userdefinedwidth@length=\skip90
\mdf@needspace@length=\skip91
\mdf@frametitleaboveskip@length=\skip92
\mdf@frametitlebelowskip@length=\skip93
\mdf@frametitlerulewidth@length=\skip94
\mdf@frametitleleftmargin@length=\skip95
\mdf@frametitlerightmargin@length=\skip96
\mdf@shadowsize@length=\skip97
\mdf@extratopheight@length=\skip98
\mdf@subtitleabovelinewidth@length=\skip99
\mdf@subtitlebelowlinewidth@length=\skip100
\mdf@subtitleaboveskip@length=\skip101
\mdf@subtitlebelowskip@length=\skip102
\mdf@subtitleinneraboveskip@length=\skip103
\mdf@subtitleinnerbelowskip@length=\skip104
\mdf@subsubtitleabovelinewidth@length=\skip105
\mdf@subsubtitlebelowlinewidth@length=\skip106
\mdf@subsubtitleaboveskip@length=\skip107
\mdf@subsubtitlebelowskip@length=\skip108
\mdf@subsubtitleinneraboveskip@length=\skip109
\mdf@subsubtitleinnerbelowskip@length=\skip110
(c:/texlive/2024/texmf-dist/tex/latex/mdframed/md-frame-0.mdf
File: md-frame-0.mdf 2013/07/01\ 1.9b: md-frame-0
)
\mdf@frametitlebox=\box61
\mdf@footnotebox=\box62
\mdf@splitbox@one=\box63
\mdf@splitbox@two=\box64
\mdf@splitbox@save=\box65
\mdfsplitboxwidth=\skip111
\mdfsplitboxtotalwidth=\skip112
\mdfsplitboxheight=\skip113
\mdfsplitboxdepth=\skip114
\mdfsplitboxtotalheight=\skip115
\mdfframetitleboxwidth=\skip116
\mdfframetitleboxtotalwidth=\skip117
\mdfframetitleboxheight=\skip118
\mdfframetitleboxdepth=\skip119
\mdfframetitleboxtotalheight=\skip120
\mdffootnoteboxwidth=\skip121
\mdffootnoteboxtotalwidth=\skip122
\mdffootnoteboxheight=\skip123
\mdffootnoteboxdepth=\skip124
\mdffootnoteboxtotalheight=\skip125
\mdftotallinewidth=\skip126
\mdfboundingboxwidth=\skip127
\mdfboundingboxtotalwidth=\skip128
\mdfboundingboxheight=\skip129
\mdfboundingboxdepth=\skip130
\mdfboundingboxtotalheight=\skip131
```

```
\mdf@freevspace@length=\skip132
\mdf@horizontalwidthofbox@length=\skip133
\mdf@verticalmarginwhole@length=\skip134
\mdf@horizontalspaceofbox=\skip135
\mdfsubtitleheight=\skip136
\mdfsubsubtitleheight=\skip137
\c@mdfcountframes=\count278

****** mdframed patching \endmdf@trivlist

****** -- success******

\mdf@envdepth=\count279
\c@mdf@env@i=\count280
\c@mdf@env@ii=\count281
\c@mdf@zref@counter=\count282
Package zref Info: New property: mdf@pagevalue on input line 895.
) (c:/texlive/2024/texmf-dist/tex/latex/titlesec/titlesec.sty
Package: titlesec 2023/10/27 v2.16 Sectioning titles
\ttl@box=\box66
\beforetitleunit=\skip138
\aftertitleunit=\skip139
\ttl@plus=\dimen154
\ttl@minus=\dimen155
\ttl@toksa=\toks34
\titlewidth=\dimen156
\titlewidthlast=\dimen157
\titlewidthfirst=\dimen158
) (c:/texlive/2024/texmf-dist/tex/latex/koma-script/scrextend.sty
Package: scrextend 2023/07/07 v3.41 KOMA-Script package (extend other
classes w
ith features of KOMA-Script classes)
(c:/texlive/2024/texmf-dist/tex/latex/koma-script/scrkbase.sty
Package: scrkbase 2023/07/07 v3.41 KOMA-Script package (KOMA-Script-
dependent b
asics and keyval usage)
(c:/texlive/2024/texmf-dist/tex/latex/koma-script/scrbase.sty
Package: scrbase 2023/07/07 v3.41 KOMA-Script package (KOMA-Script-
independent
basics and keyval usage)
(c:/texlive/2024/texmf-dist/tex/latex/koma-script/scrlfile.sty
Package: scrlfile 2023/07/07 v3.41 KOMA-Script package (file load hooks)
(c:/texlive/2024/texmf-dist/tex/latex/koma-script/scrlfile-hook.sty
Package: scrlfile-hook 2023/07/07 v3.41 KOMA-Script package (using LaTeX
hooks)

(c:/texlive/2024/texmf-dist/tex/latex/koma-script/scrlogo.sty
Package: scrlogo 2023/07/07 v3.41 KOMA-Script package (logo)
)))
Applying: [2021/05/01] Usage of raw or classic option list on input line
252.
Already applied: [0000/00/00] Usage of raw or classic option list on
input line
 368.
```

```
))
Package scrextend Info: unexpected definition of `\@makefnmark'.
(scrextend)             Trying to patch it on input line 1762.
Package scrextend Info: patch seems to be successfull on input line 1762.
)

LaTeX Font Warning: Font shape `T1/cmr/m/n' in size <7.5> not available
(Font)              size <7> substituted on input line 69.

(c:/texlive/2024/texmf-dist/tex/latex/tools/calc.sty
Package: calc 2023/07/08 v4.3 Infix arithmetic (KKT,FJ)
\calc@Acount=\count283
\calc@Bcount=\count284
\calc@Adimen=\dimen159
\calc@Bdimen=\dimen160
\calc@Askip=\skip140
\calc@Bskip=\skip141
LaTeX Info: Redefining \setlength on input line 80.
LaTeX Info: Redefining \addtolength on input line 81.
\calc@Ccount=\count285
\calc@Cskip=\skip142
) (c:/texlive/2024/texmf-dist/tex/latex/geometry/geometry.sty
Package: geometry 2020/01/02 v5.9 Page Geometry
(c:/texlive/2024/texmf-dist/tex/generic/iftex/ifvtex.sty
Package: ifvtex 2019/10/25 v1.7 ifvtex legacy package. Use iftex instead.
)
\Gm@cnth=\count286
\Gm@cntv=\count287
\c@Gm@tempcnt=\count288
\Gm@bindingoffset=\dimen161
\Gm@wd@mp=\dimen162
\Gm@odd@mp=\dimen163
\Gm@even@mp=\dimen164
\Gm@layoutwidth=\dimen165
\Gm@layoutheight=\dimen166
\Gm@layouthoffset=\dimen167
\Gm@layoutvoffset=\dimen168
\Gm@dimlist=\toks35
) (c:/texlive/2024/texmf-dist/tex/latex/preprint/authblk.sty
Package: authblk 2001/02/27 1.3 (PWD)
\affilsep=\skip143
\@affilsep=\skip144
\c@Maxaffil=\count289
\c@authors=\count290
\c@affil=\count291
) (c:/texlive/2024/texmf-dist/tex/latex/footmisc/footmisc.sty
Package: footmisc 2023/07/05 v6.0f a miscellany of footnote facilities
\FN@temptoken=\toks36
\footnotemargin=\dimen169
\@outputbox@depth=\dimen170
Package footmisc Info: Declaring symbol style bringhurst on input line
696.
Package footmisc Info: Declaring symbol style chicago on input line 704.
Package footmisc Info: Declaring symbol style wiley on input line 713.
```

```
Package footmisc Info: Declaring symbol style lamport-robust on input
line 724.

Package footmisc Info: Declaring symbol style lamport* on input line 744.
Package footmisc Info: Declaring symbol style lamport*-robust on input
line 765
.
) (c:/texlive/2024/texmf-dist/tex/latex/fancyhdr/fancyhdr.sty
Package: fancyhdr 2024/07/23 v4.3.1 Extensive control of page headers and
foote
rs
\f@nch@headwidth=\skip145
\f@nch@O@elh=\skip146
\f@nch@O@erh=\skip147
\f@nch@O@olh=\skip148
\f@nch@O@orh=\skip149
\f@nch@O@elf=\skip150
\f@nch@O@erf=\skip151
\f@nch@O@olf=\skip152
\f@nch@O@orf=\skip153
) (c:/texlive/2024/texmf-dist/tex/generic/alphalph/alphalph.sty
Package: alphalph 2019/12/09 v2.6 Convert numbers to letters (HO)
(c:/texlive/2024/texmf-dist/tex/generic/intcalc/intcalc.sty
Package: intcalc 2019/12/15 v1.3 Expandable calculations with integers
(HO)
))
\c@authorfn=\count292
(c:/texlive/2024/texmf-dist/tex/latex/abstract/abstract.sty
Package: abstract 2009/06/08 v1.2a configurable abstracts
\abstitleskip=\skip154
\absleftindent=\skip155
\absrightindent=\skip156
\absparindent=\skip157
\absparsep=\skip158
)
Package newfloat Info: New float `keypoints' with options
`placement=t!,name=kp
t' on input line 291.
\c@keypoints=\count293
\newfloat@ftype=\count294
Package newfloat Info: float type `keypoints'=8 on input line 291.
(c:/texlive/2024/texmf-dist/tex/latex/enumitem/enumitem.sty
Package: enumitem 2019/06/20 v3.9 Customized lists
\labelindent=\skip159
\enit@outerparindent=\dimen171
\enit@toks=\toks37
\enit@inbox=\box67
\enit@count@id=\count295
\enitdp@description=\count296
) (c:/texlive/2024/texmf-dist/tex/latex/quoting/quoting.sty
Package: quoting 2014/01/28 v0.1c Consolidated environment for displayed
text
\quo@toppartop=\skip160
) (c:/texlive/2024/texmf-dist/tex/latex/sttools/stfloats.sty
```

```
Package: stfloats 2017/03/27 v3.3 Improve float mechanism and
baselineskip sett
ings
\@dblbotnum=\count297
\c@dblbotnumber=\count298
) (c:/texlive/2024/texmf-dist/tex/latex/booktabs/booktabs.sty
Package: booktabs 2020/01/12 v1.61803398 Publication quality tables
\heavyrulewidth=\dimen172
\lightrulewidth=\dimen173
\cmidrulewidth=\dimen174
\belowrulesep=\dimen175
\belowbottomsep=\dimen176
\aboverulesep=\dimen177
\abovetopsep=\dimen178
\cmidrulesep=\dimen179
\cmidrulekern=\dimen180
\defaultaddspace=\dimen181
\@cmidla=\count299
\@cmidlb=\count300
\@aboverulesep=\dimen182
\@belowrulesep=\dimen183
\@thisruleclass=\count301
\@lastruleclass=\count302
\@thisrulewidth=\dimen184
) (c:/texlive/2024/texmf-dist/tex/latex/tools/tabularx.sty
Package: tabularx 2023/12/11 v2.12a `tabularx' package (DPC)
\TX@col@width=\dimen185
\TX@old@table=\dimen186
\TX@old@col=\dimen187
\TX@target=\dimen188
\TX@delta=\dimen189
\TX@cols=\count303
\TX@ftn=\toks38
)
\enitdp@tablenotes=\count304
(c:/texlive/2024/texmf-dist/tex/latex/caption/caption.sty
Package: caption 2023/08/05 v3.6o Customizing captions (AR)
(c:/texlive/2024/texmf-dist/tex/latex/caption/caption3.sty
Package: caption3 2023/07/31 v2.4d caption3 kernel (AR)
\caption@tempdima=\dimen190
\captionmargin=\dimen191
\caption@leftmargin=\dimen192
\caption@rightmargin=\dimen193
\caption@width=\dimen194
\caption@indent=\dimen195
\caption@parindent=\dimen196
\caption@hangindent=\dimen197
Package caption Info: Standard document class detected.
)
\c@caption@flags=\count305
\c@continuedfloat=\count306
Package caption Info: rotating package is loaded.
Package caption Info: scrextend package is loaded.
\caption@addmargin@hsize=\dimen198
```

```
\caption@addmargin@linewidth=\dimen199
) (c:/texlive/2024/texmf-dist/tex/latex/natbib/natbib.sty
Package: natbib 2010/09/13 8.31b (PWD, AO)
\bibhang=\skip161
\bibsep=\skip162
LaTeX Info: Redefining \cite on input line 694.
\c@NAT@ctr=\count307
)) (c:/texlive/2024/texmf-dist/tex/latex/siunitx/siunitx.sty
Package: siunitx 2024-06-24 v3.3.19 A comprehensive (SI) units package
\l__siunitx_number_uncert_offset_int=\count308
\l__siunitx_number_exponent_fixed_int=\count309
\l__siunitx_number_min_decimal_int=\count310
\l__siunitx_number_min_integer_int=\count311
\l__siunitx_number_round_precision_int=\count312
\l__siunitx_number_lower_threshold_int=\count313
\l__siunitx_number_upper_threshold_int=\count314
\l__siunitx_number_group_first_int=\count315
\l__siunitx_number_group_size_int=\count316
\l__siunitx_number_group_minimum_int=\count317
\l__siunitx_angle_tmp_dim=\dimen256
\l__siunitx_angle_marker_box=\box68
\l__siunitx_angle_unit_box=\box69
\l__siunitx_compound_count_int=\count318
(c:/texlive/2024/texmf-dist/tex/latex/translations/translations.sty
Package: translations 2022/02/05 v1.12 internationalization of LaTeX2e
packages
 (CN)
) (c:/texlive/2024/texmf-dist/tex/latex/amsmath/amstext.sty
Package: amstext 2021/08/26 v2.01 AMS text
(c:/texlive/2024/texmf-dist/tex/latex/amsmath/amsgen.sty
File: amsgen.sty 1999/11/30 v2.0 generic functions
\@emptytoks=\toks39
\ex@=\dimen257
))
\l__siunitx_table_tmp_box=\box70
\l__siunitx_table_tmp_dim=\dimen258
\l__siunitx_table_column_width_dim=\dimen259
\l__siunitx_table_integer_box=\box71
\l__siunitx_table_decimal_box=\box72
\l__siunitx_table_uncert_box=\box73
\l__siunitx_table_before_box=\box74
\l__siunitx_table_after_box=\box75
\l__siunitx_table_before_dim=\dimen260
\l__siunitx_table_carry_dim=\dimen261
\l__siunitx_unit_tmp_int=\count319
\l__siunitx_unit_position_int=\count320
\l__siunitx_unit_total_int=\count321
) (c:/texlive/2024/texmf-dist/tex/latex/amsfonts/amssymb.sty
Package: amssymb 2013/01/14 v3.01 AMS font symbols
(c:/texlive/2024/texmf-dist/tex/latex/amsfonts/amsfonts.sty
Package: amsfonts 2013/01/14 v3.01 Basic AMSFonts support
\symAMSa=\mathgroup10
\symAMSb=\mathgroup11
LaTeX Font Info:    Redeclaring math symbol \hbar on input line 98.
```

```
LaTeX Info: Redefining \frak on input line 111.
)) (c:/texlive/2024/texmf-dist/tex/latex/amsmath/amsmath.sty
Package: amsmath 2024/05/23 v2.17q AMS math features
\@mathmargin=\skip163
For additional information on amsmath, use the `?' option.
(c:/texlive/2024/texmf-dist/tex/latex/amsmath/amsbsy.sty
Package: amsbsy 1999/11/29 v1.2d Bold Symbols
\pmbraise@=\dimen262
) (c:/texlive/2024/texmf-dist/tex/latex/amsmath/amsopn.sty
Package: amsopn 2022/04/08 v2.04 operator names
)
\inf@bad=\count322
LaTeX Info: Redefining \frac on input line 233.
\uproot@=\count323
\leftroot@=\count324
LaTeX Info: Redefining \overline on input line 398.
LaTeX Info: Redefining \colon on input line 409.
\classnum@=\count325
\DOTSCASE@=\count326
LaTeX Info: Redefining \ldots on input line 495.
LaTeX Info: Redefining \dots on input line 498.
LaTeX Info: Redefining \cdots on input line 619.
\Mathstrutbox@=\box76
\strutbox@=\box77
LaTeX Info: Redefining \big on input line 721.
LaTeX Info: Redefining \Big on input line 722.
LaTeX Info: Redefining \bigg on input line 723.
LaTeX Info: Redefining \Bigg on input line 724.
\big@size=\dimen263
LaTeX Font Info:    Redeclaring font encoding OML on input line 742.
LaTeX Font Info:    Redeclaring font encoding OMS on input line 743.
\macc@depth=\count327
LaTeX Info: Redefining \bmod on input line 904.
LaTeX Info: Redefining \pmod on input line 909.
LaTeX Info: Redefining \smash on input line 939.
LaTeX Info: Redefining \relbar on input line 969.
LaTeX Info: Redefining \Relbar on input line 970.
\c@MaxMatrixCols=\count328
\dotsspace@=\muskip21
\c@parentequation=\count329
\dspbrk@lvl=\count330
\tag@help=\toks40
\row@=\count331
\column@=\count332
\maxfields@=\count333
\andhelp@=\toks41
\eqnshift@=\dimen264
\alignsep@=\dimen265
\tagshift@=\dimen266
\tagwidth@=\dimen267
\totwidth@=\dimen268
\lineht@=\dimen269
\@envbody=\toks42
\multlinegap=\skip164
```

```
\multlinetaggap=\skip165
\mathdisplay@stack=\toks43
LaTeX Info: Redefining \[ on input line 2953.
LaTeX Info: Redefining \] on input line 2954.
) (c:/texlive/2024/texmf-dist/tex/latex/multirow/multirow.sty
Package: multirow 2021/03/15 v2.8 Span multiple rows of a table
\multirow@colwidth=\skip166
\multirow@cntb=\count334
\multirow@dima=\skip167
\bigstrutjot=\dimen270
) (c:/texlive/2024/texmf-dist/tex/latex/comment/comment.sty
\CommentStream=\write3
Excluding comment 'comment') (c:/texlive/2024/texmf-
dist/tex/latex/orcidlink/or
cidlink.sty
Package: orcidlink 2024/06/26 v1.1.0 Support ORCID's three different ID
formats
.
(c:/texlive/2024/texmf-dist/tex/latex/hyperref/hyperref.sty
Package: hyperref 2024-07-10 v7.01j Hypertext links for LaTeX
(c:/texlive/2024/texmf-dist/tex/generic/pdfescape/pdfescape.sty
Package: pdfescape 2019/12/09 v1.15 Implements pdfTeX's escape features
(HO)
) (c:/texlive/2024/texmf-dist/tex/latex/hycolor/hycolor.sty
Package: hycolor 2020-01-27 v1.10 Color options for hyperref/bookmark
(HO)
) (c:/texlive/2024/texmf-dist/tex/latex/hyperref/nameref.sty
Package: nameref 2023-11-26 v2.56 Cross-referencing by name of section
(c:/texlive/2024/texmf-dist/tex/latex/refcount/refcount.sty
Package: refcount 2019/12/15 v3.6 Data extraction from label references
(HO)
) (c:/texlive/2024/texmf-
dist/tex/generic/gettitlestring/gettitlestring.sty
Package: gettitlestring 2019/12/15 v1.6 Cleanup title references (HO)
)
\c@section@level=\count335
) (c:/texlive/2024/texmf-dist/tex/generic/stringenc/stringenc.sty
Package: stringenc 2019/11/29 v1.12 Convert strings between diff.
encodings (HO
)
)
\@linkdim=\dimen271
\Hy@linkcounter=\count336
\Hy@pagecounter=\count337
(c:/texlive/2024/texmf-dist/tex/latex/hyperref/pd1enc.def
File: pd1enc.def 2024-07-10 v7.01j Hyperref: PDFDocEncoding definition
(HO)
Now handling font encoding PD1 ...
... no UTF-8 mapping file for font encoding PD1
)
\Hy@SavedSpaceFactor=\count338
(c:/texlive/2024/texmf-dist/tex/latex/hyperref/puenc.def
File: puenc.def 2024-07-10 v7.01j Hyperref: PDF Unicode definition (HO)
Now handling font encoding PU ...
```

```
... no UTF-8 mapping file for font encoding PU
)
Package hyperref Info: Hyper figures OFF on input line 4157.
Package hyperref Info: Link nesting OFF on input line 4162.
Package hyperref Info: Hyper index ON on input line 4165.
Package hyperref Info: Plain pages OFF on input line 4172.
Package hyperref Info: Backreferencing OFF on input line 4177.
Package hyperref Info: Implicit mode ON; LaTeX internals redefined.
Package hyperref Info: Bookmarks ON on input line 4424.
\c@Hy@tempcnt=\count339
LaTeX Info: Redefining \url on input line 4763.
\XeTeXLinkMargin=\dimen272
(c:/texlive/2024/texmf-dist/tex/generic/bitset/bitset.sty
Package: bitset 2019/12/09 v1.3 Handle bit-vector datatype (HO)
(c:/texlive/2024/texmf-dist/tex/generic/bigintcalc/bigintcalc.sty
Package: bigintcalc 2019/12/15 v1.5 Expandable calculations on big
integers (HO
)
))
\Fld@menulength=\count340
\Field@Width=\dimen273
\Fld@charsize=\dimen274
Package hyperref Info: Hyper figures OFF on input line 6042.
Package hyperref Info: Link nesting OFF on input line 6047.
Package hyperref Info: Hyper index ON on input line 6050.
Package hyperref Info: backreferencing OFF on input line 6057.
Package hyperref Info: Link coloring OFF on input line 6062.
Package hyperref Info: Link coloring with OCG OFF on input line 6067.
Package hyperref Info: PDF/A mode OFF on input line 6072.
(c:/texlive/2024/texmf-dist/tex/latex/base/atbegshi-ltx.sty
Package: atbegshi-ltx 2021/01/10 v1.0c Emulation of the original atbegshi
package with kernel methods
)
\Hy@abspage=\count341
\c@Item=\count342
\c@Hfootnote=\count343
)
Package hyperref Info: Driver (autodetected): hpdftex.
(c:/texlive/2024/texmf-dist/tex/latex/hyperref/hpdftex.def
File: hpdftex.def 2024-07-10 v7.01j Hyperref driver for pdfTeX
(c:/texlive/2024/texmf-dist/tex/latex/base/atveryend-ltx.sty
Package: atveryend-ltx 2020/08/19 v1.0a Emulation of the original
atveryend pac
kage
with kernel methods
)
\HyAnn@Count=\count344
\Fld@listcount=\count345
\c@bookmark@seq@number=\count346
(c:/texlive/2024/texmf-dist/tex/latex/rerunfilecheck/rerunfilecheck.sty
Package: rerunfilecheck 2022-07-10 v1.10 Rerun checks for auxiliary files
(HO)
(c:/texlive/2024/texmf-dist/tex/generic/uniquecounter/uniquecounter.sty
```

```
Package: uniquecounter 2019/12/15 v1.4 Provide unlimited unique counter
(HO)
)
Package uniquecounter Info: New unique counter `rerunfilecheck' on input
line 2
85.
)
\Hy@SectionHShift=\skip168
) (c:/texlive/2024/texmf-dist/tex/latex/pgf/frontendlayer/tikz.sty
(c:/texlive/
2024/texmf-dist/tex/latex/pgf/basiclayer/pgf.sty (c:/texlive/2024/texmf-
dist/te
x/latex/pgf/utilities/pgfrcs.sty (c:/texlive/2024/texmf-
dist/tex/generic/pgf/ut
ilities/pgfutil-common.tex
\pgfutil@everybye=\toks44
\pgfutil@tempdima=\dimen275
\pgfutil@tempdimb=\dimen276
) (c:/texlive/2024/texmf-dist/tex/generic/pgf/utilities/pgfutil-latex.def
\pgfutil@abb=\box78
) (c:/texlive/2024/texmf-dist/tex/generic/pgf/utilities/pgfrcs.code.tex
(c:/tex
live/2024/texmf-dist/tex/generic/pgf/pgf.revision.tex)
Package: pgfrcs 2023-01-15 v3.1.10 (3.1.10)
))
Package: pgf 2023-01-15 v3.1.10 (3.1.10)
(c:/texlive/2024/texmf-dist/tex/latex/pgf/basiclayer/pgfcore.sty
(c:/texlive/20
24/texmf-dist/tex/latex/pgf/systemlayer/pgfsys.sty
(c:/texlive/2024/texmf-dist/
tex/generic/pgf/systemlayer/pgfsys.code.tex
Package: pgfsys 2023-01-15 v3.1.10 (3.1.10)
(c:/texlive/2024/texmf-dist/tex/generic/pgf/utilities/pgfkeys.code.tex
\pgfkeys@pathtoks=\toks45
\pgfkeys@temptoks=\toks46

(c:/texlive/2024/texmf-
dist/tex/generic/pgf/utilities/pgfkeyslibraryfiltered.co
de.tex
\pgfkeys@tmptoks=\toks47
))
\pgf@x=\dimen277
\pgf@y=\dimen278
\pgf@xa=\dimen279
\pgf@ya=\dimen280
\pgf@xb=\dimen281
\pgf@yb=\dimen282
\pgf@xc=\dimen283
\pgf@yc=\dimen284
\pgf@xd=\dimen285
\pgf@yd=\dimen286
\w@pgf@writea=\write4
\r@pgf@reada=\read2
\c@pgf@counta=\count347
```

```
\c@pgf@countb=\count348
\c@pgf@countc=\count349
\c@pgf@countd=\count350
\t@pgf@toka=\toks48
\t@pgf@tokb=\toks49
\t@pgf@tokc=\toks50
\pgf@sys@id@count=\count351
(c:/texlive/2024/texmf-dist/tex/generic/pgf/systemlayer/pgf.cfg
File: pgf.cfg 2023-01-15 v3.1.10 (3.1.10)
)
Driver file for pgf: pgfsys-pdftex.def
(c:/texlive/2024/texmf-dist/tex/generic/pgf/systemlayer/pgfsys-pdftex.def
File: pgfsys-pdftex.def 2023-01-15 v3.1.10 (3.1.10)
(c:/texlive/2024/texmf-dist/tex/generic/pgf/systemlayer/pgfsys-common-
pdf.def
File: pgfsys-common-pdf.def 2023-01-15 v3.1.10 (3.1.10)
)))
(c:/texlive/2024/texmf-
dist/tex/generic/pgf/systemlayer/pgfsyssoftpath.code.tex
File: pgfsyssoftpath.code.tex 2023-01-15 v3.1.10 (3.1.10)
\pgfsyssoftpath@smallbuffer@items=\count352
\pgfsyssoftpath@bigbuffer@items=\count353
)
(c:/texlive/2024/texmf-
dist/tex/generic/pgf/systemlayer/pgfsysprotocol.code.tex
File: pgfsysprotocol.code.tex 2023-01-15 v3.1.10 (3.1.10)
)) (c:/texlive/2024/texmf-
dist/tex/generic/pgf/basiclayer/pgfcore.code.tex
Package: pgfcore 2023-01-15 v3.1.10 (3.1.10)
(c:/texlive/2024/texmf-dist/tex/generic/pgf/math/pgfmath.code.tex
(c:/texlive/2
024/texmf-dist/tex/generic/pgf/math/pgfmathutil.code.tex)
(c:/texlive/2024/texm
f-dist/tex/generic/pgf/math/pgfmathparser.code.tex
\pgfmath@dimen=\dimen287
\pgfmath@count=\count354
\pgfmath@box=\box79
\pgfmath@toks=\toks51
\pgfmath@stack@operand=\toks52
\pgfmath@stack@operation=\toks53
) (c:/texlive/2024/texmf-
dist/tex/generic/pgf/math/pgfmathfunctions.code.tex)
(c:/texlive/2024/texmf-
dist/tex/generic/pgf/math/pgfmathfunctions.basic.code.te
x)
(c:/texlive/2024/texmf-
dist/tex/generic/pgf/math/pgfmathfunctions.trigonometric
.code.tex)
(c:/texlive/2024/texmf-
dist/tex/generic/pgf/math/pgfmathfunctions.random.code.t
ex)
(c:/texlive/2024/texmf-
dist/tex/generic/pgf/math/pgfmathfunctions.comparison.co
de.tex)
```

```
(c:/texlive/2024/texmf-
dist/tex/generic/pgf/math/pgfmathfunctions.base.code.tex
)
(c:/texlive/2024/texmf-
dist/tex/generic/pgf/math/pgfmathfunctions.round.code.te
x)
(c:/texlive/2024/texmf-
dist/tex/generic/pgf/math/pgfmathfunctions.misc.code.tex
)
(c:/texlive/2024/texmf-
dist/tex/generic/pgf/math/pgfmathfunctions.integerarithm
etics.code.tex) (c:/texlive/2024/texmf-
dist/tex/generic/pgf/math/pgfmathcalc.co
de.tex) (c:/texlive/2024/texmf-
dist/tex/generic/pgf/math/pgfmathfloat.code.tex
\c@pgfmathroundto@lastzeros=\count355
)) (c:/texlive/2024/texmf-dist/tex/generic/pgf/math/pgfint.code.tex)
(c:/texliv
e/2024/texmf-dist/tex/generic/pgf/basiclayer/pgfcorepoints.code.tex
File: pgfcorepoints.code.tex 2023-01-15 v3.1.10 (3.1.10)
\pgf@picminx=\dimen288
\pgf@picmaxx=\dimen289
\pgf@picminy=\dimen290
\pgf@picmaxy=\dimen291
\pgf@pathminx=\dimen292
\pgf@pathmaxx=\dimen293
\pgf@pathminy=\dimen294
\pgf@pathmaxy=\dimen295
\pgf@xx=\dimen296
\pgf@xy=\dimen297
\pgf@yx=\dimen298
\pgf@yy=\dimen299
\pgf@zx=\dimen300
\pgf@zy=\dimen301
)
(c:/texlive/2024/texmf-
dist/tex/generic/pgf/basiclayer/pgfcorepathconstruct.cod
e.tex
File: pgfcorepathconstruct.code.tex 2023-01-15 v3.1.10 (3.1.10)
\pgf@path@lastx=\dimen302
\pgf@path@lasty=\dimen303
)
(c:/texlive/2024/texmf-
dist/tex/generic/pgf/basiclayer/pgfcorepathusage.code.te
x
File: pgfcorepathusage.code.tex 2023-01-15 v3.1.10 (3.1.10)
\pgf@shorten@end@additional=\dimen304
\pgf@shorten@start@additional=\dimen305
) (c:/texlive/2024/texmf-
dist/tex/generic/pgf/basiclayer/pgfcorescopes.code.tex
File: pgfcorescopes.code.tex 2023-01-15 v3.1.10 (3.1.10)
\pgfpic=\box80
\pgf@hbox=\box81
\pgf@layerbox@main=\box82
```

```
\pgf@picture@serial@count=\count356
)
(c:/texlive/2024/texmf-
dist/tex/generic/pgf/basiclayer/pgfcoregraphicstate.code
.tex
File: pgfcoregraphicstate.code.tex 2023-01-15 v3.1.10 (3.1.10)
\pgflinewidth=\dimen306
)
(c:/texlive/2024/texmf-
dist/tex/generic/pgf/basiclayer/pgfcoretransformations.c
ode.tex
File: pgfcoretransformations.code.tex 2023-01-15 v3.1.10 (3.1.10)
\pgf@pt@x=\dimen307
\pgf@pt@y=\dimen308
\pgf@pt@temp=\dimen309
) (c:/texlive/2024/texmf-
dist/tex/generic/pgf/basiclayer/pgfcorequick.code.tex
File: pgfcorequick.code.tex 2023-01-15 v3.1.10 (3.1.10)
) (c:/texlive/2024/texmf-
dist/tex/generic/pgf/basiclayer/pgfcoreobjects.code.te
x
File: pgfcoreobjects.code.tex 2023-01-15 v3.1.10 (3.1.10)
)
(c:/texlive/2024/texmf-
dist/tex/generic/pgf/basiclayer/pgfcorepathprocessing.co
de.tex
File: pgfcorepathprocessing.code.tex 2023-01-15 v3.1.10 (3.1.10)
) (c:/texlive/2024/texmf-
dist/tex/generic/pgf/basiclayer/pgfcorearrows.code.tex
File: pgfcorearrows.code.tex 2023-01-15 v3.1.10 (3.1.10)
\pgfarrowsep=\dimen310
) (c:/texlive/2024/texmf-
dist/tex/generic/pgf/basiclayer/pgfcoreshade.code.tex
File: pgfcoreshade.code.tex 2023-01-15 v3.1.10 (3.1.10)
\pgf@max=\dimen311
\pgf@sys@shading@range@num=\count357
\pgf@shadingcount=\count358
) (c:/texlive/2024/texmf-
dist/tex/generic/pgf/basiclayer/pgfcoreimage.code.tex
File: pgfcoreimage.code.tex 2023-01-15 v3.1.10 (3.1.10)
)
(c:/texlive/2024/texmf-
dist/tex/generic/pgf/basiclayer/pgfcoreexternal.code.tex
File: pgfcoreexternal.code.tex 2023-01-15 v3.1.10 (3.1.10)
\pgfexternal@startupbox=\box83
) (c:/texlive/2024/texmf-
dist/tex/generic/pgf/basiclayer/pgfcorelayers.code.tex
File: pgfcorelayers.code.tex 2023-01-15 v3.1.10 (3.1.10)
)
(c:/texlive/2024/texmf-
dist/tex/generic/pgf/basiclayer/pgfcoretransparency.code
.tex
File: pgfcoretransparency.code.tex 2023-01-15 v3.1.10 (3.1.10)
)
```

```
(c:/texlive/2024/texmf-
dist/tex/generic/pgf/basiclayer/pgfcorepatterns.code.tex
File: pgfcorepatterns.code.tex 2023-01-15 v3.1.10 (3.1.10)
) (c:/texlive/2024/texmf-
dist/tex/generic/pgf/basiclayer/pgfcorerdf.code.tex
File: pgfcorerdf.code.tex 2023-01-15 v3.1.10 (3.1.10)
))) (c:/texlive/2024/texmf-
dist/tex/generic/pgf/modules/pgfmoduleshapes.code.te
x
File: pgfmoduleshapes.code.tex 2023-01-15 v3.1.10 (3.1.10)
\pgfnodeparttextbox=\box84
) (c:/texlive/2024/texmf-
dist/tex/generic/pgf/modules/pgfmoduleplot.code.tex
File: pgfmoduleplot.code.tex 2023-01-15 v3.1.10 (3.1.10)
)
(c:/texlive/2024/texmf-dist/tex/latex/pgf/compatibility/pgfcomp-version-
0-65.st
y
Package: pgfcomp-version-0-65 2023-01-15 v3.1.10 (3.1.10)
\pgf@nodesepstart=\dimen312
\pgf@nodesepend=\dimen313
)
(c:/texlive/2024/texmf-dist/tex/latex/pgf/compatibility/pgfcomp-version-
1-18.st
y
Package: pgfcomp-version-1-18 2023-01-15 v3.1.10 (3.1.10)
)) (c:/texlive/2024/texmf-dist/tex/latex/pgf/utilities/pgffor.sty
(c:/texlive/2
024/texmf-dist/tex/latex/pgf/utilities/pgfkeys.sty
(c:/texlive/2024/texmf-dist/
tex/generic/pgf/utilities/pgfkeys.code.tex)) (c:/texlive/2024/texmf-
dist/tex/la
tex/pgf/math/pgfmath.sty (c:/texlive/2024/texmf-
dist/tex/generic/pgf/math/pgfma
th.code.tex)) (c:/texlive/2024/texmf-
dist/tex/generic/pgf/utilities/pgffor.code
.tex
Package: pgffor 2023-01-15 v3.1.10 (3.1.10)
\pgffor@iter=\dimen314
\pgffor@skip=\dimen315
\pgffor@stack=\toks54
\pgffor@toks=\toks55
)) (c:/texlive/2024/texmf-
dist/tex/generic/pgf/frontendlayer/tikz/tikz.code.tex
Package: tikz 2023-01-15 v3.1.10 (3.1.10)

(c:/texlive/2024/texmf-
dist/tex/generic/pgf/libraries/pgflibraryplothandlers.co
de.tex
File: pgflibraryplothandlers.code.tex 2023-01-15 v3.1.10 (3.1.10)
\pgf@plot@mark@count=\count359
\pgfplotmarksize=\dimen316
)
\tikz@lastx=\dimen317
```

```
\tikz@lasty=\dimen318
\tikz@lastxsaved=\dimen319
\tikz@lastysaved=\dimen320
\tikz@lastmovetox=\dimen321
\tikz@lastmovetoy=\dimen322
\tikzleveldistance=\dimen323
\tikzsiblingdistance=\dimen324
\tikz@figbox=\box85
\tikz@figbox@bg=\box86
\tikz@tempbox=\box87
\tikz@tempbox@bg=\box88
\tikztreelevel=\count360
\tikznumberofchildren=\count361
\tikznumberofcurrentchild=\count362
\tikz@fig@count=\count363
(c:/texlive/2024/texmf-
dist/tex/generic/pgf/modules/pgfmodulematrix.code.tex
File: pgfmodulematrix.code.tex 2023-01-15 v3.1.10 (3.1.10)
\pgfmatrixcurrentrow=\count364
\pgfmatrixcurrentcolumn=\count365
\pgf@matrix@numberofcolumns=\count366
)
\tikz@expandcount=\count367

(c:/texlive/2024/texmf-
dist/tex/generic/pgf/frontendlayer/tikz/libraries/tikzli
brarytopaths.code.tex
File: tikzlibrarytopaths.code.tex 2023-01-15 v3.1.10 (3.1.10)
)))
(c:/texlive/2024/texmf-
dist/tex/generic/pgf/frontendlayer/tikz/libraries/tikzli
brarysvg.path.code.tex
File: tikzlibrarysvg.path.code.tex 2023-01-15 v3.1.10 (3.1.10)

(c:/texlive/2024/texmf-
dist/tex/generic/pgf/libraries/pgflibrarysvg.path.code.t
ex
File: pgflibrarysvg.path.code.tex 2023-01-15 v3.1.10 (3.1.10)
(c:/texlive/2024/texmf-
dist/tex/generic/pgf/modules/pgfmoduleparser.code.tex
File: pgfmoduleparser.code.tex 2023-01-15 v3.1.10 (3.1.10)
\pgfparserdef@arg@count=\count368
)
\pgf@lib@svg@last@x=\dimen325
\pgf@lib@svg@last@y=\dimen326
\pgf@lib@svg@last@c@x=\dimen327
\pgf@lib@svg@last@c@y=\dimen328
\pgf@lib@svg@count=\count369
\pgf@lib@svg@max@num=\count370
))
\@curXheight=\skip169
)

! LaTeX Error: Option clash for package hyperref.
```

```
See the LaTeX manual or LaTeX Companion for explanation.
Type  H <return>  for immediate help.
 ...

l.30 \begin{document}

The package hyperref has already been loaded with options:
  []
There has now been an attempt to load it with options
  [colorlinks,allcolors=black,urlcolor=blue]
Adding the global options:
  ,colorlinks,allcolors=black,urlcolor=blue
to your \documentclass declaration may fix this.
Try typing  <return>  to proceed.

Package translations Info: No language package found. I am going to use
`englis
h' as default language. on input line 30.
LaTeX Font Info:    Trying to load font information for T1+Merriwthr-OsF
on inp
ut line 30.
(c:/texlive/2024/texmf-dist/tex/latex/merriweather/T1Merriwthr-OsF.fd
File: T1Merriwthr-OsF.fd 2020/08/30 (autoinst) Font definitions for
T1/Merriwth
r-OsF.
)
LaTeX Font Info:    Font shape `T1/Merriwthr-OsF/m/n' will be
(Font)              scaled to size 7.5pt on input line 30.
(./main.aux)
\openout1 = `main.aux'.

LaTeX Font Info:    Checking defaults for OML/cmm/m/it on input line 30.
LaTeX Font Info:    ... okay on input line 30.
LaTeX Font Info:    Checking defaults for OMS/cmsy/m/n on input line 30.
LaTeX Font Info:    ... okay on input line 30.
LaTeX Font Info:    Checking defaults for OT1/cmr/m/n on input line 30.
LaTeX Font Info:    ... okay on input line 30.
LaTeX Font Info:    Checking defaults for T1/cmr/m/n on input line 30.
LaTeX Font Info:    ... okay on input line 30.
LaTeX Font Info:    Checking defaults for TS1/cmr/m/n on input line 30.
LaTeX Font Info:    ... okay on input line 30.
LaTeX Font Info:    Checking defaults for OMX/cmex/m/n on input line 30.
LaTeX Font Info:    ... okay on input line 30.
LaTeX Font Info:    Checking defaults for U/cmr/m/n on input line 30.
LaTeX Font Info:    ... okay on input line 30.
LaTeX Font Info:    Checking defaults for PD1/pdf/m/n on input line 30.
LaTeX Font Info:    ... okay on input line 30.
LaTeX Font Info:    Checking defaults for PU/pdf/m/n on input line 30.
LaTeX Font Info:    ... okay on input line 30.
LaTeX Info: Redefining \microtypecontext on input line 30.
Package microtype Info: Applying patch `item' on input line 30.
Package microtype Info: Applying patch `toc' on input line 30.
Package microtype Info: Applying patch `eqnum' on input line 30.
```

```
Package microtype Info: Applying patch `footnote' on input line 30.
Package microtype Info: Applying patch `verbatim' on input line 30.
Package microtype Info: Generating PDF output.
Package microtype Info: Character protrusion enabled (level 2).
Package microtype Info: Using default protrusion set `alltext'.
Package microtype Info: Automatic font expansion enabled (level 2),
(microtype)             stretch: 20, shrink: 20, step: 1, non-selected.
Package microtype Info: Using default expansion set `alltext-nott'.
LaTeX Info: Redefining \showhyphens on input line 30.
Package microtype Info: No adjustment of tracking.
Package microtype Info: No adjustment of interword spacing.
Package microtype Info: No adjustment of character kerning.
Package microtype Info: Loading generic protrusion settings for font
family
(microtype)             `Merriwthr-OsF' (encoding: T1).
(microtype)             For optimal results, create family-specific
settings.
(microtype)             See the microtype manual for details.
LaTeX Font Info:    Redeclaring symbol font `operators' on input line 30.
LaTeX Font Info:    Encoding `OT1' has changed to `T1' for symbol font
(Font)              `operators' in the math version `normal' on input
line 30.
LaTeX Font Info:    Overwriting symbol font `operators' in version
`normal'
(Font)                  OT1/cmr/m/n --> T1/Merriwthr-OsF/m/up on input
line 30.

LaTeX Font Info:    Encoding `OT1' has changed to `T1' for symbol font
(Font)              `operators' in the math version `bold' on input line
30.
LaTeX Font Info:    Overwriting symbol font `operators' in version `bold'
(Font)                  OT1/cmr/bx/n --> T1/Merriwthr-OsF/m/up on input
line 30
.
LaTeX Font Info:    Overwriting symbol font `operators' in version `bold'
(Font)                  T1/Merriwthr-OsF/m/up --> T1/Merriwthr-OsF/b/up
on inpu
t line 30.
LaTeX Font Info:    Redeclaring math alphabet \mathbf on input line 30.
LaTeX Font Info:    Overwriting math alphabet `\mathbf' in version
`normal'
(Font)                  OT1/cmr/bx/n --> T1/Merriwthr-OsF/b/up on input
line 30
.
LaTeX Font Info:    Overwriting math alphabet `\mathbf' in version `bold'
(Font)                  OT1/cmr/bx/n --> T1/Merriwthr-OsF/b/up on input
line 30
.
LaTeX Font Info:    Redeclaring math alphabet \mathsf on input line 30.
LaTeX Font Info:    Overwriting math alphabet `\mathsf' in version
`normal'
(Font)                  OT1/cmss/m/n --> T1/MerriwthrSans-OsF/m/up on
input lin
e 30.
```

```
LaTeX Font Info:    Overwriting math alphabet `\mathsf' in version `bold'
(Font)                  OT1/cmss/bx/n --> T1/MerriwthrSans-OsF/m/up on
input li
ne 30.
LaTeX Font Info:    Redeclaring math alphabet \mathit on input line 30.
LaTeX Font Info:    Overwriting math alphabet `\mathit' in version
`normal'
(Font)                  OT1/cmr/m/it --> T1/Merriwthr-OsF/m/it on input
line 30
.
LaTeX Font Info:    Overwriting math alphabet `\mathit' in version `bold'
(Font)                  OT1/cmr/bx/it --> T1/Merriwthr-OsF/m/it on input
line 3
0.
LaTeX Font Info:    Redeclaring math alphabet \mathtt on input line 30.
LaTeX Font Info:    Overwriting math alphabet `\mathtt' in version
`normal'
(Font)                  OT1/cmtt/m/n --> T1/lmtt/m/up on input line 30.
LaTeX Font Info:    Overwriting math alphabet `\mathtt' in version `bold'
(Font)                  OT1/cmtt/m/n --> T1/lmtt/m/up on input line 30.
LaTeX Font Info:    Overwriting math alphabet `\mathsf' in version `bold'
(Font)                  T1/MerriwthrSans-OsF/m/up --> T1/MerriwthrSans-
OsF/b/up
 on input line 30.
LaTeX Font Info:    Overwriting math alphabet `\mathit' in version `bold'
(Font)                  T1/Merriwthr-OsF/m/it --> T1/Merriwthr-OsF/b/it
on inpu
t line 30.
\c@mv@tabular=\count371
\c@mv@boldtabular=\count372
(c:/texlive/2024/texmf-dist/tex/context/base/mkii/supp-pdf.mkii
[Loading MPS to PDF converter (version 2006.09.02).]
\scratchcounter=\count373
\scratchdimen=\dimen329
\scratchbox=\box89
\nofMPsegments=\count374
\nofMParguments=\count375
\everyMPshowfont=\toks56
\MPscratchCnt=\count376
\MPscratchDim=\dimen330
\MPnumerator=\count377
\makeMPintoPDFobject=\count378
\everyMPtoPDFconversion=\toks57
) (c:/texlive/2024/texmf-dist/tex/latex/epstopdf-pkg/epstopdf-base.sty
Package: epstopdf-base 2020-01-24 v2.11 Base part for package epstopdf
Package epstopdf-base Info: Redefining graphics rule for `.eps' on input
line 4
85.
(c:/texlive/2024/texmf-dist/tex/latex/latexconfig/epstopdf-sys.cfg
File: epstopdf-sys.cfg 2010/07/13 v1.3 Configuration of (r)epstopdf for
TeX Liv
e
))
*geometry* driver: auto-detecting
```

```
*geometry* detected driver: pdftex
*geometry* verbose mode - [ preamble ] result:
* driver: pdftex
* paper: a4paper
* layout: <same size as paper>
* layoutoffset:(h,v)=(0.0pt,0.0pt)
* modes: includefoot twoside
* h-part:(L,W,R)=(54.64pt, 488.22787pt, 54.64pt)
* v-part:(T,H,B)=(66.0pt, 745.04684pt, 34.0pt)
* \paperwidth=597.50787pt
* \paperheight=845.04684pt
* \textwidth=488.22787pt
* \textheight=715.04684pt
* \oddsidemargin=-17.62999pt
* \evensidemargin=-17.62999pt
* \topmargin=-47.76999pt
* \headheight=17.5pt
* \headsep=24.0pt
* \topskip=10.0pt
* \footskip=30.0pt
* \marginparwidth=48.0pt
* \marginparsep=10.0pt
* \columnsep=18.0pt
* \skip\footins=22.0pt plus 2.0pt
* \hoffset=0.0pt
* \voffset=0.0pt
* \mag=1000
* \@twocolumntrue
* \@twosidetrue
* \@mparswitchtrue
* \@reversemarginfalse
* (1in=72.27pt=25.4mm, 1cm=28.453pt)

Package caption Info: Begin \AtBeginDocument code.
Package caption Info: hyperref package is loaded.
Package caption Info: End \AtBeginDocument code.

(c:/texlive/2024/texmf-dist/tex/latex/translations/translations-basic-
dictionar
y-english.trsl
File: translations-basic-dictionary-english.trsl (english translation
file `tra
nslations-basic-dictionary')
)
Package translations Info: loading dictionary `translations-basic-
dictionary' f
or `english'. on input line 30.
Package hyperref Info: Link coloring OFF on input line 30.
(./main.out) (./main.out)
\@outlinefile=\write5
\openout5 = `main.out'.

\@gsciteddetails=\box90
\@gscitedetailsheight=\skip170
```

```
\@gsheadbox=\box91
\@gsheadboxheight=\skip171
LaTeX Font Info:    Font shape `T1/Merriwthr-OsF/b/n' will be
(Font)              scaled to size 6.5pt on input line 30.
LaTeX Font Info:    Calculating math sizes for size <7.5> on input line
30.

LaTeX Font Warning: Font shape `T1/Merriwthr-OsF/m/up' undefined
(Font)              using `T1/Merriwthr-OsF/m/n' instead on input line
30.

LaTeX Font Info:    Font shape `T1/Merriwthr-OsF/m/up' will be
(Font)              scaled to size 6.24973pt on input line 30.
LaTeX Font Info:    Font shape `T1/Merriwthr-OsF/m/up' will be
(Font)              scaled to size 5.24997pt on input line 30.
LaTeX Font Info:    Trying to load font information for U+eur on input
line 30.

(c:/texlive/2024/texmf-dist/tex/latex/amsfonts/ueur.fd
File: ueur.fd 2013/01/14 v3.01 Euler Roman
) (c:/texlive/2024/texmf-dist/tex/latex/microtype/mt-eur.cfg
File: mt-eur.cfg 2006/07/31 v1.1 microtype config. file: AMS Euler Roman
(RS)
)

LaTeX Font Warning: Font shape `OMS/cmsy/m/n' in size <7.5> not available
(Font)              size <7> substituted on input line 30.

LaTeX Font Info:    Trying to load font information for U+euf on input
line 30.

(c:/texlive/2024/texmf-dist/tex/latex/amsfonts/ueuf.fd
File: ueuf.fd 2013/01/14 v3.01 Euler Fraktur
) (c:/texlive/2024/texmf-dist/tex/latex/microtype/mt-euf.cfg
File: mt-euf.cfg 2006/07/03 v1.1 microtype config. file: AMS Euler
Fraktur (RS)

)
LaTeX Font Info:    Trying to load font information for U+eus on input
line 30.

(c:/texlive/2024/texmf-dist/tex/latex/amsfonts/ueus.fd
File: ueus.fd 2013/01/14 v3.01 Euler Script
) (c:/texlive/2024/texmf-dist/tex/latex/microtype/mt-eus.cfg
File: mt-eus.cfg 2006/07/28 v1.2 microtype config. file: AMS Euler Script
(RS)
)
LaTeX Font Info:    Trying to load font information for U+euex on input
line 30
.
(c:/texlive/2024/texmf-dist/tex/latex/amsfonts/ueuex.fd
File: ueuex.fd 2013/01/14 v3.01 Euler extra symbols
)
```

```
LaTeX Font Warning: Font shape `OML/cmm/m/it' in size <7.5> not available
(Font)              size <7> substituted on input line 30.

LaTeX Font Info:    Font shape `T1/Merriwthr-OsF/m/n' will be
(Font)              scaled to size 6.24973pt on input line 30.
LaTeX Font Info:    Font shape `T1/Merriwthr-OsF/m/n' will be
(Font)              scaled to size 5.24997pt on input line 30.
LaTeX Font Info:    Font shape `T1/Merriwthr-OsF/m/it' will be
(Font)              scaled to size 7.5pt on input line 30.
LaTeX Font Info:    Font shape `T1/Merriwthr-OsF/m/it' will be
(Font)              scaled to size 6.24973pt on input line 30.
LaTeX Font Info:    Font shape `T1/Merriwthr-OsF/m/it' will be
(Font)              scaled to size 5.24997pt on input line 30.
LaTeX Font Info:    Trying to load font information for U+msa on input
line 30.

(c:/texlive/2024/texmf-dist/tex/latex/amsfonts/umsa.fd
File: umsa.fd 2013/01/14 v3.01 AMS symbols A
) (c:/texlive/2024/texmf-dist/tex/latex/microtype/mt-msa.cfg
File: mt-msa.cfg 2006/02/04 v1.1 microtype config. file: AMS symbols (a)
(RS)
)
LaTeX Font Info:    Trying to load font information for U+msb on input
line 30.

(c:/texlive/2024/texmf-dist/tex/latex/amsfonts/umsb.fd
File: umsb.fd 2013/01/14 v3.01 AMS symbols B
) (c:/texlive/2024/texmf-dist/tex/latex/microtype/mt-msb.cfg
File: mt-msb.cfg 2005/06/01 v1.0 microtype config. file: AMS symbols (b)
(RS)
)
LaTeX Font Info:    Font shape `T1/Merriwthr-OsF/m/n' will be
(Font)              scaled to size 8.0pt on input line 30.
LaTeX Font Info:    Font shape `T1/Merriwthr-OsF/m/it' will be
(Font)              scaled to size 8.0pt on input line 30.
LaTeX Font Info:    Font shape `T1/Merriwthr-OsF/b/it' will be
(Font)              scaled to size 8.0pt on input line 30.
! Undefined control sequence.
<argument> \orgdiv
                   {School of Mathematics Sciences}, \orgname {Peking
Univer...
l.45 ...00871}, \state{Beijing}, \country{China}}}

The control sequence at the end of the top line
of your error message was never \def'ed. If you have
misspelled it (e.g., `\hobx'), type `I' and the correct
spelling (e.g., `I\hbox'). Otherwise just continue,
and I'll forget about whatever was undefined.

! Undefined control sequence.
<argument> ... of Mathematics Sciences}, \orgname
                                                  {Peking University},
\orga...
l.45 ...00871}, \state{Beijing}, \country{China}}}
```

```
The control sequence at the end of the top line
of your error message was never \def'ed. If you have
misspelled it (e.g., `\hobx'), type `I' and the correct
spelling (e.g., `I\hbox'). Otherwise just continue,
and I'll forget about whatever was undefined.

! Undefined control sequence.
<argument> ...me {Peking University}, \orgaddress
                                                  {\postcode {100871},
\stat...
l.45 ...00871}, \state{Beijing}, \country{China}}}

The control sequence at the end of the top line
of your error message was never \def'ed. If you have
misspelled it (e.g., `\hobx'), type `I' and the correct
spelling (e.g., `I\hbox'). Otherwise just continue,
and I'll forget about whatever was undefined.

! Undefined control sequence.
<argument> ...University}, \orgaddress {\postcode
                                                  {100871}, \state
{Beijing}...
l.45 ...00871}, \state{Beijing}, \country{China}}}

The control sequence at the end of the top line
of your error message was never \def'ed. If you have
misspelled it (e.g., `\hobx'), type `I' and the correct
spelling (e.g., `I\hbox'). Otherwise just continue,
and I'll forget about whatever was undefined.

! Undefined control sequence.
<argument> ...address {\postcode {100871}, \state
                                                  {Beijing}, \country
{China}}
l.45 ...00871}, \state{Beijing}, \country{China}}}

The control sequence at the end of the top line
of your error message was never \def'ed. If you have
misspelled it (e.g., `\hobx'), type `I' and the correct
spelling (e.g., `I\hbox'). Otherwise just continue,
and I'll forget about whatever was undefined.

! Undefined control sequence.
<argument> ...100871}, \state {Beijing}, \country
                                                  {China}}
l.45 ...00871}, \state{Beijing}, \country{China}}}

The control sequence at the end of the top line
of your error message was never \def'ed. If you have
misspelled it (e.g., `\hobx'), type `I' and the correct
spelling (e.g., `I\hbox'). Otherwise just continue,
and I'll forget about whatever was undefined.
```

```
! Undefined control sequence.
<argument> \orgdiv 
                   {School of Mathematics Sciences}, \orgname {Peking 
Univer...
l.45 ...00871}, \state{Beijing}, \country{China}}}

The control sequence at the end of the top line
of your error message was never \def'ed. If you have
misspelled it (e.g., `\hobx'), type `I' and the correct
spelling (e.g., `I\hbox'). Otherwise just continue,
and I'll forget about whatever was undefined.

! Undefined control sequence.
<argument> ... of Mathematics Sciences}, \orgname 
                                                  {Peking University}, 
\orga...
l.45 ...00871}, \state{Beijing}, \country{China}}}

The control sequence at the end of the top line
of your error message was never \def'ed. If you have
misspelled it (e.g., `\hobx'), type `I' and the correct
spelling (e.g., `I\hbox'). Otherwise just continue,
and I'll forget about whatever was undefined.

! Undefined control sequence.
<argument> ...me {Peking University}, \orgaddress 
                                                  {\postcode {100871}, 
\stat...
l.45 ...00871}, \state{Beijing}, \country{China}}}

The control sequence at the end of the top line
of your error message was never \def'ed. If you have
misspelled it (e.g., `\hobx'), type `I' and the correct
spelling (e.g., `I\hbox'). Otherwise just continue,
and I'll forget about whatever was undefined.

! Undefined control sequence.
<argument> ...University}, \orgaddress {\postcode 
                                                  {100871}, \state 
{Beijing}...
l.45 ...00871}, \state{Beijing}, \country{China}}}

The control sequence at the end of the top line
of your error message was never \def'ed. If you have
misspelled it (e.g., `\hobx'), type `I' and the correct
spelling (e.g., `I\hbox'). Otherwise just continue,
and I'll forget about whatever was undefined.

! Undefined control sequence.
<argument> ...address {\postcode {100871}, \state 
                                                  {Beijing}, \country 
{China}}
l.45 ...00871}, \state{Beijing}, \country{China}}}
```

```
The control sequence at the end of the top line
of your error message was never \def'ed. If you have
misspelled it (e.g., `\hobx'), type `I' and the correct
spelling (e.g., `I\hbox'). Otherwise just continue,
and I'll forget about whatever was undefined.

! Undefined control sequence.
<argument> ...100871}, \state {Beijing}, \country
                                                  {China}}
l.45 ...00871}, \state{Beijing}, \country{China}}}

The control sequence at the end of the top line
of your error message was never \def'ed. If you have
misspelled it (e.g., `\hobx'), type `I' and the correct
spelling (e.g., `I\hbox'). Otherwise just continue,
and I'll forget about whatever was undefined.

! Undefined control sequence.
<argument> \orgdiv
                   {Peking-Tsinghua Center for Life Sciences}, \orgname
{Pek...
l.46 ...00871}, \state{Beijing}, \country{China}}}

The control sequence at the end of the top line
of your error message was never \def'ed. If you have
misspelled it (e.g., `\hobx'), type `I' and the correct
spelling (e.g., `I\hbox'). Otherwise just continue,
and I'll forget about whatever was undefined.

! Undefined control sequence.
<argument> ...Center for Life Sciences}, \orgname
                                                  {Peking University},
\orga...
l.46 ...00871}, \state{Beijing}, \country{China}}}

The control sequence at the end of the top line
of your error message was never \def'ed. If you have
misspelled it (e.g., `\hobx'), type `I' and the correct
spelling (e.g., `I\hbox'). Otherwise just continue,
and I'll forget about whatever was undefined.

! Undefined control sequence.
<argument> ...me {Peking University}, \orgaddress
                                                  {\postcode {100871},
\stat...
l.46 ...00871}, \state{Beijing}, \country{China}}}

The control sequence at the end of the top line
of your error message was never \def'ed. If you have
misspelled it (e.g., `\hobx'), type `I' and the correct
spelling (e.g., `I\hbox'). Otherwise just continue,
and I'll forget about whatever was undefined.

! Undefined control sequence.
```

```
<argument> ...University}, \orgaddress {\postcode
                                                  {100871}, \state
{Beijing}...
l.46 ...00871}, \state{Beijing}, \country{China}}}

The control sequence at the end of the top line
of your error message was never \def'ed. If you have
misspelled it (e.g., `\hobx'), type `I' and the correct
spelling (e.g., `I\hbox'). Otherwise just continue,
and I'll forget about whatever was undefined.

! Undefined control sequence.
<argument> ...address {\postcode {100871}, \state
                                                  {Beijing}, \country
{China}}
l.46 ...00871}, \state{Beijing}, \country{China}}}

The control sequence at the end of the top line
of your error message was never \def'ed. If you have
misspelled it (e.g., `\hobx'), type `I' and the correct
spelling (e.g., `I\hbox'). Otherwise just continue,
and I'll forget about whatever was undefined.

! Undefined control sequence.
<argument> ...100871}, \state {Beijing}, \country
                                                  {China}}
l.46 ...00871}, \state{Beijing}, \country{China}}}

The control sequence at the end of the top line
of your error message was never \def'ed. If you have
misspelled it (e.g., `\hobx'), type `I' and the correct
spelling (e.g., `I\hbox'). Otherwise just continue,
and I'll forget about whatever was undefined.

! Undefined control sequence.
<argument> \orgdiv
                   {Peking-Tsinghua Center for Life Sciences}, \orgname
{Pek...
l.46 ...00871}, \state{Beijing}, \country{China}}}

The control sequence at the end of the top line
of your error message was never \def'ed. If you have
misspelled it (e.g., `\hobx'), type `I' and the correct
spelling (e.g., `I\hbox'). Otherwise just continue,
and I'll forget about whatever was undefined.

! Undefined control sequence.
<argument> ...Center for Life Sciences}, \orgname
                                                  {Peking University},
\orga...
l.46 ...00871}, \state{Beijing}, \country{China}}}

The control sequence at the end of the top line
of your error message was never \def'ed. If you have
```

```
misspelled it (e.g., `\hobx'), type `I' and the correct
spelling (e.g., `I\hbox'). Otherwise just continue,
and I'll forget about whatever was undefined.

! Undefined control sequence.
<argument> ...me {Peking University}, \orgaddress 
                                                  {\postcode {100871},
\stat...
l.46 ...00871}, \state{Beijing}, \country{China}}}

The control sequence at the end of the top line
of your error message was never \def'ed. If you have
misspelled it (e.g., `\hobx'), type `I' and the correct
spelling (e.g., `I\hbox'). Otherwise just continue,
and I'll forget about whatever was undefined.

! Undefined control sequence.
<argument> ...University}, \orgaddress {\postcode 
                                                  {100871}, \state
{Beijing}...
l.46 ...00871}, \state{Beijing}, \country{China}}}

The control sequence at the end of the top line
of your error message was never \def'ed. If you have
misspelled it (e.g., `\hobx'), type `I' and the correct
spelling (e.g., `I\hbox'). Otherwise just continue,
and I'll forget about whatever was undefined.

! Undefined control sequence.
<argument> ...address {\postcode {100871}, \state 
                                                  {Beijing}, \country
{China}}
l.46 ...00871}, \state{Beijing}, \country{China}}}

The control sequence at the end of the top line
of your error message was never \def'ed. If you have
misspelled it (e.g., `\hobx'), type `I' and the correct
spelling (e.g., `I\hbox'). Otherwise just continue,
and I'll forget about whatever was undefined.

! Undefined control sequence.
<argument> ...100871}, \state {Beijing}, \country 
                                                  {China}}
l.46 ...00871}, \state{Beijing}, \country{China}}}

The control sequence at the end of the top line
of your error message was never \def'ed. If you have
misspelled it (e.g., `\hobx'), type `I' and the correct
spelling (e.g., `I\hbox'). Otherwise just continue,
and I'll forget about whatever was undefined.

! Undefined control sequence.
<argument> \orgdiv
```

```
                  {Department of Computer Science}, \orgname {University
of...
l.47 ...024}, \state{Los Angeles}, \country{USA}}}

The control sequence at the end of the top line
of your error message was never \def'ed. If you have
misspelled it (e.g., `\hobx'), type `I' and the correct
spelling (e.g., `I\hbox'). Otherwise just continue,
and I'll forget about whatever was undefined.

! Undefined control sequence.
<argument> ...ment of Computer Science}, \orgname
                                                  {University of
California}...
l.47 ...024}, \state{Los Angeles}, \country{USA}}}

The control sequence at the end of the top line
of your error message was never \def'ed. If you have
misspelled it (e.g., `\hobx'), type `I' and the correct
spelling (e.g., `I\hbox'). Otherwise just continue,
and I'll forget about whatever was undefined.

! Undefined control sequence.
<argument> ...versity of California}, \orgaddress
                                                  {\postcode {90024},
\state...
l.47 ...024}, \state{Los Angeles}, \country{USA}}}

The control sequence at the end of the top line
of your error message was never \def'ed. If you have
misspelled it (e.g., `\hobx'), type `I' and the correct
spelling (e.g., `I\hbox'). Otherwise just continue,
and I'll forget about whatever was undefined.

! Undefined control sequence.
<argument> ...California}, \orgaddress {\postcode
                                                  {90024}, \state {Los
Angel...
l.47 ...024}, \state{Los Angeles}, \country{USA}}}

The control sequence at the end of the top line
of your error message was never \def'ed. If you have
misspelled it (e.g., `\hobx'), type `I' and the correct
spelling (e.g., `I\hbox'). Otherwise just continue,
and I'll forget about whatever was undefined.

! Undefined control sequence.
<argument> ...gaddress {\postcode {90024}, \state
                                                  {Los Angeles}, \country
{U...
l.47 ...024}, \state{Los Angeles}, \country{USA}}}

The control sequence at the end of the top line
of your error message was never \def'ed. If you have
```

```
misspelled it (e.g., `\hobx'), type `I' and the correct
spelling (e.g., `I\hbox'). Otherwise just continue,
and I'll forget about whatever was undefined.

! Undefined control sequence.
<argument> ...24}, \state {Los Angeles}, \country
                                                  {USA}}
l.47 ...024}, \state{Los Angeles}, \country{USA}}}

The control sequence at the end of the top line
of your error message was never \def'ed. If you have
misspelled it (e.g., `\hobx'), type `I' and the correct
spelling (e.g., `I\hbox'). Otherwise just continue,
and I'll forget about whatever was undefined.

! Undefined control sequence.
<argument> \orgdiv
                   {Department of Computer Science}, \orgname {University
of...
l.47 ...024}, \state{Los Angeles}, \country{USA}}}

The control sequence at the end of the top line
of your error message was never \def'ed. If you have
misspelled it (e.g., `\hobx'), type `I' and the correct
spelling (e.g., `I\hbox'). Otherwise just continue,
and I'll forget about whatever was undefined.

! Undefined control sequence.
<argument> ...ment of Computer Science}, \orgname
                                                  {University of
California}...
l.47 ...024}, \state{Los Angeles}, \country{USA}}}

The control sequence at the end of the top line
of your error message was never \def'ed. If you have
misspelled it (e.g., `\hobx'), type `I' and the correct
spelling (e.g., `I\hbox'). Otherwise just continue,
and I'll forget about whatever was undefined.

! Undefined control sequence.
<argument> ...versity of California}, \orgaddress
                                                  {\postcode {90024},
\state...
l.47 ...024}, \state{Los Angeles}, \country{USA}}}

The control sequence at the end of the top line
of your error message was never \def'ed. If you have
misspelled it (e.g., `\hobx'), type `I' and the correct
spelling (e.g., `I\hbox'). Otherwise just continue,
and I'll forget about whatever was undefined.

! Undefined control sequence.
<argument> ...California}, \orgaddress {\postcode
```

```
                                                  {90024}, \state {Los
Angel...
l.47 ...024}, \state{Los Angeles}, \country{USA}}}

The control sequence at the end of the top line
of your error message was never \def'ed. If you have
misspelled it (e.g., `\hobx'), type `I' and the correct
spelling (e.g., `I\hbox'). Otherwise just continue,
and I'll forget about whatever was undefined.

! Undefined control sequence.
<argument> ...gaddress {\postcode {90024}, \state
                                                  {Los Angeles}, \country
{U...
l.47 ...024}, \state{Los Angeles}, \country{USA}}}

The control sequence at the end of the top line
of your error message was never \def'ed. If you have
misspelled it (e.g., `\hobx'), type `I' and the correct
spelling (e.g., `I\hbox'). Otherwise just continue,
and I'll forget about whatever was undefined.

! Undefined control sequence.
<argument> ...24}, \state {Los Angeles}, \country
                                                  {USA}}
l.47 ...024}, \state{Los Angeles}, \country{USA}}}

The control sequence at the end of the top line
of your error message was never \def'ed. If you have
misspelled it (e.g., `\hobx'), type `I' and the correct
spelling (e.g., `I\hbox'). Otherwise just continue,
and I'll forget about whatever was undefined.

! Undefined control sequence.
<argument> \orgdiv
                   {Center for Quantitative Biology}, \orgname {Peking
Unive...
l.48 ...00871}, \state{Beijing}, \country{China}}}

The control sequence at the end of the top line
of your error message was never \def'ed. If you have
misspelled it (e.g., `\hobx'), type `I' and the correct
spelling (e.g., `I\hbox'). Otherwise just continue,
and I'll forget about whatever was undefined.

! Undefined control sequence.
<argument> ...for Quantitative Biology}, \orgname
                                                  {Peking University},
\orga...
l.48 ...00871}, \state{Beijing}, \country{China}}}

The control sequence at the end of the top line
of your error message was never \def'ed. If you have
misspelled it (e.g., `\hobx'), type `I' and the correct
```

```
spelling (e.g., `I\hbox'). Otherwise just continue,
and I'll forget about whatever was undefined.

! Undefined control sequence.
<argument> ...me {Peking University}, \orgaddress
                                                  {\postcode {100871},
\stat...
l.48 ...00871}, \state{Beijing}, \country{China}}}

The control sequence at the end of the top line
of your error message was never \def'ed. If you have
misspelled it (e.g., `\hobx'), type `I' and the correct
spelling (e.g., `I\hbox'). Otherwise just continue,
and I'll forget about whatever was undefined.

! Undefined control sequence.
<argument> ...University}, \orgaddress {\postcode
                                                  {100871}, \state
{Beijing}...
l.48 ...00871}, \state{Beijing}, \country{China}}}

The control sequence at the end of the top line
of your error message was never \def'ed. If you have
misspelled it (e.g., `\hobx'), type `I' and the correct
spelling (e.g., `I\hbox'). Otherwise just continue,
and I'll forget about whatever was undefined.

! Undefined control sequence.
<argument> ...address {\postcode {100871}, \state
                                                  {Beijing}, \country
{China}}
l.48 ...00871}, \state{Beijing}, \country{China}}}

The control sequence at the end of the top line
of your error message was never \def'ed. If you have
misspelled it (e.g., `\hobx'), type `I' and the correct
spelling (e.g., `I\hbox'). Otherwise just continue,
and I'll forget about whatever was undefined.

! Undefined control sequence.
<argument> ...100871}, \state {Beijing}, \country
                                                  {China}}
l.48 ...00871}, \state{Beijing}, \country{China}}}

The control sequence at the end of the top line
of your error message was never \def'ed. If you have
misspelled it (e.g., `\hobx'), type `I' and the correct
spelling (e.g., `I\hbox'). Otherwise just continue,
and I'll forget about whatever was undefined.

! Undefined control sequence.
<argument> \orgdiv
                   {Center for Quantitative Biology}, \orgname {Peking
Unive...
```

```
l.48 ...00871}, \state{Beijing}, \country{China}}}

The control sequence at the end of the top line
of your error message was never \def'ed. If you have
misspelled it (e.g., `\hobx'), type `I' and the correct
spelling (e.g., `I\hbox'). Otherwise just continue,
and I'll forget about whatever was undefined.

! Undefined control sequence.
<argument> ...for Quantitative Biology}, \orgname
                                                  {Peking University},
\orga...
l.48 ...00871}, \state{Beijing}, \country{China}}}

The control sequence at the end of the top line
of your error message was never \def'ed. If you have
misspelled it (e.g., `\hobx'), type `I' and the correct
spelling (e.g., `I\hbox'). Otherwise just continue,
and I'll forget about whatever was undefined.

! Undefined control sequence.
<argument> ...me {Peking University}, \orgaddress
                                                  {\postcode {100871},
\stat...
l.48 ...00871}, \state{Beijing}, \country{China}}}

The control sequence at the end of the top line
of your error message was never \def'ed. If you have
misspelled it (e.g., `\hobx'), type `I' and the correct
spelling (e.g., `I\hbox'). Otherwise just continue,
and I'll forget about whatever was undefined.

! Undefined control sequence.
<argument> ...University}, \orgaddress {\postcode
                                                  {100871}, \state
{Beijing}...
l.48 ...00871}, \state{Beijing}, \country{China}}}

The control sequence at the end of the top line
of your error message was never \def'ed. If you have
misspelled it (e.g., `\hobx'), type `I' and the correct
spelling (e.g., `I\hbox'). Otherwise just continue,
and I'll forget about whatever was undefined.

! Undefined control sequence.
<argument> ...address {\postcode {100871}, \state
                                                  {Beijing}, \country
{China}}
l.48 ...00871}, \state{Beijing}, \country{China}}}

The control sequence at the end of the top line
of your error message was never \def'ed. If you have
misspelled it (e.g., `\hobx'), type `I' and the correct
spelling (e.g., `I\hbox'). Otherwise just continue,
```

```
and I'll forget about whatever was undefined.

! Undefined control sequence.
<argument> ...100871}, \state {Beijing}, \country
                                                  {China}}
l.48 ...00871}, \state{Beijing}, \country{China}}}

The control sequence at the end of the top line
of your error message was never \def'ed. If you have
misspelled it (e.g., `\hobx'), type `I' and the correct
spelling (e.g., `I\hbox'). Otherwise just continue,
and I'll forget about whatever was undefined.

! Undefined control sequence.
<argument> \orgdiv
                   {Center for Statistical Science}, \orgname {Peking
Univer...
l.49 ...00871}, \state{Beijing}, \country{China}}}

The control sequence at the end of the top line
of your error message was never \def'ed. If you have
misspelled it (e.g., `\hobx'), type `I' and the correct
spelling (e.g., `I\hbox'). Otherwise just continue,
and I'll forget about whatever was undefined.

! Undefined control sequence.
<argument> ... for Statistical Science}, \orgname
                                                  {Peking University},
\orga...
l.49 ...00871}, \state{Beijing}, \country{China}}}

The control sequence at the end of the top line
of your error message was never \def'ed. If you have
misspelled it (e.g., `\hobx'), type `I' and the correct
spelling (e.g., `I\hbox'). Otherwise just continue,
and I'll forget about whatever was undefined.

! Undefined control sequence.
<argument> ...me {Peking University}, \orgaddress
                                                  {\postcode {100871},
\stat...
l.49 ...00871}, \state{Beijing}, \country{China}}}

The control sequence at the end of the top line
of your error message was never \def'ed. If you have
misspelled it (e.g., `\hobx'), type `I' and the correct
spelling (e.g., `I\hbox'). Otherwise just continue,
and I'll forget about whatever was undefined.

! Undefined control sequence.
<argument> ...University}, \orgaddress {\postcode
                                                  {100871}, \state
{Beijing}...
l.49 ...00871}, \state{Beijing}, \country{China}}}
```

```
The control sequence at the end of the top line
of your error message was never \def'ed. If you have
misspelled it (e.g., `\hobx'), type `I' and the correct
spelling (e.g., `I\hbox'). Otherwise just continue,
and I'll forget about whatever was undefined.

! Undefined control sequence.
<argument> ...address {\postcode {100871}, \state
                                                  {Beijing}, \country
{China}}
l.49 ...00871}, \state{Beijing}, \country{China}}}

The control sequence at the end of the top line
of your error message was never \def'ed. If you have
misspelled it (e.g., `\hobx'), type `I' and the correct
spelling (e.g., `I\hbox'). Otherwise just continue,
and I'll forget about whatever was undefined.

! Undefined control sequence.
<argument> ...100871}, \state {Beijing}, \country
                                                  {China}}
l.49 ...00871}, \state{Beijing}, \country{China}}}

The control sequence at the end of the top line
of your error message was never \def'ed. If you have
misspelled it (e.g., `\hobx'), type `I' and the correct
spelling (e.g., `I\hbox'). Otherwise just continue,
and I'll forget about whatever was undefined.

! Undefined control sequence.
<argument> \orgdiv
                   {Center for Statistical Science}, \orgname {Peking
Univer...
l.49 ...00871}, \state{Beijing}, \country{China}}}

The control sequence at the end of the top line
of your error message was never \def'ed. If you have
misspelled it (e.g., `\hobx'), type `I' and the correct
spelling (e.g., `I\hbox'). Otherwise just continue,
and I'll forget about whatever was undefined.

! Undefined control sequence.
<argument> ... for Statistical Science}, \orgname
                                                  {Peking University},
\orga...
l.49 ...00871}, \state{Beijing}, \country{China}}}

The control sequence at the end of the top line
of your error message was never \def'ed. If you have
misspelled it (e.g., `\hobx'), type `I' and the correct
spelling (e.g., `I\hbox'). Otherwise just continue,
and I'll forget about whatever was undefined.
```

```
! Undefined control sequence.
<argument> ...me {Peking University}, \orgaddress
                                                  {\postcode {100871},
\stat...
l.49 ...00871}, \state{Beijing}, \country{China}}}

The control sequence at the end of the top line
of your error message was never \def'ed. If you have
misspelled it (e.g., `\hobx'), type `I' and the correct
spelling (e.g., `I\hbox'). Otherwise just continue,
and I'll forget about whatever was undefined.

! Undefined control sequence.
<argument> ...University}, \orgaddress {\postcode
                                                  {100871}, \state
{Beijing}...
l.49 ...00871}, \state{Beijing}, \country{China}}}

The control sequence at the end of the top line
of your error message was never \def'ed. If you have
misspelled it (e.g., `\hobx'), type `I' and the correct
spelling (e.g., `I\hbox'). Otherwise just continue,
and I'll forget about whatever was undefined.

! Undefined control sequence.
<argument> ...address {\postcode {100871}, \state
                                                  {Beijing}, \country
{China}}
l.49 ...00871}, \state{Beijing}, \country{China}}}

The control sequence at the end of the top line
of your error message was never \def'ed. If you have
misspelled it (e.g., `\hobx'), type `I' and the correct
spelling (e.g., `I\hbox'). Otherwise just continue,
and I'll forget about whatever was undefined.

! Undefined control sequence.
<argument> ...100871}, \state {Beijing}, \country
                                                  {China}}
l.49 ...00871}, \state{Beijing}, \country{China}}}

The control sequence at the end of the top line
of your error message was never \def'ed. If you have
misspelled it (e.g., `\hobx'), type `I' and the correct
spelling (e.g., `I\hbox'). Otherwise just continue,
and I'll forget about whatever was undefined.

TextBlockOrigin set to 4pc+6.64pt x 4pc+6pt
<oup.pdf, id=104, 597.50829pt x 845.0471pt>
File: oup.pdf Graphic file (type pdf)
<use oup.pdf>
Package pdftex.def Info: oup.pdf  used on input line 78.
(pdftex.def)             Requested size: 34.31725pt x 48.538pt.
<gigascience-logo.pdf, id=105, 99.37125pt x 33.12375pt>
```

```
File: gigascience-logo.pdf Graphic file (type pdf)
<use gigascience-logo.pdf>
Package pdftex.def Info: gigascience-logo.pdf  used on input line 78.
(pdftex.def)             Requested size: 97.50749pt x 32.5pt.

Overfull \hbox (54.64pt too wide) in paragraph at lines 78--78
[][]
 []

LaTeX Font Info:    Font shape `T1/Merriwthr-OsF/m/n' will be
(Font)              scaled to size 14.0pt on input line 78.
LaTeX Font Info:    Font shape `T1/Merriwthr-OsF/m/n' will be
(Font)              scaled to size 8.99997pt on input line 78.
LaTeX Font Info:    Calculating math sizes for size <14> on input line
78.
LaTeX Font Info:    Font shape `T1/Merriwthr-OsF/m/up' will be
(Font)              scaled to size 14.0pt on input line 78.
LaTeX Font Info:    Font shape `T1/Merriwthr-OsF/m/up' will be
(Font)              scaled to size 11.66617pt on input line 78.
LaTeX Font Info:    Font shape `T1/Merriwthr-OsF/m/up' will be
(Font)              scaled to size 9.79996pt on input line 78.
LaTeX Font Info:    Font shape `T1/Merriwthr-OsF/m/n' will be
(Font)              scaled to size 11.66617pt on input line 78.
LaTeX Font Info:    Font shape `T1/Merriwthr-OsF/m/n' will be
(Font)              scaled to size 9.79996pt on input line 78.
LaTeX Font Info:    Font shape `T1/Merriwthr-OsF/m/it' will be
(Font)              scaled to size 14.0pt on input line 78.
LaTeX Font Info:    Font shape `T1/Merriwthr-OsF/m/it' will be
(Font)              scaled to size 11.66617pt on input line 78.
LaTeX Font Info:    Font shape `T1/Merriwthr-OsF/m/it' will be
(Font)              scaled to size 9.79996pt on input line 78.
LaTeX Font Info:    Font shape `T1/Merriwthr-OsF/b/n' will be
(Font)              scaled to size 18.0pt on input line 78.
LaTeX Font Info:    Font shape `T1/Merriwthr-OsF/m/n' will be
(Font)              scaled to size 13.0pt on input line 78.
LaTeX Font Info:    Calculating math sizes for size <13> on input line
78.
LaTeX Font Info:    Font shape `T1/Merriwthr-OsF/m/up' will be
(Font)              scaled to size 13.0pt on input line 78.
LaTeX Font Info:    Font shape `T1/Merriwthr-OsF/m/up' will be
(Font)              scaled to size 10.83287pt on input line 78.
LaTeX Font Info:    Font shape `T1/Merriwthr-OsF/m/up' will be
(Font)              scaled to size 9.09996pt on input line 78.

LaTeX Font Warning: Font shape `OMS/cmsy/m/n' in size <13> not available
(Font)              size <12> substituted on input line 78.


LaTeX Font Warning: Font shape `OML/cmm/m/it' in size <13> not available
(Font)              size <12> substituted on input line 78.

LaTeX Font Info:    Font shape `T1/Merriwthr-OsF/m/n' will be
(Font)              scaled to size 10.83287pt on input line 78.
LaTeX Font Info:    Font shape `T1/Merriwthr-OsF/m/n' will be
```

```
(Font)              scaled to size 9.09996pt on input line 78.
LaTeX Font Info:    Font shape `T1/Merriwthr-OsF/m/it' will be
(Font)              scaled to size 13.0pt on input line 78.
LaTeX Font Info:    Font shape `T1/Merriwthr-OsF/m/it' will be
(Font)              scaled to size 10.83287pt on input line 78.
LaTeX Font Info:    Font shape `T1/Merriwthr-OsF/m/it' will be
(Font)              scaled to size 9.09996pt on input line 78.
LaTeX Font Info:    Calculating math sizes for size <10.83287> on input
line 78
.
LaTeX Font Info:    Font shape `T1/Merriwthr-OsF/m/up' will be
(Font)              scaled to size 9.027pt on input line 78.
LaTeX Font Info:    Font shape `T1/Merriwthr-OsF/m/up' will be
(Font)              scaled to size 7.58296pt on input line 78.

LaTeX Font Warning: Font shape `OMS/cmsy/m/n' in size <7.58296> not
available
(Font)              size <8> substituted on input line 78.


LaTeX Font Warning: Font shape `OML/cmm/m/it' in size <7.58296> not
available
(Font)              size <8> substituted on input line 78.

LaTeX Font Info:    Font shape `T1/Merriwthr-OsF/m/n' will be
(Font)              scaled to size 9.027pt on input line 78.
LaTeX Font Info:    Font shape `T1/Merriwthr-OsF/m/n' will be
(Font)              scaled to size 7.58296pt on input line 78.
LaTeX Font Info:    Font shape `T1/Merriwthr-OsF/m/it' will be
(Font)              scaled to size 9.027pt on input line 78.
LaTeX Font Info:    Font shape `T1/Merriwthr-OsF/m/it' will be
(Font)              scaled to size 7.58296pt on input line 78.
LaTeX Font Info:    Font shape `T1/Merriwthr-OsF/m/n' will be
(Font)              scaled to size 9.0pt on input line 78.
LaTeX Font Info:    Font shape `T1/Merriwthr-OsF/m/up' will be
(Font)              scaled to size 9.0pt on input line 78.
LaTeX Font Info:    Font shape `T1/Merriwthr-OsF/m/up' will be
(Font)              scaled to size 7.0pt on input line 78.
LaTeX Font Info:    Font shape `T1/Merriwthr-OsF/m/up' will be
(Font)              scaled to size 5.0pt on input line 78.
LaTeX Font Info:    Font shape `T1/Merriwthr-OsF/m/n' will be
(Font)              scaled to size 7.0pt on input line 78.
LaTeX Font Info:    Font shape `T1/Merriwthr-OsF/m/n' will be
(Font)              scaled to size 5.0pt on input line 78.
LaTeX Font Info:    Font shape `T1/Merriwthr-OsF/m/it' will be
(Font)              scaled to size 9.0pt on input line 78.
LaTeX Font Info:    Font shape `T1/Merriwthr-OsF/m/it' will be
(Font)              scaled to size 7.0pt on input line 78.
LaTeX Font Info:    Font shape `T1/Merriwthr-OsF/m/it' will be
(Font)              scaled to size 5.0pt on input line 78.
LaTeX Font Info:    Font shape `T1/Merriwthr-OsF/m/n' will be
(Font)              scaled to size 6.5pt on input line 78.
LaTeX Font Info:    Calculating math sizes for size <6.5> on input line
78.
```

```
LaTeX Font Info:    Font shape `T1/Merriwthr-OsF/m/up' will be
(Font)              scaled to size 6.5pt on input line 78.
LaTeX Font Info:    Font shape `T1/Merriwthr-OsF/m/up' will be
(Font)              scaled to size 5.41643pt on input line 78.
LaTeX Font Info:    Font shape `T1/Merriwthr-OsF/m/up' will be
(Font)              scaled to size 4.54997pt on input line 78.

LaTeX Font Warning: Font shape `OMS/cmsy/m/n' in size <6.5> not available
(Font)              size <6> substituted on input line 78.


LaTeX Font Warning: Font shape `OMS/cmsy/m/n' in size <5.41643> not
available
(Font)              size <5> substituted on input line 78.


LaTeX Font Warning: Font shape `OMS/cmsy/m/n' in size <4.54997> not
available
(Font)              size <5> substituted on input line 78.


LaTeX Font Warning: Font shape `OML/cmm/m/it' in size <6.5> not available
(Font)              size <6> substituted on input line 78.


LaTeX Font Warning: Font shape `OML/cmm/m/it' in size <5.41643> not
available
(Font)              size <5> substituted on input line 78.


LaTeX Font Warning: Font shape `OML/cmm/m/it' in size <4.54997> not
available
(Font)              size <5> substituted on input line 78.

LaTeX Font Info:    Font shape `T1/Merriwthr-OsF/m/n' will be
(Font)              scaled to size 5.41643pt on input line 78.
LaTeX Font Info:    Font shape `T1/Merriwthr-OsF/m/n' will be
(Font)              scaled to size 4.54997pt on input line 78.
LaTeX Font Info:    Font shape `T1/Merriwthr-OsF/m/it' will be
(Font)              scaled to size 6.5pt on input line 78.
LaTeX Font Info:    Font shape `T1/Merriwthr-OsF/m/it' will be
(Font)              scaled to size 5.41643pt on input line 78.
LaTeX Font Info:    Font shape `T1/Merriwthr-OsF/m/it' will be
(Font)              scaled to size 4.54997pt on input line 78.
LaTeX Font Info:    Trying to load font information for TS1+Merriwthr-OsF
on in
put line 78.
(c:/texlive/2024/texmf-dist/tex/latex/merriweather/TS1Merriwthr-OsF.fd
File: TS1Merriwthr-OsF.fd 2020/08/30 (autoinst) Font definitions for
TS1/Merriw
thr-OsF.
)
LaTeX Font Info:    Font shape `TS1/Merriwthr-OsF/m/n' will be
(Font)              scaled to size 5.41643pt on input line 78.
```

```
Package microtype Info: Loading generic protrusion settings for font
family
(microtype)                `Merriwthr-OsF' (encoding: TS1).
(microtype)                For optimal results, create family-specific
settings.
(microtype)                See the microtype manual for details.

Overfull \hbox (54.64pt too wide) in paragraph at lines 78--78
[][][]
 []

LaTeX Font Info:    Font shape `T1/Merriwthr-OsF/b/n' will be
(Font)              scaled to size 10.0pt on input line 78.
LaTeX Font Info:    Font shape `T1/Merriwthr-OsF/b/n' will be
(Font)              scaled to size 8.0pt on input line 78.
LaTeX Font Info:    Font shape `T1/Merriwthr-OsF/m/up' will be
(Font)              scaled to size 8.0pt on input line 78.
LaTeX Font Info:    Font shape `T1/Merriwthr-OsF/m/up' will be
(Font)              scaled to size 6.0pt on input line 78.
LaTeX Font Info:    Font shape `T1/Merriwthr-OsF/m/n' will be
(Font)              scaled to size 6.0pt on input line 78.
LaTeX Font Info:    Font shape `T1/Merriwthr-OsF/m/it' will be
(Font)              scaled to size 6.0pt on input line 78.
LaTeX Font Info:    Trying to load font information for T1+lmtt on input
line 7
8.
(c:/texlive/2024/texmf-dist/tex/latex/lm/t1lmtt.fd
File: t1lmtt.fd 2015/05/01 v1.6.1 Font defs for Latin Modern
)
Package microtype Info: Loading generic protrusion settings for font
family
(microtype)                `lmtt' (encoding: T1).
(microtype)                For optimal results, create family-specific
settings.
(microtype)                See the microtype manual for details.

Overfull \hbox (54.64pt too wide) in paragraph at lines 78--78
[][][]
 []

Package mdframed Info: mdframed works in twoside mode on input line 81.
LaTeX Font Info:    Font shape `T1/Merriwthr-OsF/b/n' will be
(Font)              scaled to size 8.2pt on input line 81.
LaTeX Font Info:    Font shape `TS1/Merriwthr-OsF/m/n' will be
(Font)              scaled to size 7.5pt on input line 83.
Package mdframed Info: mdframed inside float
 mdframed uses option nobreak mdframed on input line 88.
Package mdframed Info: mdframed inside a box
 mdframed uses option nobreak mdframed on input line 88.
(./1_intro.tex
LaTeX Font Info:    Font shape `T1/Merriwthr-OsF/b/n' will be
(Font)              scaled to size 7.5pt on input line 8.
```

```
Package natbib Warning: Citation `eisenberg2000protein' on page 1
undefined on
input line 8.


Package natbib Warning: Citation `berman2000protein' on page 1 undefined
on inp
ut line 9.


Package natbib Warning: Citation `apweiler2004uniprot' on page 1
undefined on i
nput line 9.


Package natbib Warning: Citation `boutet2007uniprotkb' on page 1
undefined on i
nput line 9.


Package natbib Warning: Citation `uniprot2019uniprot' on page 1 undefined
on in
put line 9.


Package natbib Warning: Citation `zhou2019cafa' on page 1 undefined on
input li
ne 9.


Package natbib Warning: Citation `you2018golabeler' on page 1 undefined
on inpu
t line 9.


Package natbib Warning: Citation `kulmanov2018deepgo' on page 1 undefined
on in
put line 9.


Package natbib Warning: Citation `you2019netgo' on page 1 undefined on
input li
ne 9.


Package natbib Warning: Citation `gligorijevic2021structure' on page 1
undefine
d on input line 9.


Underfull \vbox (badness 10000) has occurred while \output is active []
```

```
Underfull \vbox (badness 10000) has occurred while \output is active []



LaTeX Font Info:    Font shape `T1/Merriwthr-OsF/m/n' will be
(Font)              scaled to size 7.8pt on input line 10.
LaTeX Font Info:    Font shape `T1/Merriwthr-OsF/b/n' will be
(Font)              scaled to size 7.8pt on input line 10.
[1{c:/texlive/2024/texmf-
var/fonts/map/pdftex/updmap/pdftex.map}{c:/texlive/202
4/texmf-
dist/fonts/enc/dvips/merriweather/merriwthr_posqbl.enc}{c:/texlive/2024
/texmf-
dist/fonts/enc/dvips/merriweather/merriwthr_owzwzj.enc}{c:/texlive/2024/
texmf-dist/fonts/enc/dvips/lm/lm-ec.enc}


 <./oup.pdf> <./gigascience-logo.pdf>]

Package natbib Warning: Citation `altschul1990basic' on page 2 undefined
on inp
ut line 10.


Package natbib Warning: Citation `das2015functional' on page 2 undefined
on inp
ut line 10.


Package natbib Warning: Citation `radivojac2013large' on page 2 undefined
on in
put line 10.


Package natbib Warning: Citation `altschul1990basic' on page 2 undefined
on inp
ut line 10.


Package natbib Warning: Citation `kulmanov2018deepgo' on page 2 undefined
on in
put line 11.


Package natbib Warning: Citation `fa2018predicting' on page 2 undefined
on inpu
t line 11.


Package natbib Warning: Citation `zhang2021prot2go' on page 2 undefined
on inpu
t line 11.
```

```
Package natbib Warning: Citation `cao2021tale' on page 2 undefined on
input lin
e 11.


Package natbib Warning: Citation `kulmanov2021deepgoplus' on page 2
undefined o
n input line 11.


Package natbib Warning: Citation `you2019netgo' on page 2 undefined on
input li
ne 11.


Package natbib Warning: Citation `sharan2007network' on page 2 undefined
on inp
ut line 11.


Package natbib Warning: Citation `mostafavi2008genemania' on page 2
undefined o
n input line 11.


Package natbib Warning: Citation `jiang2011predicting' on page 2
undefined on i
nput line 11.


Package natbib Warning: Citation `cho2016compact' on page 2 undefined on
input
line 11.


Package natbib Warning: Citation `you2021graph' on page 2 undefined on
input li
ne 11.


Package natbib Warning: Citation `gaudet2011phylogenetic' on page 2
undefined o
n input line 11.


Package natbib Warning: Citation `gligorijevic2021structure' on page 2
undefine
d on input line 11.


Package natbib Warning: Citation `konc2013structure' on page 2 undefined
on inp
ut line 11.
```

Package natbib Warning: Citation `lai2022accurate' on page 2 undefined on input
 line 11.

Package natbib Warning: Citation `ma2022enhancing' on page 2 undefined on input
 line 11.

Package natbib Warning: Citation `gu2023hierarchical' on page 2 undefined on in
put line 11.

Package natbib Warning: Citation `verspoor2014roles' on page 2 undefined on inp
ut line 11.

Package natbib Warning: Citation `yao2021netgo' on page 2 undefined on input li
ne 11.

Package natbib Warning: Citation `cao2021tale' on page 2 undefined on input lin
e 11.

Package natbib Warning: Citation `kulmanov2021deepgoplus' on page 2 undefined o
n input line 11.

Package natbib Warning: Citation `gligorijevic2021structure' on page 2 undefine
d on input line 12.

Package natbib Warning: Citation `zhou2022tasser' on page 2 undefined on input
line 12.

Package natbib Warning: Citation `varadi2022alphafold' on page 2 undefined on i
nput line 12.

Package natbib Warning: Citation `baek2021Rose' on page 2 undefined on input li

```
ne 12.


Package natbib Warning: Citation `lin2023evolutionary' on page 2
undefined on i
nput line 12.


Package natbib Warning: Citation `ma2022enhancing' on page 2 undefined on
input
 line 12.


Package natbib Warning: Citation `wang2023netgo' on page 2 undefined on
input l
ine 12.


Package natbib Warning: Citation `yao2021netgo' on page 2 undefined on
input li
ne 12.


Package natbib Warning: Citation `gu2023hierarchical' on page 2 undefined
on in
put line 12.


Package natbib Warning: Citation `zhang2018metago' on page 2 undefined on
input
 line 12.


Underfull \vbox (badness 3635) has occurred while \output is active []



LaTeX Font Info:    Font shape `T1/Merriwthr-OsF/m/up' will be
(Font)              scaled to size 7.5pt on input line 21.

Package natbib Warning: Citation `altschul1990basic' on page 2 undefined
on inp
ut line 23.


Package natbib Warning: Citation `kulmanov2021deepgoplus' on page 2
undefined o
n input line 23.


Package natbib Warning: Citation `cao2021tale' on page 2 undefined on
input lin
e 23.
```

```
Package natbib Warning: Citation `gligorijevic2021structure' on page 2
undefine
d on input line 23.


Package natbib Warning: Citation `ma2022enhancing' on page 2 undefined on
input
 line 23.


Package natbib Warning: Citation `gu2023hierarchical' on page 2 undefined
on in
put line 23.

) (./2_method.tex
<figure1.png, id=174, 3019.28pt x 855.195pt>
File: figure1.png Graphic file (type png)
<use figure1.png>
Package pdftex.def Info: figure1.png  used on input line 4.
(pdftex.def)             Requested size: 488.22787pt x 138.28246pt.
LaTeX Font Info:    Font shape `T1/Merriwthr-OsF/b/n' will be
(Font)              scaled to size 6.0pt on input line 5.
LaTeX Font Info:    Font shape `T1/Merriwthr-OsF/b/n' will be
(Font)              scaled to size 8.5pt on input line 10.

Package natbib Warning: Citation `gu2023hierarchical' on page 2 undefined
on in
put line 11.



LaTeX Font Info:    Font shape `T1/Merriwthr-OsF/m/it' will be
(Font)              scaled to size 7.8pt on input line 13.
[2]

Package natbib Warning: Citation `rives2021biological' on page 3
undefined on i
nput line 13.


Package natbib Warning: Citation `xu2019powerful' on page 3 undefined on
input
line 24.


Package natbib Warning: Citation `gilmer2020message' on page 3 undefined
on inp
ut line 24.


Underfull \vbox (badness 2762) has occurred while \output is active []
```

```
Package natbib Warning: Citation `baek2021accurate' on page 3 undefined on inpu
t line 30.


Package natbib Warning: Citation `vaswani2017attention' on page 3 undefined on
input line 35.



[3 <./figure1.png>]

Package natbib Warning: Citation `gligorijevic2021structure' on page 4 undefine
d on input line 50.


Package natbib Warning: Citation `long2018conditional' on page 4 undefined on i
nput line 51.




Package natbib Warning: Citation `cao2021tale' on page 4 undefined on input lin
e 77.


Package natbib Warning: Citation `kingma2014adam' on page 4 undefined on input
line 87.

) (./3_experiment.tex

Package natbib Warning: Citation `gligorijevic2021structure' on page 4 undefine
d on input line 2.


Package natbib Warning: Citation `berman2000protein' on page 4 undefined on inp
ut line 2.


Package natbib Warning: Citation `dana2019sifts' on page 4 undefined on input l
ine 2.
```

```
Package natbib Warning: Citation `mirdita2021fast' on page 4 undefined on
input
 line 2.


Package natbib Warning: Citation `ashburner2000gene' on page 4 undefined
on inp
ut line 3.


Package natbib Warning: Citation `waterhouse2018swiss' on page 4
undefined on i
nput line 4.


Package natbib Warning: Citation `gu2023hierarchical' on page 4 undefined
on in
put line 4.


Package natbib Warning: Citation `varadi2022alphafold' on page 4
undefined on i
nput line 4.

LaTeX Font Info:    Font shape `T1/Merriwthr-OsF/b/n' will be
(Font)              scaled to size 7.0pt on input line 8.


[4]

Package natbib Warning: Citation `radivojac2013large' on page 5 undefined
on in
put line 30.


Package natbib Warning: Citation `kulmanov2018deepgo' on page 5 undefined
on in
put line 46.




Overfull \hbox (9.05498pt too wide) in paragraph at lines 69--83
 [][]
 []


! LaTeX Error: Something's wrong--perhaps a missing \item.

See the LaTeX manual or LaTeX Companion for explanation.
Type  H <return>  for immediate help.
 ...

l.109 \end{tablenotes}
```

```
Try typing  <return>  to proceed.
If that doesn't work, type  X <return>  to quit.


Underfull \vbox (badness 10000) has occurred while \output is active []



[5]

pdfTeX warning: pdflatex.exe (file ./figure6.pdf): PDF inclusion: found
PDF ver
sion <1.7>, but at most version <1.5> allowed
<figure6.pdf, id=225, 963.65018pt x 341.42555pt>
File: figure6.pdf Graphic file (type pdf)
<use figure6.pdf>
Package pdftex.def Info: figure6.pdf  used on input line 114.
(pdftex.def)             Requested size: 439.4021pt x 155.68228pt.


pdfTeX warning: pdflatex.exe (file ./figure2.pdf): PDF inclusion: found
PDF ver
sion <1.7>, but at most version <1.5> allowed
<figure2.pdf, id=226, 963.65018pt x 369.88188pt>
File: figure2.pdf Graphic file (type pdf)
<use figure2.pdf>
Package pdftex.def Info: figure2.pdf  used on input line 121.
(pdftex.def)             Requested size: 390.58379pt x 149.91986pt.

Underfull \vbox (badness 10000) has occurred while \output is active []




[6 <./figure6.pdf>]
Package epstopdf Info: Source file: <figure3.eps>
(epstopdf)                    date: 2024-10-18 09:17:57
(epstopdf)                    size: 42274 bytes
(epstopdf)             Output file: <figure3-eps-converted-to.pdf>
(epstopdf)                    date: 2024-10-18 09:18:18
(epstopdf)                    size: 16083 bytes
(epstopdf)             Command: <repstopdf --outfile=figure3-eps-
converted-to.p
df figure3.eps>
(epstopdf)             \includegraphics on input line 136.
Package epstopdf Info: Output file is already uptodate.
<figure3-eps-converted-to.pdf, id=249, 2890.8pt x 867.24pt>
File: figure3-eps-converted-to.pdf Graphic file (type pdf)
<use figure3-eps-converted-to.pdf>
Package pdftex.def Info: figure3-eps-converted-to.pdf  used on input line
136.
(pdftex.def)             Requested size: 463.81499pt x 139.14499pt.
```

```
! LaTeX Error: Something's wrong--perhaps a missing \item.

See the LaTeX manual or LaTeX Companion for explanation.
Type  H <return>  for immediate help.
 ...

l.164 \end{tablenotes}

Try typing  <return>  to proceed.
If that doesn't work, type  X <return>  to quit.




Package natbib Warning: Citation `liu2024plmsearch' on page 7 undefined
on inpu
t line 170.


Package natbib Warning: Citation `zhang2005tm' on page 7 undefined on
input lin
e 170.


Package natbib Warning: Citation `van2022foldseek' on page 7 undefined on
input
 line 170.

Package epstopdf Info: Source file: <figure4.eps>
(epstopdf)                    date: 2024-10-18 09:17:57
(epstopdf)                    size: 27632 bytes
(epstopdf)             Output file: <figure4-eps-converted-to.pdf>
(epstopdf)                    date: 2024-10-18 09:18:21
(epstopdf)                    size: 14395 bytes
(epstopdf)             Command: <repstopdf --outfile=figure4-eps-
converted-to.p
df figure4.eps>
(epstopdf)             \includegraphics on input line 176.
Package epstopdf Info: Output file is already uptodate.
<figure4-eps-converted-to.pdf, id=250, 1806.75pt x 722.7pt>
File: figure4-eps-converted-to.pdf Graphic file (type pdf)
<use figure4-eps-converted-to.pdf>
Package pdftex.def Info: figure4-eps-converted-to.pdf  used on input line
176.
(pdftex.def)             Requested size: 235.11394pt x 94.04251pt.

Package natbib Warning: Citation `boutet2007uniprotkb' on page 7
undefined on i
nput line 180.


Package natbib Warning: Citation `boutet2016uniprotkb' on page 7
undefined on i
```

```
nput line 180.



[7 <./figure2.pdf> <./figure3-eps-converted-to.pdf>]

pdfTeX warning: pdflatex.exe (file ./figure5.pdf): PDF inclusion: found
PDF ver
sion <1.7>, but at most version <1.5> allowed
<figure5.pdf, id=320, 569.0058pt x 426.69414pt>
File: figure5.pdf Graphic file (type pdf)
<use figure5.pdf>
Package pdftex.def Info: figure5.pdf  used on input line 184.
(pdftex.def)             Requested size: 390.58379pt x 292.89574pt.

Package natbib Warning: Citation `selvaraju2020grad' on page 8 undefined
on inp
ut line 188.


Underfull \vbox (badness 4156) has occurred while \output is active []




Package natbib Warning: Citation `yang2012biolip' on page 8 undefined on
input
line 200.


! LaTeX Error: Something's wrong--perhaps a missing \item.

See the LaTeX manual or LaTeX Companion for explanation.
Type  H <return>  for immediate help.
 ...

l.232 \end{tablenotes}

Try typing  <return>  to proceed.
If that doesn't work, type  X <return>  to quit.

) (./4_discussion.tex

[8 <./figure4-eps-converted-to.pdf>]
Underfull \vbox (badness 4254) has occurred while \output is active []



)
Underfull \hbox (badness 2680) in paragraph at lines 94--105
\T1/Merriwthr-OsF/m/up/7.5 (+20) Supplementary Fig-ure S4. Con-tri-bu-
tion scor
e com-puted by
 []
```

```
Underfull \vbox (badness 1259) has occurred while \output is active []


[9 <./figure5.pdf>]

Package natbib Warning: Citation `gligorijevic2021structure' on page 10
undefin
ed on input line 106.


Package natbib Warning: Citation `steinegger2018clustering' on page 10
undefine
d on input line 107.


Package natbib Warning: Citation `fu2024learning' on page 10 undefined on
input
 line 108.


Package natbib Warning: Citation `fu2024learning' on page 10 undefined on
input
 line 108.

No file main.bbl.

Package natbib Warning: There were undefined citations.


[10

]
enddocument/afterlastpage: lastpage setting LastPage.
(./main.aux)
 ***********
LaTeX2e <2024-06-01> patch level 2
L3 programming layer <2020/03/25>
 ***********

LaTeX Font Warning: Size substitutions with differences
(Font)              up to 1.0pt have occurred.


LaTeX Font Warning: Some font shapes were not available, defaults
substituted.

Package rerunfilecheck Info: File `main.out' has not changed.
```

```
(rerunfilecheck)             Checksum:
DDA843EBFB255F62BB6CD4C57C511500;4927.
 )
Here is how much of TeX's memory you used:
 36167 strings out of 473583
 726112 string characters out of 5732343
 1989908 words of memory out of 5000000
 57623 multiletter control sequences out of 15000+600000
 1996778 words of font info for 697 fonts, out of 8000000 for 9000
 1141 hyphenation exceptions out of 8191
 123i,13n,131p,2313b,974s stack positions out of
10000i,1000n,20000p,200000b,200000s
<c:/texlive/2024/texmf-dist/fonts/type1/sorkin/merriweather/Merriwthr-
Bold.pf
b><c:/texlive/2024/texmf-dist/fonts/type1/sorkin/merriweather/Merriwthr-
BoldIta
lic.pfb><c:/texlive/2024/texmf-
dist/fonts/type1/sorkin/merriweather/Merriwthr-I
talic.pfb><c:/texlive/2024/texmf-
dist/fonts/type1/sorkin/merriweather/Merriwthr
-Regular.pfb><c:/texlive/2024/texmf-
dist/fonts/type1/public/amsfonts/cmextra/cm
ex8.pfb><c:/texlive/2024/texmf-
dist/fonts/type1/public/amsfonts/cm/cmsy10.pfb><
c:/texlive/2024/texmf-
dist/fonts/type1/public/amsfonts/cm/cmsy6.pfb><c:/texlive
/2024/texmf-
dist/fonts/type1/public/amsfonts/cm/cmsy7.pfb><c:/texlive/2024/texm
f-
dist/fonts/type1/public/amsfonts/euler/euex7.pfb><c:/texlive/2024/texmf-
dist/
fonts/type1/public/amsfonts/euler/euex8.pfb><c:/texlive/2024/texmf-
dist/fonts/t
ype1/public/amsfonts/euler/eufm7.pfb><c:/texlive/2024/texmf-
dist/fonts/type1/pu
blic/amsfonts/euler/eurm5.pfb><c:/texlive/2024/texmf-
dist/fonts/type1/public/am
sfonts/euler/eurm7.pfb><c:/texlive/2024/texmf-
dist/fonts/type1/public/lm/lmtt8.
pfb><c:/texlive/2024/texmf-
dist/fonts/type1/public/amsfonts/symbols/msam7.pfb>
Output written on main.pdf (10 pages, 1451947 bytes).
PDF statistics:
 484 PDF objects out of 1000 (max. 8388607)
 320 compressed objects within 4 object streams
 69 named destinations out of 1000 (max. 500000)
 220405 words of extra memory for PDF output out of 221844 (max.
10000000)
```

Click here to access/download;LaTeX - Main Document (TeX file);supplementary_information.tex

```
This is pdfTeX, Version 3.141592653-2.6-1.40.26 (TeX Live 2024)
(preloaded format=pdflatex 2024.8.2)  18 OCT 2024 09:18
entering extended mode
 restricted \write18 enabled.
 %&-line parsing enabled.
**supplementary_information.tex
(./supplementary_information.tex
LaTeX2e <2024-06-01> patch level 2
L3 programming layer <2024-05-27>
(c:/texlive/2024/texmf-dist/tex/latex/base/article.cls
Document Class: article 2024/02/08 v1.4n Standard LaTeX document class
(c:/texlive/2024/texmf-dist/tex/latex/base/size10.clo
File: size10.clo 2024/02/08 v1.4n Standard LaTeX file (size option)
)
\c@part=\count194
\c@section=\count195
\c@subsection=\count196
\c@subsubsection=\count197
\c@paragraph=\count198
\c@subparagraph=\count199
\c@figure=\count266
\c@table=\count267
\abovecaptionskip=\skip49
\belowcaptionskip=\skip50
\bibindent=\dimen141
) (./neurips_2019.sty
Package: neurips_2019 2019/03/13 NeurIPS 2019 submission/camera-ready
style fil
e
(c:/texlive/2024/texmf-dist/tex/latex/natbib/natbib.sty
Package: natbib 2010/09/13 8.31b (PWD, AO)
\bibhang=\skip51
\bibsep=\skip52
LaTeX Info: Redefining \cite on input line 694.
\c@NAT@ctr=\count268
) (c:/texlive/2024/texmf-dist/tex/latex/geometry/geometry.sty
Package: geometry 2020/01/02 v5.9 Page Geometry
(c:/texlive/2024/texmf-dist/tex/latex/graphics/keyval.sty
Package: keyval 2022/05/29 v1.15 key=value parser (DPC)
\KV@toks@=\toks17
) (c:/texlive/2024/texmf-dist/tex/generic/iftex/ifvtex.sty
Package: ifvtex 2019/10/25 v1.7 ifvtex legacy package. Use iftex instead.
(c:/texlive/2024/texmf-dist/tex/generic/iftex/iftex.sty
Package: iftex 2022/02/03 v1.0f TeX engine tests
))
\Gm@cnth=\count269
\Gm@cntv=\count270
\c@Gm@tempcnt=\count271
\Gm@bindingoffset=\dimen142
\Gm@wd@mp=\dimen143
\Gm@odd@mp=\dimen144
\Gm@even@mp=\dimen145
\Gm@layoutwidth=\dimen146
\Gm@layoutheight=\dimen147
```

```
\Gm@layouthoffset=\dimen148
\Gm@layoutvoffset=\dimen149
\Gm@dimlist=\toks18
)
\@neuripsabovecaptionskip=\skip53
\@neuripsbelowcaptionskip=\skip54
) (c:/texlive/2024/texmf-dist/tex/latex/graphics/rotating.sty
Package: rotating 2016/08/11 v2.16d rotated objects in LaTeX
(c:/texlive/2024/texmf-dist/tex/latex/graphics/graphicx.sty
Package: graphicx 2021/09/16 v1.2d Enhanced LaTeX Graphics (DPC,SPQR)
(c:/texlive/2024/texmf-dist/tex/latex/graphics/graphics.sty
Package: graphics 2024/05/23 v1.4g Standard LaTeX Graphics (DPC,SPQR)
(c:/texlive/2024/texmf-dist/tex/latex/graphics/trig.sty
Package: trig 2023/12/02 v1.11 sin cos tan (DPC)
) (c:/texlive/2024/texmf-dist/tex/latex/graphics-cfg/graphics.cfg
File: graphics.cfg 2016/06/04 v1.11 sample graphics configuration
)
Package graphics Info: Driver file: pdftex.def on input line 106.
(c:/texlive/2024/texmf-dist/tex/latex/graphics-def/pdftex.def
File: pdftex.def 2024/04/13 v1.2c Graphics/color driver for pdftex
))
\Gin@req@height=\dimen150
\Gin@req@width=\dimen151
) (c:/texlive/2024/texmf-dist/tex/latex/base/ifthen.sty
Package: ifthen 2024/03/16 v1.1e Standard LaTeX ifthen package (DPC)
)
\c@r@tfl@t=\count272
\rotFPtop=\skip55
\rotFPbot=\skip56
\rot@float@box=\box52
\rot@mess@toks=\toks19
) (c:/texlive/2024/texmf-dist/tex/latex/tools/longtable.sty
Package: longtable 2024-04-26 v4.20 Multi-page Table package (DPC)
\LTleft=\skip57
\LTright=\skip58
\LTpre=\skip59
\LTpost=\skip60
\LTchunksize=\count273
\LTcapwidth=\dimen152
\LT@head=\box53
\LT@firsthead=\box54
\LT@foot=\box55
\LT@lastfoot=\box56
\LT@gbox=\box57
\LT@cols=\count274
\LT@rows=\count275
\c@LT@tables=\count276
\c@LT@chunks=\count277
\LT@p@ftn=\toks20
) (c:/texlive/2024/texmf-dist/tex/latex/booktabs/booktabs.sty
Package: booktabs 2020/01/12 v1.61803398 Publication quality tables
\heavyrulewidth=\dimen153
\lightrulewidth=\dimen154
\cmidrulewidth=\dimen155
```

```
\belowrulesep=\dimen156
\belowbottomsep=\dimen157
\aboverulesep=\dimen158
\abovetopsep=\dimen159
\cmidrulesep=\dimen160
\cmidrulekern=\dimen161
\defaultaddspace=\dimen162
\@cmidla=\count278
\@cmidlb=\count279
\@aboverulesep=\dimen163
\@belowrulesep=\dimen164
\@thisruleclass=\count280
\@lastruleclass=\count281
\@thisrulewidth=\dimen165
) (c:/texlive/2024/texmf-dist/tex/latex/graphics/lscape.sty
Package: lscape 2020/05/28 v3.02 Landscape Pages (DPC)
) (c:/texlive/2024/texmf-dist/tex/latex/tabu/tabu.sty
Package: tabu 2019/01/11 v2.9 - flexible LaTeX tabulars (FC+tabu-fixed)
(c:/texlive/2024/texmf-dist/tex/latex/tools/array.sty
Package: array 2024/06/14 v2.6d Tabular extension package (FMi)
\col@sep=\dimen166
\ar@mcellbox=\box58
\extrarowheight=\dimen167
\NC@list=\toks21
\extratabsurround=\skip61
\backup@length=\skip62
\ar@cellbox=\box59
) (c:/texlive/2024/texmf-dist/tex/latex/varwidth/varwidth.sty
Package: varwidth 2009/03/30 ver 0.92;  Variable-width minipages
\@vwid@box=\box60
\sift@deathcycles=\count282
\@vwid@loff=\dimen168
\@vwid@roff=\dimen169
)
\c@taburow=\count283
\tabu@nbcols=\count284
\tabu@cnt=\count285
\tabu@Xcol=\count286
\tabu@alloc=\count287
\tabu@nested=\count288
\tabu@target=\dimen170
\tabu@spreadtarget=\dimen171
\tabu@naturalX=\dimen172
\tabucolX=\dimen173
\tabu@Xsum=\dimen174
\extrarowdepth=\dimen175
\abovetabulinesep=\dimen176
\belowtabulinesep=\dimen177
\tabustrutrule=\dimen178
\tabu@thebody=\toks22
\tabu@footnotes=\toks23
\tabu@box=\box61
\tabu@arstrutbox=\box62
\tabu@hleads=\box63
```

```
\tabu@vleads=\box64
\tabu@cellskip=\skip63
) (c:/texlive/2024/texmf-dist/tex/latex/amscls/amsthm.sty
Package: amsthm 2020/05/29 v2.20.6
\thm@style=\toks24
\thm@bodyfont=\toks25
\thm@headfont=\toks26
\thm@notefont=\toks27
\thm@headpunct=\toks28
\thm@preskip=\skip64
\thm@postskip=\skip65
\thm@headsep=\skip66
\dth@everypar=\toks29
) (c:/texlive/2024/texmf-dist/tex/latex/amsmath/amsmath.sty
Package: amsmath 2024/05/23 v2.17q AMS math features
\@mathmargin=\skip67
For additional information on amsmath, use the `?' option.
(c:/texlive/2024/texmf-dist/tex/latex/amsmath/amstext.sty
Package: amstext 2021/08/26 v2.01 AMS text
(c:/texlive/2024/texmf-dist/tex/latex/amsmath/amsgen.sty
File: amsgen.sty 1999/11/30 v2.0 generic functions
\@emptytoks=\toks30
\ex@=\dimen179
)) (c:/texlive/2024/texmf-dist/tex/latex/amsmath/amsbsy.sty
Package: amsbsy 1999/11/29 v1.2d Bold Symbols
\pmbraise@=\dimen180
) (c:/texlive/2024/texmf-dist/tex/latex/amsmath/amsopn.sty
Package: amsopn 2022/04/08 v2.04 operator names
)
\inf@bad=\count289
LaTeX Info: Redefining \frac on input line 233.
\uproot@=\count290
\leftroot@=\count291
LaTeX Info: Redefining \overline on input line 398.
LaTeX Info: Redefining \colon on input line 409.
\classnum@=\count292
\DOTSCASE@=\count293
LaTeX Info: Redefining \ldots on input line 495.
LaTeX Info: Redefining \dots on input line 498.
LaTeX Info: Redefining \cdots on input line 619.
\Mathstrutbox@=\box65
\strutbox@=\box66
LaTeX Info: Redefining \big on input line 721.
LaTeX Info: Redefining \Big on input line 722.
LaTeX Info: Redefining \bigg on input line 723.
LaTeX Info: Redefining \Bigg on input line 724.
\big@size=\dimen181
LaTeX Font Info:    Redeclaring font encoding OML on input line 742.
LaTeX Font Info:    Redeclaring font encoding OMS on input line 743.
\macc@depth=\count294
LaTeX Info: Redefining \bmod on input line 904.
LaTeX Info: Redefining \pmod on input line 909.
LaTeX Info: Redefining \smash on input line 939.
LaTeX Info: Redefining \relbar on input line 969.
```

```
LaTeX Info: Redefining \Relbar on input line 970.
\c@MaxMatrixCols=\count295
\dotsspace@=\muskip17
\c@parentequation=\count296
\dspbrk@lvl=\count297
\tag@help=\toks31
\row@=\count298
\column@=\count299
\maxfields@=\count300
\andhelp@=\toks32
\eqnshift@=\dimen182
\alignsep@=\dimen183
\tagshift@=\dimen184
\tagwidth@=\dimen185
\totwidth@=\dimen186
\lineht@=\dimen187
\@envbody=\toks33
\multlinegap=\skip68
\multlinetaggap=\skip69
\mathdisplay@stack=\toks34
LaTeX Info: Redefining \[ on input line 2953.
LaTeX Info: Redefining \] on input line 2954.
) (c:/texlive/2024/texmf-dist/tex/latex/amsfonts/amssymb.sty
Package: amssymb 2013/01/14 v3.01 AMS font symbols
(c:/texlive/2024/texmf-dist/tex/latex/amsfonts/amsfonts.sty
Package: amsfonts 2013/01/14 v3.01 Basic AMSFonts support
\symAMSa=\mathgroup4
\symAMSb=\mathgroup5
LaTeX Font Info:    Redeclaring math symbol \hbar on input line 98.
LaTeX Font Info:    Overwriting math alphabet `\mathfrak' in version
`bold'
(Font)                  U/euf/m/n --> U/euf/b/n on input line 106.
)) (c:/texlive/2024/texmf-dist/tex/latex/jknapltx/mathrsfs.sty
Package: mathrsfs 1996/01/01 Math RSFS package v1.0 (jk)
\symrsfs=\mathgroup6
) (c:/texlive/2024/texmf-dist/tex/latex/multirow/multirow.sty
Package: multirow 2021/03/15 v2.8 Span multiple rows of a table
\multirow@colwidth=\skip70
\multirow@cntb=\count301
\multirow@dima=\skip71
\bigstrutjot=\dimen188
) (c:/texlive/2024/texmf-dist/tex/latex/float/float.sty
Package: float 2001/11/08 v1.3d Float enhancements (AL)
\c@float@type=\count302
\float@exts=\toks35
\float@box=\box67
\@float@everytoks=\toks36
\@floatcapt=\box68
) (c:/texlive/2024/texmf-dist/tex/latex/caption/caption.sty
Package: caption 2023/08/05 v3.6o Customizing captions (AR)
(c:/texlive/2024/texmf-dist/tex/latex/caption/caption3.sty
Package: caption3 2023/07/31 v2.4d caption3 kernel (AR)
\caption@tempdima=\dimen189
\captionmargin=\dimen190
```

```
\caption@leftmargin=\dimen191
\caption@rightmargin=\dimen192
\caption@width=\dimen193
\caption@indent=\dimen194
\caption@parindent=\dimen195
\caption@hangindent=\dimen196
Package caption Info: Standard document class detected.
)
\c@caption@flags=\count303
\c@continuedfloat=\count304
Package caption Info: float package is loaded.
Package caption Info: longtable package is loaded.
(c:/texlive/2024/texmf-dist/tex/latex/caption/ltcaption.sty
Package: ltcaption 2021/01/08 v1.4c longtable captions (AR)
)
Package caption Info: rotating package is loaded.
) (c:/texlive/2024/texmf-dist/tex/latex/threeparttable/threeparttable.sty
Package: threeparttable 2003/06/13  v 3.0
\@tempboxb=\box69
) (c:/texlive/2024/texmf-dist/tex/latex/xcolor/xcolor.sty
Package: xcolor 2023/11/15 v3.01 LaTeX color extensions (UK)
(c:/texlive/2024/texmf-dist/tex/latex/graphics-cfg/color.cfg
File: color.cfg 2016/01/02 v1.6 sample color configuration
)
Package xcolor Info: Driver file: pdftex.def on input line 274.
(c:/texlive/2024/texmf-dist/tex/latex/graphics/mathcolor.ltx)
Package xcolor Info: Model `cmy' substituted by `cmy0' on input line
1350.
Package xcolor Info: Model `hsb' substituted by `rgb' on input line 1354.
Package xcolor Info: Model `RGB' extended on input line 1366.
Package xcolor Info: Model `HTML' substituted by `rgb' on input line
1368.
Package xcolor Info: Model `Hsb' substituted by `hsb' on input line 1369.
Package xcolor Info: Model `tHsb' substituted by `hsb' on input line
1370.
Package xcolor Info: Model `HSB' substituted by `hsb' on input line 1371.
Package xcolor Info: Model `Gray' substituted by `gray' on input line
1372.
Package xcolor Info: Model `wave' substituted by `hsb' on input line
1373.
) (c:/texlive/2024/texmf-dist/tex/latex/preprint/authblk.sty
Package: authblk 2001/02/27 1.3 (PWD)
\affilsep=\skip72
\@affilsep=\skip73
\c@Maxaffil=\count305
\c@authors=\count306
\c@affil=\count307
) (c:/texlive/2024/texmf-dist/tex/latex/subfig/subfig.sty
Package: subfig 2005/06/28 ver: 1.3 subfig package
\c@KVtest=\count308
\sf@farskip=\skip74
\sf@captopadj=\dimen197
\sf@capskip=\skip75
\sf@nearskip=\skip76
```

```
\c@subfigure=\count309
\c@subfigure@save=\count310
\c@lofdepth=\count311
\c@subtable=\count312
\c@subtable@save=\count313
\c@lotdepth=\count314
\sf@top=\skip77
\sf@bottom=\skip78
) (c:/texlive/2024/texmf-dist/tex/latex/url/url.sty
\Urlmuskip=\muskip18
Package: url 2013/09/16  ver 3.4  Verb mode for urls, etc.
)
LaTeX Font Info:    Trying to load font information for OT1+ptm on input
line 3
5.
(c:/texlive/2024/texmf-dist/tex/latex/psnfss/ot1ptm.fd
File: ot1ptm.fd 2001/06/04 font definitions for OT1/ptm.
) (c:/texlive/2024/texmf-dist/tex/latex/l3backend/l3backend-pdftex.def
File: l3backend-pdftex.def 2024-05-08 L3 backend support: PDF output
(pdfTeX)
\l__color_backend_stack_int=\count315
\l__pdf_internal_box=\box70
) (./supplementary_information.aux)
\openout1 = `supplementary_information.aux'.

LaTeX Font Info:    Checking defaults for OML/cmm/m/it on input line 35.
LaTeX Font Info:    ... okay on input line 35.
LaTeX Font Info:    Checking defaults for OMS/cmsy/m/n on input line 35.
LaTeX Font Info:    ... okay on input line 35.
LaTeX Font Info:    Checking defaults for OT1/cmr/m/n on input line 35.
LaTeX Font Info:    ... okay on input line 35.
LaTeX Font Info:    Checking defaults for T1/cmr/m/n on input line 35.
LaTeX Font Info:    ... okay on input line 35.
LaTeX Font Info:    Checking defaults for TS1/cmr/m/n on input line 35.
LaTeX Font Info:    ... okay on input line 35.
LaTeX Font Info:    Checking defaults for OMX/cmex/m/n on input line 35.
LaTeX Font Info:    ... okay on input line 35.
LaTeX Font Info:    Checking defaults for U/cmr/m/n on input line 35.
LaTeX Font Info:    ... okay on input line 35.
*geometry* driver: auto-detecting
*geometry* detected driver: pdftex
*geometry* verbose mode - [ preamble ] result:
* driver: pdftex
* paper: letterpaper
* layout: <same size as paper>
* layoutoffset:(h,v)=(0.0pt,0.0pt)
* modes:
* h-part:(L,W,R)=(92.14519pt, 430.00462pt, 92.14519pt)
* v-part:(T,H,B)=(95.39737pt, 556.47656pt, 143.09605pt)
* \paperwidth=614.295pt
* \paperheight=794.96999pt
* \textwidth=430.00462pt
* \textheight=556.47656pt
* \oddsidemargin=19.8752pt
```

```
* \evensidemargin=19.8752pt
* \topmargin=-13.87262pt
* \headheight=12.0pt
* \headsep=25.0pt
* \topskip=10.0pt
* \footskip=30.0pt
* \marginparwidth=65.0pt
* \marginparsep=11.0pt
* \columnsep=10.0pt
* \skip\footins=9.0pt plus 4.0pt minus 2.0pt
* \hoffset=0.0pt
* \voffset=0.0pt
* \mag=1000
* \@twocolumnfalse
* \@twosidefalse
* \@mparswitchfalse
* \@reversemarginfalse
* (1in=72.27pt=25.4mm, 1cm=28.453pt)

*geometry* verbose mode - [ newgeometry ] result:
* driver: pdftex
* paper: letterpaper
* layout: <same size as paper>
* layoutoffset:(h,v)=(0.0pt,0.0pt)
* modes:
* h-part:(L,W,R)=(108.405pt, 397.48499pt, 108.40501pt)
* v-part:(T,H,B)=(72.26999pt, 650.43pt, 72.27pt)
* \paperwidth=614.295pt
* \paperheight=794.96999pt
* \textwidth=397.48499pt
* \textheight=650.43pt
* \oddsidemargin=36.13501pt
* \evensidemargin=36.13501pt
* \topmargin=-37.0pt
* \headheight=12.0pt
* \headsep=25.0pt
* \topskip=10.0pt
* \footskip=30.0pt
* \marginparwidth=65.0pt
* \marginparsep=11.0pt
* \columnsep=10.0pt
* \skip\footins=9.0pt plus 4.0pt minus 2.0pt
* \hoffset=0.0pt
* \voffset=0.0pt
* \mag=1000
* \@twocolumnfalse
* \@twosidefalse
* \@mparswitchfalse
* \@reversemarginfalse
* (1in=72.27pt=25.4mm, 1cm=28.453pt)

(c:/texlive/2024/texmf-dist/tex/context/base/mkii/supp-pdf.mkii
[Loading MPS to PDF converter (version 2006.09.02).]
\scratchcounter=\count316
```

```
\scratchdimen=\dimen198
\scratchbox=\box71
\nofMPsegments=\count317
\nofMParguments=\count318
\everyMPshowfont=\toks37
\MPscratchCnt=\count319
\MPscratchDim=\dimen199
\MPnumerator=\count320
\makeMPintoPDFobject=\count321
\everyMPtoPDFconversion=\toks38
) (c:/texlive/2024/texmf-dist/tex/latex/epstopdf-pkg/epstopdf-base.sty
Package: epstopdf-base 2020-01-24 v2.11 Base part for package epstopdf
Package epstopdf-base Info: Redefining graphics rule for `.eps' on input
line 4
85.
(c:/texlive/2024/texmf-dist/tex/latex/latexconfig/epstopdf-sys.cfg
File: epstopdf-sys.cfg 2010/07/13 v1.3 Configuration of (r)epstopdf for
TeX Liv
e
))
Package caption Info: Begin \AtBeginDocument code.
Package caption Info: subfig package v1.3 is loaded.
Package caption Info: threeparttable package is loaded.
Package caption Info: End \AtBeginDocument code.
! Undefined control sequence.
<argument> Yiwei Fu\,$^{\text {\sfb
                                    1},\dagger }$, Zhonghui Gu\,$^{\text
{\s...
l.48 ...il: dengmh@pku.edu.cn or lhlai@pku.edu.cn}
                                                  \\
The control sequence at the end of the top line
of your error message was never \def'ed. If you have
misspelled it (e.g., `\hobx'), type `I' and the correct
spelling (e.g., `I\hbox'). Otherwise just continue,
and I'll forget about whatever was undefined.

! Undefined control sequence.
<argument> ...ger }$, Zhonghui Gu\,$^{\text {\sfb
                                                  2},\dagger }$, Xiao
Luo\,$...
l.48 ...il: dengmh@pku.edu.cn or lhlai@pku.edu.cn}
                                                  \\
The control sequence at the end of the top line
of your error message was never \def'ed. If you have
misspelled it (e.g., `\hobx'), type `I' and the correct
spelling (e.g., `I\hbox'). Otherwise just continue,
and I'll forget about whatever was undefined.

! Undefined control sequence.
<argument> ...dagger }$, Xiao Luo\,$^{\text {\sfb
                                                  3,}}$, Luhua Lai
\,$^{\tex...
l.48 ...il: dengmh@pku.edu.cn or lhlai@pku.edu.cn}
                                                  \\
```

```
The control sequence at the end of the top line
of your error message was never \def'ed. If you have
misspelled it (e.g., `\hobx'), type `I' and the correct
spelling (e.g., `I\hbox'). Otherwise just continue,
and I'll forget about whatever was undefined.

! Undefined control sequence.
<argument> ...b 3,}}$, Luhua Lai \,$^{\text {\sfb
                                                  2,4,}*}$, and Minghua
Deng...
l.48 ...il: dengmh@pku.edu.cn or lhlai@pku.edu.cn}
                                                  \\
The control sequence at the end of the top line
of your error message was never \def'ed. If you have
misspelled it (e.g., `\hobx'), type `I' and the correct
spelling (e.g., `I\hbox'). Otherwise just continue,
and I'll forget about whatever was undefined.

! Undefined control sequence.
<argument> ...*}$, and Minghua Deng$^{\text {\sfb
                                                  1,4,5,}*}$\\ $^{\text
{\sf...
l.48 ...il: dengmh@pku.edu.cn or lhlai@pku.edu.cn}
                                                  \\
The control sequence at the end of the top line
of your error message was never \def'ed. If you have
misspelled it (e.g., `\hobx'), type `I' and the correct
spelling (e.g., `I\hbox'). Otherwise just continue,
and I'll forget about whatever was undefined.

! Undefined control sequence.
<argument> Yiwei Fu\,$^{\text {\sfb
                                    1},\dagger }$, Zhonghui Gu\,$^{\text
{\s...
l.48 ...il: dengmh@pku.edu.cn or lhlai@pku.edu.cn}
                                                  \\
The control sequence at the end of the top line
of your error message was never \def'ed. If you have
misspelled it (e.g., `\hobx'), type `I' and the correct
spelling (e.g., `I\hbox'). Otherwise just continue,
and I'll forget about whatever was undefined.

! Undefined control sequence.
<argument> ...ger }$, Zhonghui Gu\,$^{\text {\sfb
                                                  2},\dagger }$, Xiao
Luo\,$...
l.48 ...il: dengmh@pku.edu.cn or lhlai@pku.edu.cn}
                                                  \\
The control sequence at the end of the top line
of your error message was never \def'ed. If you have
misspelled it (e.g., `\hobx'), type `I' and the correct
spelling (e.g., `I\hbox'). Otherwise just continue,
and I'll forget about whatever was undefined.
```

```
! Undefined control sequence.
<argument> ...dagger }$, Xiao Luo\,$^{\text {\sfb 
                                                  3,}}$, Luhua Lai 
\,$^{\tex...
l.48 ...il: dengmh@pku.edu.cn or lhlai@pku.edu.cn}
                                                  \\
The control sequence at the end of the top line
of your error message was never \def'ed. If you have
misspelled it (e.g., `\hobx'), type `I' and the correct
spelling (e.g., `I\hbox'). Otherwise just continue,
and I'll forget about whatever was undefined.

! Undefined control sequence.
<argument> ...b 3,}}$, Luhua Lai \,$^{\text {\sfb 
                                                  2,4,}*}$, and Minghua 
Deng...
l.48 ...il: dengmh@pku.edu.cn or lhlai@pku.edu.cn}
                                                  \\
The control sequence at the end of the top line
of your error message was never \def'ed. If you have
misspelled it (e.g., `\hobx'), type `I' and the correct
spelling (e.g., `I\hbox'). Otherwise just continue,
and I'll forget about whatever was undefined.

! Undefined control sequence.
<argument> ...*}$, and Minghua Deng$^{\text {\sfb 
                                                  1,4,5,}*}$\\ $^{\text 
{\sf...
l.48 ...il: dengmh@pku.edu.cn or lhlai@pku.edu.cn}
                                                  \\
The control sequence at the end of the top line
of your error message was never \def'ed. If you have
misspelled it (e.g., `\hobx'), type `I' and the correct
spelling (e.g., `I\hbox'). Otherwise just continue,
and I'll forget about whatever was undefined.


! LaTeX Error: There's no line here to end.

See the LaTeX manual or LaTeX Companion for explanation.
Type  H <return>  for immediate help.
 ...

l.49 \maketitle

Your command was ignored.
Type  I <command> <return>  to replace it with another command,
or  <return>  to continue without it.

LaTeX Font Info:    Trying to load font information for U+msa on input
line 49.

(c:/texlive/2024/texmf-dist/tex/latex/amsfonts/umsa.fd
File: umsa.fd 2013/01/14 v3.01 AMS symbols A
```

```
)
LaTeX Font Info:    Trying to load font information for U+msb on input
line 49.

(c:/texlive/2024/texmf-dist/tex/latex/amsfonts/umsb.fd
File: umsb.fd 2013/01/14 v3.01 AMS symbols B
)
LaTeX Font Info:    Trying to load font information for U+rsfs on input
line 49
.
(c:/texlive/2024/texmf-dist/tex/latex/jknapltx/ursfs.fd
File: ursfs.fd 1998/03/24 rsfs font definition file (jk)
)
LaTeX Font Info:    Trying to load font information for OT1+phv on input
line 4
9.
(c:/texlive/2024/texmf-dist/tex/latex/psnfss/ot1phv.fd
File: ot1phv.fd 2020/03/25 scalable font definitions for OT1/phv.
)

Package natbib Warning: Citation `gligorijevic2021structure' on page 1
undefine
d on input line 51.


Package natbib Warning: Citation `berman2000protein' on page 1 undefined
on inp
ut line 51.


Package natbib Warning: Citation `dana2019sifts' on page 1 undefined on
input l
ine 51.


Package natbib Warning: Citation `mirdita2021fast' on page 1 undefined on
input
 line 52.


Package natbib Warning: Citation `gligorijevic2021structure' on page 1
undefine
d on input line 56.


Package natbib Warning: Citation `ashburner2000gene' on page 1 undefined
on inp
ut line 56.


Package natbib Warning: Citation `gligorijevic2021structure' on page 1
undefine
d on input line 58.
```

```
Package natbib Warning: Citation `waterhouse2018swiss' on page 1
undefined on i
nput line 58.


Package natbib Warning: Citation `gu2023hierarchical' on page 1 undefined
on in
put line 58.

LaTeX Font Info:    Trying to load font information for OML+ptm on input
line 5
8.
(c:/texlive/2024/texmf-dist/tex/latex/psnfss/omlptm.fd
File: omlptm.fd
)
LaTeX Font Info:    Font shape `OML/ptm/m/n' in size <10> not available
(Font)              Font shape `OML/cmm/m/it' tried instead on input line
58.

Package natbib Warning: Citation `varadi2022alphafold' on page 1
undefined on i
nput line 58.



[1


{c:/texlive/2024/texmf-
var/fonts/map/pdftex/updmap/pdftex.map}{c:/texlive/2024/
texmf-dist/fonts/enc/dvips/base/8r.enc}]

Package natbib Warning: Citation `altschul1990basic' on page 2 undefined
on inp
ut line 67.


Package natbib Warning: Citation `gligorijevic2021structure' on page 2
undefine
d on input line 67.


Package natbib Warning: Citation `kulmanov2021deepgoplus' on page 2
undefined o
n input line 71.


Package natbib Warning: Citation `cao2021tale' on page 2 undefined on
input lin
e 74.
```

```
Package natbib Warning: Citation `buchfink2015fast' on page 2 undefined
on inpu
t line 74.


Package natbib Warning: Citation `gligorijevic2021structure' on page 2
undefine
d on input line 76.


Package natbib Warning: Citation `ma2022enhancing' on page 2 undefined on
input
 line 78.


Package natbib Warning: Citation `gu2023hierarchical' on page 2 undefined
on in
put line 80.

LaTeX Font Info:    Calculating math sizes for size <8.5> on input line
88.

LaTeX Font Warning: Font shape `OT1/cmr/m/n' in size <8.5> not available
(Font)              size <8> substituted on input line 88.


LaTeX Font Warning: Font shape `OT1/cmr/m/n' in size <4.25> not available
(Font)              size <5> substituted on input line 88.


LaTeX Font Warning: Font shape `OML/cmm/m/it' in size <8.5> not available
(Font)              size <8> substituted on input line 88.


LaTeX Font Warning: Font shape `OML/cmm/m/it' in size <4.25> not
available
(Font)              size <5> substituted on input line 88.


LaTeX Font Warning: Font shape `OMS/cmsy/m/n' in size <8.5> not available
(Font)              size <8> substituted on input line 88.


LaTeX Font Warning: Font shape `OMS/cmsy/m/n' in size <4.25> not
available
(Font)              size <5> substituted on input line 88.


LaTeX Font Warning: Font shape `U/rsfs/m/n' in size <8.5> not available
(Font)              size <8> substituted on input line 88.


LaTeX Font Warning: Font shape `U/rsfs/m/n' in size <4.25> not available
(Font)              size <5> substituted on input line 88.
```

```
Overfull \hbox (64.41841pt too wide) in paragraph at lines 88--104
 [][]
 []


Overfull \hbox (64.41841pt too wide) in paragraph at lines 110--127
 [][]
 []


Overfull \hbox (64.41841pt too wide) in paragraph at lines 133--150
 [][]
 []


LaTeX Warning: `h' float specifier changed to `ht'.


LaTeX Warning: `h' float specifier changed to `ht'.


Underfull \vbox (badness 10000) has occurred while \output is active []



[2]
Package epstopdf Info: Source file: <figureS1.eps>
(epstopdf)                    date: 2024-10-18 09:17:58
(epstopdf)                    size: 24451 bytes
(epstopdf)             Output file: <figureS1-eps-converted-to.pdf>
(epstopdf)                    date: 2024-10-18 09:18:26
(epstopdf)                    size: 10042 bytes
(epstopdf)             Command: <repstopdf --outfile=figureS1-eps-
converted-to.
pdf figureS1.eps>
(epstopdf)             \includegraphics on input line 158.
Package epstopdf Info: Output file is already uptodate.
<figureS1-eps-converted-to.pdf, id=18, 462.52798pt x 346.89601pt>
File: figureS1-eps-converted-to.pdf Graphic file (type pdf)
<use figureS1-eps-converted-to.pdf>
Package pdftex.def Info: figureS1-eps-converted-to.pdf  used on input
line 158.

(pdftex.def)             Requested size: 317.9892pt x 238.50099pt.


[3]
Overfull \hbox (1.7247pt too wide) in paragraph at lines 193--208
 [][]
 []
```

```
LaTeX Warning: `h' float specifier changed to `ht'.

Package epstopdf Info: Source file: <figureS2.eps>
(epstopdf)                    date: 2024-10-18 09:17:58
(epstopdf)                    size: 59022 bytes
(epstopdf)             Output file: <figureS2-eps-converted-to.pdf>
(epstopdf)                    date: 2024-10-18 09:18:29
(epstopdf)                    size: 71129 bytes
(epstopdf)             Command: <repstopdf --outfile=figureS2-eps-
converted-to.
pdf figureS2.eps>
(epstopdf)             \includegraphics on input line 237.
Package epstopdf Info: Output file is already uptodate.
<figureS2-eps-converted-to.pdf, id=23, 803.0pt x 602.25pt>
File: figureS2-eps-converted-to.pdf Graphic file (type pdf)
<use figureS2-eps-converted-to.pdf>
Package pdftex.def Info: figureS2-eps-converted-to.pdf  used on input
line 237.

(pdftex.def)             Requested size: 317.9892pt x 238.49724pt.


[4 <./figureS1-eps-converted-to.pdf>]
Package epstopdf Info: Source file: <figureS3.eps>
(epstopdf)                    date: 2024-10-18 09:17:58
(epstopdf)                    size: 48033 bytes
(epstopdf)             Output file: <figureS3-eps-converted-to.pdf>
(epstopdf)                    date: 2024-10-18 09:18:31
(epstopdf)                    size: 97185 bytes
(epstopdf)             Command: <repstopdf --outfile=figureS3-eps-
converted-to.
pdf figureS3.eps>
(epstopdf)             \includegraphics on input line 243.
Package epstopdf Info: Output file is already uptodate.
<figureS3-eps-converted-to.pdf, id=51, 803.0pt x 602.25pt>
File: figureS3-eps-converted-to.pdf Graphic file (type pdf)
<use figureS3-eps-converted-to.pdf>
Package pdftex.def Info: figureS3-eps-converted-to.pdf  used on input
line 243.

(pdftex.def)             Requested size: 317.9892pt x 238.49724pt.
Package epstopdf Info: Source file: <figureS4.eps>
(epstopdf)                    date: 2024-10-18 09:17:58
(epstopdf)                    size: 48389 bytes
(epstopdf)             Output file: <figureS4-eps-converted-to.pdf>
(epstopdf)                    date: 2024-10-18 09:18:34
(epstopdf)                    size: 84042 bytes
(epstopdf)             Command: <repstopdf --outfile=figureS4-eps-
converted-to.
pdf figureS4.eps>
(epstopdf)             \includegraphics on input line 249.
Package epstopdf Info: Output file is already uptodate.
<figureS4-eps-converted-to.pdf, id=52, 803.0pt x 602.25pt>
File: figureS4-eps-converted-to.pdf Graphic file (type pdf)
```

```
<use figureS4-eps-converted-to.pdf>
Package pdftex.def Info: figureS4-eps-converted-to.pdf  used on input
line 249.

(pdftex.def)             Requested size: 317.9892pt x 238.49724pt.


[5 <./figureS2-eps-converted-to.pdf>]

[6 <./figureS3-eps-converted-to.pdf> <./figureS4-eps-converted-to.pdf>]
No file supplementary_information.bbl.

Package natbib Warning: There were undefined citations.

(./supplementary_information.aux)
 ***********
LaTeX2e <2024-06-01> patch level 2
L3 programming layer <2024-05-27>
 ***********

LaTeX Font Warning: Size substitutions with differences
(Font)              up to 0.75pt have occurred.

 )
Here is how much of TeX's memory you used:
 7314 strings out of 473583
 114099 string characters out of 5732343
 1949908 words of memory out of 5000000
 30091 multiletter control sequences out of 15000+600000
 578971 words of font info for 84 fonts, out of 8000000 for 9000
 1141 hyphenation exceptions out of 8191
 72i,11n,79p,1568b,336s stack positions out of
10000i,1000n,20000p,200000b,200000s
<c:/texlive/2024/texmf-
dist/fonts/type1/public/amsfonts/cm/cmmi10.pfb><c:/tex
live/2024/texmf-
dist/fonts/type1/public/amsfonts/cm/cmmi7.pfb><c:/texlive/2024/
texmf-
dist/fonts/type1/public/amsfonts/cm/cmr10.pfb><c:/texlive/2024/texmf-dist
/fonts/type1/public/amsfonts/cm/cmr7.pfb><c:/texlive/2024/texmf-
dist/fonts/type
1/public/amsfonts/cm/cmsy10.pfb><c:/texlive/2024/texmf-
dist/fonts/type1/public/
amsfonts/cm/cmsy7.pfb><c:/texlive/2024/texmf-
dist/fonts/type1/public/amsfonts/c
m/cmsy8.pfb><c:/texlive/2024/texmf-
dist/fonts/type1/urw/helvetic/uhvr8a.pfb><c:
/texlive/2024/texmf-
dist/fonts/type1/urw/times/utmb8a.pfb><c:/texlive/2024/texm
f-dist/fonts/type1/urw/times/utmr8a.pfb>
Output written on supplementary_information.pdf (6 pages, 366157 bytes).
PDF statistics:
 108 PDF objects out of 1000 (max. 8388607)
 52 compressed objects within 1 object stream
```

```
0 named destinations out of 1000 (max. 500000)
21 words of extra memory for PDF output out of 10000 (max. 10000000)
```

Click here to access/download;LaTeX - Figure (eps, ps, etc.);example-grid-100x100pt.png

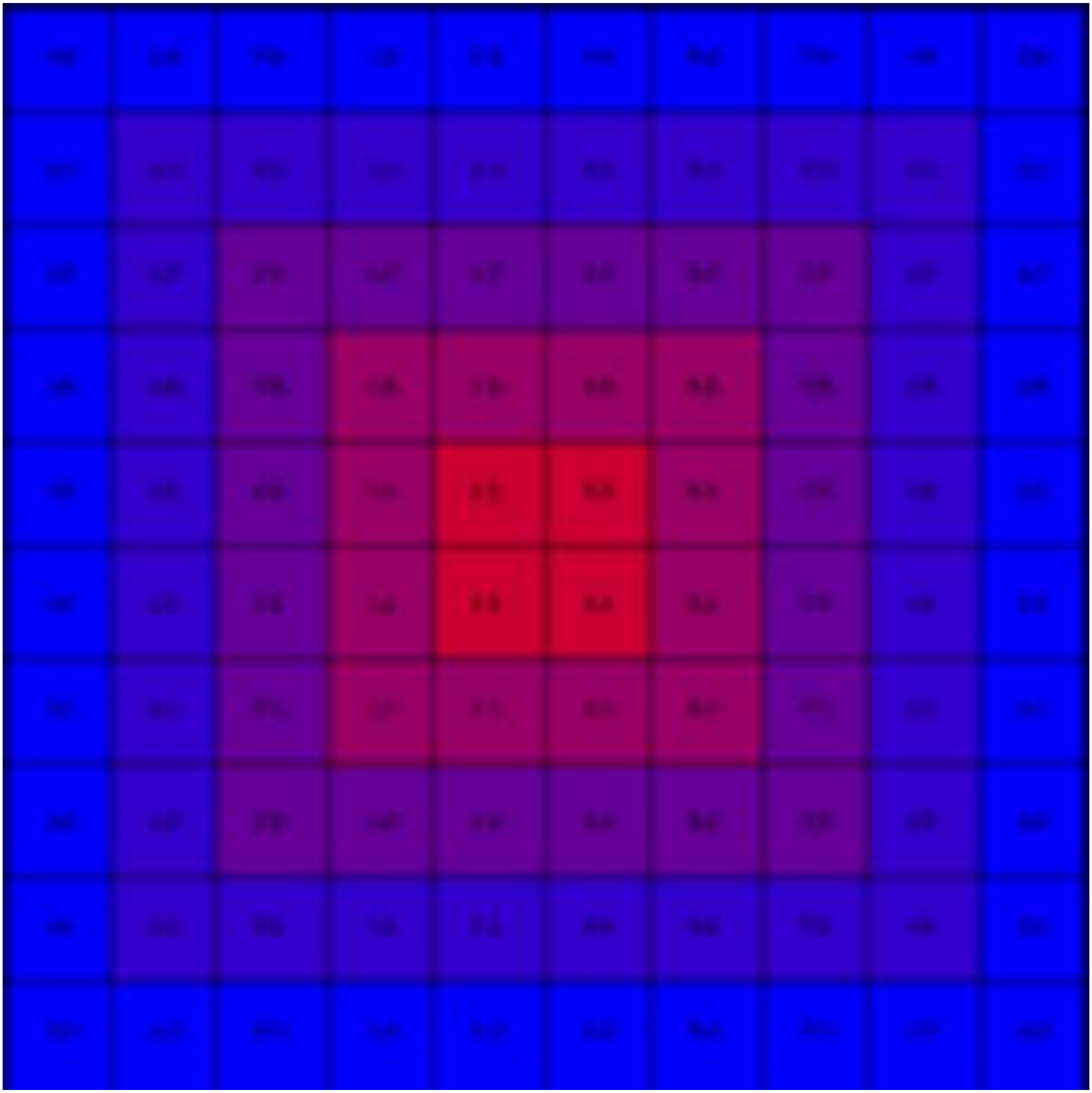

Click here to access/download;LaTeX - Figure (eps, ps, etc.);example-image.png

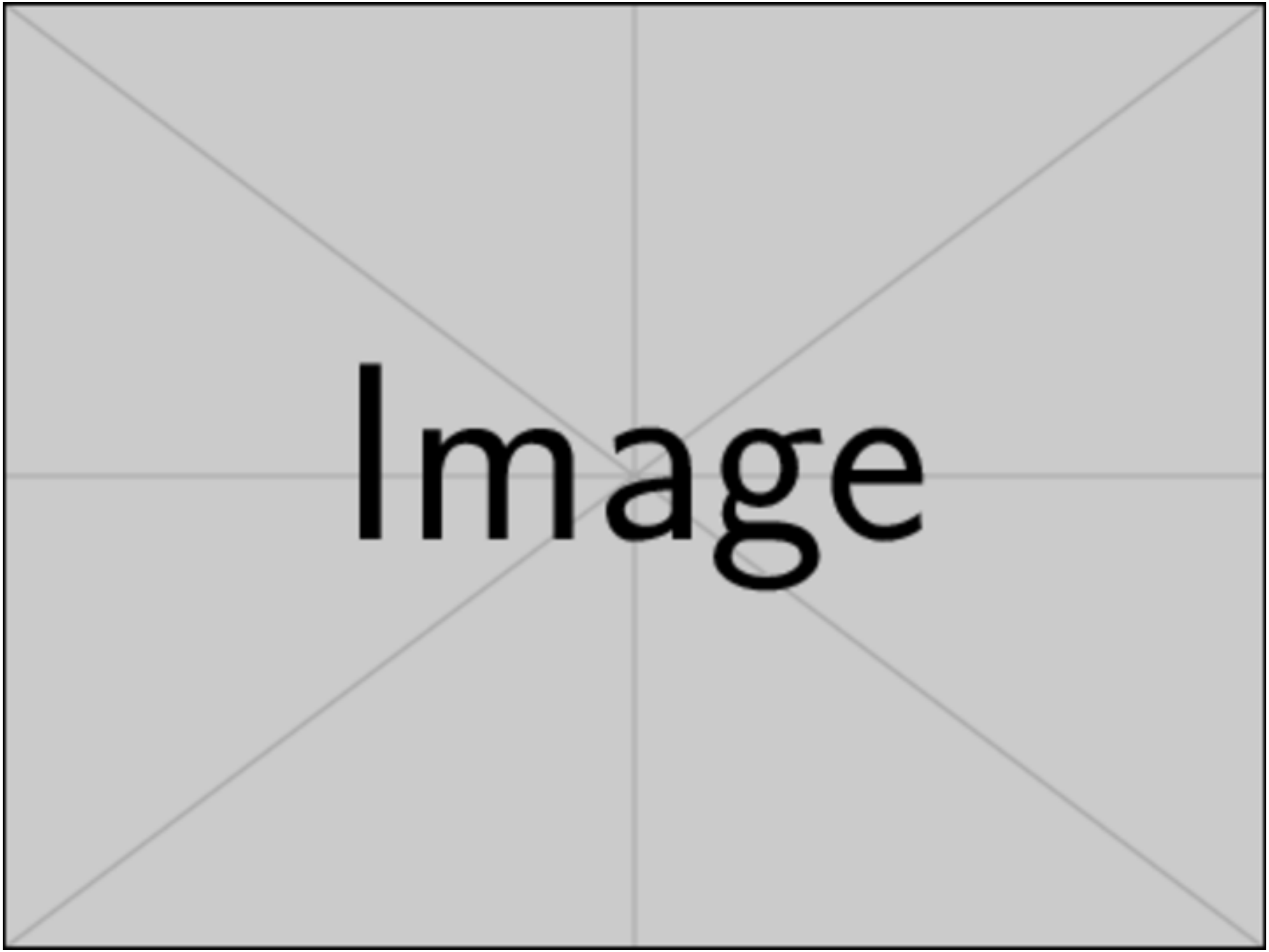

Click here to access/download;LaTeX - Figure (eps, ps, etc.);figureS5.eps

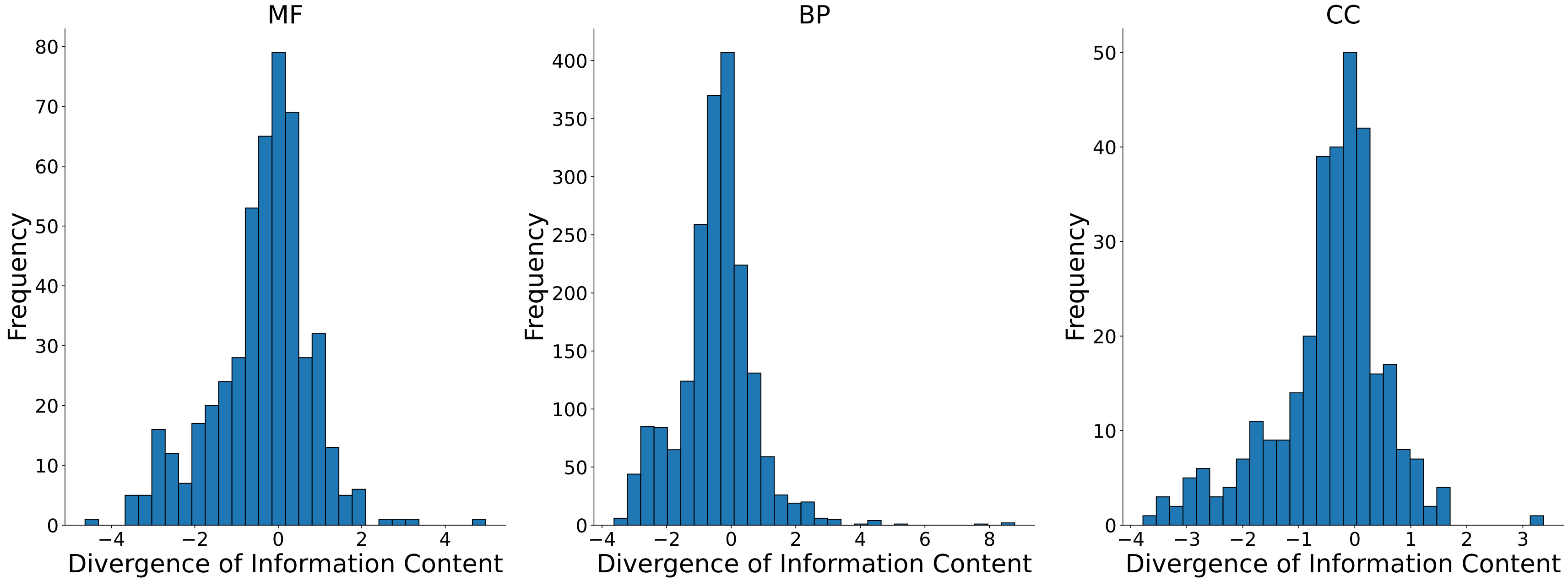

Click here to access/download;Manuscript;GALA_revision_3.pdf

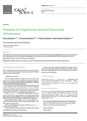

GigaScience, 20xx, 1–11

doi: xx.xxxx/xxxx
Manuscript in Preparation

PAPER

Learning A Generalized Graph Transformer for Protein Function Prediction in Dissimilar Sequences

Yiwei Fu [1,†], Zhonghui Gu [2,†], Xiao Luo [3], Qirui Guo[4], Luhua Lai[2,4,*] and Minghua Deng [1,4,5,*]

[1]School of Mathematics Sciences, Peking University, 100871, Beijing, China and [2]Peking-Tsinghua Center for Life Sciences, Peking University, 100871, Beijing, China and [3]Department of Computer Science, University of California, 90024, Los Angeles, USA and [4]Center for Quantitative Biology, Peking University, 100871, Beijing, China and [5]Center for Statistical Science, Peking University, 100871, Beijing, China

[*]Correspondence E-mail: dengmh@math.pku.edu.cn; lhlai@pku.edu.cn
[†]Contributed equally.

Abstract

Background: In the face of a growing disparity between high-throughput sequence data and low-throughput experimental studies, the emerging field of deep learning stands as a promising alternative. Generally, many data-driven approaches are capable of facilitating fast and accurate predictions of protein functions. Nevertheless, the inherent statistical nature of deep learning techniques may limit their generalization capabilities when applied to novel non-homologous proteins that diverge significantly from existing ones.
Results: In this work, we propose a novel, generalized approach named Graph Adversarial Learning with Alignment (GALA) for protein function prediction. Our GALA model integrates a graph transformer architecture with an attention pooling module to extract information from both protein sequences and structures, facilitating unified learning of protein structural representations. Particularly noteworthy, GALA incorporates a domain discriminator conditioned on both representations and predicted probabilities, which undergoes adversarial training to ensure representation invariance across diverse environments. To optimize the model with abundant label information, we generate label embeddings in the hidden space, explicitly aligning them with protein representations. Benchmarked on datasets derived from PDB database and Swiss-Prot database,our GALA achieves performance comparable to several state-of-the-art methods. Furthermore, GALA demonstrates outstanding interpretability by identifying key functional residues associated with GO terms through class activation mapping.
Conclusions: GALA, which leverages adversarial learning and label embedding alignment to acquire domain-invariant protein representations, exhibits outstanding generalizability in function prediction for proteins from previously unseen sequence space. By utilizing the structures predicted by AlphaFold2, GALA holds significant potential for function annotation in newly discovered sequences. Implementations of our GALA can be found at `https://github.com/fuyw-aisw/GALA`.

Key words: protein function prediction; low sequence identity; domain adaptation; adversarial learning; graph transformer

Introduction

Proteins are the main catalysts, structural elements, signaling messengers and molecular machines of biological tissues [1]. Additionally, protein function prediction is a pivotal challenge in comprehending the roles of proteins within biological systems, which holds significant implications for disease research, drug discovery, and various domains of biotechnology and bioinformatics. The advancement of high-throughput sequencing technology has resulted in the creation of vast protein sequence databases [2, 3, 4, 5], yet a notable proportion of these proteins lack functional annotations. Experimentally determining the functional properties of protein se-

Compiled on: October 18, 2024.
Draft manuscript prepared by the author.

Key Points

- We present GALA, a novel and generalized approach for protein function prediction, leveraging adversarial learning and label embedding alignment to ensure representation invariance across diverse environments and dissimilar protein sequences.
- Comprehensive experimental evaluations demonstrate that GALA outperforms several state-of-the-art methods, exhibiting excellent generalizability and interpretability. This positions GALA as well-suited for protein function prediction in dissimilar sequences

quences is not only labor-intensive but also time-consuming [6]. In response to this challenge, a wide range of computational methods have been proposed for predicting protein functions [7, 8, 9, 10].

Traditional sequence-alignment based methods [11, 12] are utilized to transfer the functions from similar annotated sequences or domains to query sequences, assuming that proteins with similar sequences and structures are more likely to have similar functions [13]. For instance, Blast [11] is a basic method to transfer annotations directly from homologous sequences with labeled protein functions, which cannot make confident prediction on proteins without annotated homologous sequences in the real scenarios.

Furthermore, machine learning-based methods are developed leveraging existing information, such as amino acid sequences [8, 14, 15, 16, 17], protein-protein interactions [9, 18, 19, 20, 21, 22], evolutionary relations [23], experimentally resolved or predicted protein structures [10, 24, 25, 26, 27], literature [28] and aforementioned multi-source information [29]. In general, the amino acid sequences of proteins are readily available, while other features of proteins, such as protein-protein interactions and structures, may present in a small subset of proteins. This has led to the emergence of a large number of sequence-based methods. For example, TALE+ [16] utilize protein sequence inputs jointly embedded with hierarchical function labels to enhance protein function prediction without considering structural information. Furthermore, DeepGOPlus[17] combines a deep convolutional neural network (CNN) model with sequence similarity based predictions for predicting protein functions from sequence alone. Considering that protein structures have a direct relationship with functions, utilizing protein structures for function prediction may have a natural advantage over these sequence-based methods. Among the notable structure-based methods, DeepFRI [10] pioneers the use of protein structures generated by homology modeling for reinforcement, achieving comparable performance with good interpretability. More importantly, I-TASSER-MTD [30] is specifically designed to model the structures and functions of multi-domain proteins. Notably, although some protein structures have not been experimentally resolved, tools like AlphaFold2 [31], RoseTTAFold [32] and ESMFold [33] have demonstrated remarkable success in protein structure prediction. Furthermore, Struct2GO [26] validates the hypothesis that AlphaFold-predicted structures could improve protein function prediction performance. And NetGO 3.0 [34] has proposed a new component logistic regression (LR)-ESM based on NetGO 2.0 [29] to improve large-scale functional annotations. HEAL [27] employs a hierarchical graph transformer combined with graph contrastive learning to maximize similarity between different views represented by the graph. However, these deep learning-based methods may rely to some extent on homology information of sequences and models, potentially compromising their ability to transfer protein function prediction information from known to unknown dissimilar sequences, resulting in less satisfactory performance. MetaGO [35] is proposed to predict Gene Ontology of non-homologous proteins by combining three complementary pipelines from global and local structure alignments, sequence and sequence-profile matches, and protein-protein interaction (PPI) network mapping. However, the quality of PPI network including data noise and data completeness may affect the performance of function prediction. In conclusion, there are promising prospects for the development of highly generalized prediction frameworks that demonstrate superior performance on dissimilar target datasets compared to their source datasets.

To address the aforementioned challenges and formalize a generalized framework, we propose a novel domain adaptation approach named Graph Adversarial Learning with Alignment (GALA) for protein function prediction. To thoroughly explore protein structure and capture essential residues from diverse environments, we introduce a graph transformer with an attention mechanism for representation learning. The transformer first generates metanode embeddings to interact with other residues, followed by aggregating node embeddings for better protein representations. To enhance generalizability, we introduce a domain discriminator conditioned on both representations and predictions, which is trained adversarially for discrepancy reduction between the source and target domains in the embedding space. In addition, to improve the discriminability of protein representations, we generate label embeddings in the latent space, and enforce source representations to approach their corresponding label embeddings compared to other embeddings. In this way, we can produce discriminative and domain-invariant protein representations for more accurate function prediction.

To assess the performance of GALA, we compare it with several baseline methods, including Blast [11], DeepGOPlus[17], TALE+ [16], DeepFRI [10], Struct2GO [26], and HEAL [27], in various settings. We retrain these models with our split training sets and then evaluate their performance on the protein test sets in three functional aspects: Molecular Function (MF), Biological Process (BP), and Cellular Component (CC). To demonstrate the efficiency of GALA, we derive two versions of the model: GALA-PDB and GALA. The former is trained with a subset of proteins, while the latter incorporates AlphaFold2-predicted protein structures into training. And Our model has achieved outstanding performance across all three aspects. Furthermore, we evaluate their performance on distinct specificity GO terms, and GALA proves to be robust to GO terms with varying specificity, particularly for rare GO terms. On the test set of AlphaFold2-predicted protein structures, GALA significantly outperforms all other methods.What's more, GALA demonstrates exceptional performance on the non-homologous PDBch test set. Notably, GALA excels on the newly annotated test set, underscoring its robustness over time. More importantly, our method GALA showcases excellent generalizability and interpretability in identifying crucial residues, making it suitable for protein function prediction in dissimilar sequences. Finally, We conduct an ablation study on our method GALA to assess the utility of each module.

Methods

Problem Definition

We begin with the problem setting and notations. Previous protein function prediction approaches [27] usually assume that both training and test samples are from the same distribution, which cannot be promised when novel proteins are found in the real world. To-

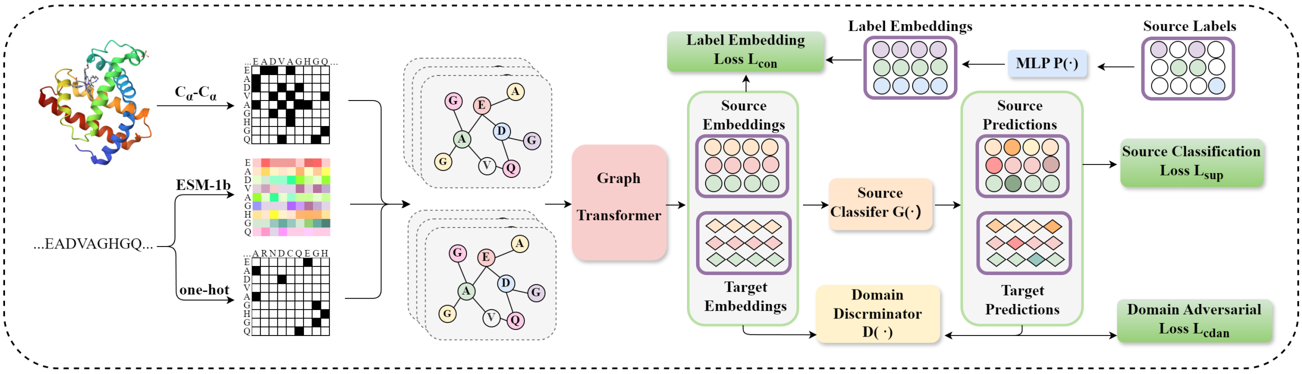

Figure 1. Overview of our proposed method GALA. GALA first adopts a GCN-based encoder to aggregate local niche information and obtain node-level feature embeddings for each graph. Subsequently, a multi-head meta-nodes graph transformer is introduced to thoroughly explore protein structure, utilizing attention pooling module to aggregate graph-level representations. To better represent protein graphs, a two-layer multi-layer perception is applied to generate label embeddings in the latent space for labeled source data and then embed graph representations with label information for better function prediction. To enhance the generalizability of GALA, a domain adversarial discriminator is applied to narrow the discrepancy between source and target domains, and align various domains with low sequence identity.

wards this end, we study a relatively underexplored but more practical setting of domain adaptive protein function prediction. Here, we have access to a labeled source domain $\mathcal{D}^s = \{(G_i^s, y_i^s)\}_{i=1}^{n_s}$ with n_s protein graphs and an unlabeled target domain $\mathcal{D}^t = \{(G_j^t)\}_{j=1}^{n_t}$ with n_t graphs. $\mathcal{D}^s$ and $\mathcal{D}^t$ share the same label space, that is, $\mathcal{Y} = \{1, 2, \ldots, C\}$ with different distributions in the data space. Therefore, our objective is to minimize the discrepancy among diverse domains within the embedding space, thus enhancing the model's generalizability and enabling the seamless transfer of protein function label information from the source domain to the target domain.

To characterize the spatial structure, we represent each protein using a graph $G = (\mathcal{V}, \mathcal{E})$, where $\mathcal{V}$ and $\mathcal{E}$ represent the node and edge sets, respectively. Specifically, the node set $\mathcal{V}$ comprises the amino acid residue sequence of a graph with $|\mathcal{V}|$ residues. Regarding the edge set, it is derived from the C_α-C_α contact map. We define two amino acid residues as adjacent if the distance between their C_α atoms is less than 10 Å. Subsequently, we add an edge between adjacent residues and construct an adjacency matrix $A \in \mathcal{R}^{|\mathcal{V}| \times |\mathcal{V}|}$. Additionally, the node feature matrix $X \in \mathcal{R}^{|\mathcal{V}| \times F}$ is obtained from two sources: (i) a one-hot residue encoder encoded by amino acid symbols, and (ii) the ESM-1b protein language model [36], which produces residue embeddings to capture intrinsic protein sequence knowledge. These embeddings are then concatenated to form the feature matrix.

An Overview of the Proposed GALA

In this paper, we propose a new approach named GALA for protein function prediction in dissimilar sequences. Our GALA utilizes the graph transformer to acquire graph-level embeddings that capture spatial semantics and essential information about key residues. In addition, a domain classifier is introduced conditioned on both representations and predictions, facilitating the acquisition of domain-invariant features by adversarial learning. Finally, label embedding alignment is adopted to enhance the discriminability of protein representations. For a more comprehensive understanding, please refer to the detailed information provided in **Figure 1**.

Graph Transformer for Representation Learning

We first employ a graph convolutional network to capture the overall structure of the graph [37, 38]. In this process, the node embeddings are gradually updated by aggregating information from the nodes' neighborhood in the last layer. The embeddings are updated with the following layer-wise rule.

$$H^{l+1} = \sigma(\tilde{D}^{-\frac{1}{2}}\tilde{A}\tilde{D}^{-\frac{1}{2}}H^l W^l). \tag{1}$$

Here, $\tilde{A}$ is the adjacency matrix of protein graph G with added self-loops. $\tilde{D}$ is the degree matrix and W^l is a layer-specific weight matrix which can be learnable. Furthermore, $\sigma(\cdot)$ is an activation function and $ReLU(\cdot) = max(0, \cdot)$ is applied during training. H^l is the embedding matrix in the l-th layer and $H^0 = X$. After N layers, we generate the hidden embedding matrix $H \triangleq H^N \in \mathcal{R}^{|\mathcal{V}| \times D}$, where D is the dimension of hidden embeddings. After applying the graph convolutional network (GCN), we generate node-level embeddings for each graph.

To effectively integrate residue neighborhood information and represent protein structural information, we are required to aggregate node-level representations for each graph. In particular, we introduce K meta-nodes, denoted as learnable features $q_1, \ldots, q_K$, to interact with node embeddings and then capture the protein structure information. Inspired by the graph transformer [39], we obtain key and value embedding vectors $\mathcal{K} \in \mathcal{R}^{|\mathcal{V}| \times D}$ and $\mathcal{V} \in \mathcal{R}^{|\mathcal{V}| \times D}$ from another two graph convolution networks leveraging the graph structure, and the concatenated meta-node representation $\mathcal{Q} = (q_1, \ldots, q_k) \in \mathcal{R}^{K \times D}$ performs as query vector. We calculate the similarity between $\mathcal{Q}$ and $\mathcal{K}$ to obtain weights, which are then used to weight value vector $\mathcal{V}$, and finally derive the meta-node embedding matrix $\Gamma \in \mathcal{R}^{K \times D}$ using the following formula:

$$\Gamma = softmax\left(\frac{\mathcal{Q} \cdot \mathcal{K}^T}{\sqrt{D}}\right) \cdot \mathcal{V}, \tag{2}$$

$$\mathcal{K} = GCN^1(H, A), \quad \mathcal{V} = GCN^2(H, A). \tag{3}$$

Instead of computing a single attention, we can further utilize multi-head attention [40]. This involves repeating the above formula for h times with distinct parameters, resulting in h different representation subspaces, denoted as $\Gamma_1, \ldots, \Gamma_h$. Subsequently, we concatenate these h derived meta-node embeddings and transform them to a multi-head meta-node embedding using a fully connected network. In other words,

$$U = FC^1([\Gamma_1, \ldots, \Gamma_h]), \tag{4}$$

where FC^1 denotes a multi-layer perceptron (MLP), and thus $U \in \mathcal{R}^{K \times D}$ is a multi-head meta-nodes embedding matrix, which represents structure information in the protein graph.

In order to aggregate local niche information, we adopt an attention module, which summarizes these multi-head meta-node

representations into a graph-level representation in an adaptive fashion. Specifically, we utilize a query vector $\mathcal{Q}^P \in \mathcal{R}^D$ and two transformation matrices $\mathcal{K}^P \in \mathcal{R}^{D\times D}$ and $\mathcal{V}^P \in \mathcal{R}^{D\times D}$, and then the graph representation z is derived by the following formula:

$$z = softmax\left(\frac{\mathcal{Q}^P \cdot (U \cdot \mathcal{K}^P)^T}{\sqrt{D}}\right) \cdot U \cdot \mathcal{V}^P. \quad (5)$$

Finally, we construct a source classifier G to establish a projection between the graph representation z and the label y, and the predicted positive probability is denoted as $\hat{y} = G(z)$. A binary cross-entropy loss objective function for multi-label classification of labeled source data is described as follows:

$$\mathcal{L}_{sup} = -\frac{1}{M \cdot C}\sum_{c=1}^{C}\sum_{m=1}^{M}\left(y_{mc}log(\hat{y}_{mc}) + (1 - y_{mc})log(1 - \hat{y}_{mc})\right). \quad (6)$$

where M is the sample size of a minibatch, and C is the number of classes. What's more, y_{mc} and $\hat{y}_{mc}$ denote the ground truth and predicted probability for the c-th function of m-th sample respectively.

Adversarial Learning for Domain Alignment

Previous methods [10] typically neglect domain alignment to some extent, assuming that the source and target domains inherently share the same distribution. Consequently, annotating novel proteins dissimilar to known functional proteins presents significant challenges. The key to addressing this issue is to minimize the gap between the source and target domains and subsequently learn domain-invariant features to achieve cross-domain protein functional annotation. Here, we introduce a domain discriminator to learn domain-invariant graph representations to transfer annotations from the source domain to the target domain.

Specifically, we leverage adversarial learning [41] to obtain domain-invariant graph representations, which can be formulated as an optimization problem involving source classifier G and domain discriminator D across the source and target domains. We randomly sample a minibatch of M graphs from source and target data respectively, and a binary cross-entropy loss is employed to distinguish whether a sample is from the source domain or the target domain. Let z and $\hat{y}$ denote the outputs of feature extractor F and source classifier G, respectively. The adversarial learning loss is formulated as follows:

$$\mathcal{L}_{adv} = -\frac{1}{M}\sum_{i=1}^{M} w(H(\hat{y}_i^s))\log D(T(z_i^s, \hat{y}_i^s)) - \frac{1}{M}\sum_{j=1}^{M} w(H(\hat{y}_j^t))\log\left(1 - D(T(z_j^t, \hat{y}_j^t))\right), \quad (7)$$

where z_i^s and z_j^t denote graph embeddings of graph i and graph j from the source data and target data. Moreover, $\hat{y}_i^s$ and $\hat{y}_j^t$ are predicted probabilities of graphs, and D denotes domain discriminator. $T(z_i^s, \hat{y}_i^s) = \frac{1}{\sqrt{d}}(R_z z_i^s) \odot (R_{\hat{y}}\hat{y}_i^s)$ represents the explicit randomized multilinear map of dimension d. Here, $\odot$ denotes the element-wise product, and R_z and $R_{\hat{y}}$ are random matrices sampled only once and held constant throughout training. Each element $R_{i,j}$ follows a symmetric distribution with univariance. The mapping is used to capture multiplicative interactions between feature representation and classifier prediction, which is important to learn domain-invariant features. The entropy-aware weight, denoted as $w(H(\hat{y}_i^s)) = 1 + e^{-H(\hat{y}_i^s)}$, is employed to adjust the weights of samples. This aims to prioritize the discriminator's focus on examples that are easier to transfer, as indicated by more certain predictions. Through adversarial learning, we align source and target domains, which is beneficial for obtaining domain-invariant representations and consequently improving model generalizability.

Label Embedding Alignment

Inspired by TALE [16], we introduce a label embedding alignment module, which first generates label embeddings for source data in the representation space and then aligns graph and label embeddings in the hidden space, aiding the learning of semantics from labeled source data. Specifically, we employ a two-layer multi-layer perception (MLP) $P(\cdot)$ to project each label representation y into a label embedding b with the same dimension as z, i.e., $b = P(y)$. The subsequent goal is to align z and b for each protein, so that label information is contained in graph embedding. Comprehensively, we sample a minibatch of M proteins, each of which produces graph embedding z and label embedding b, and then the loss function for label embedding alignment is written as follows:

$$\mathcal{L}_{con} = -\frac{1}{M}\sum_{i=1}^{M} log\frac{e^{z_i^s * b_i^s/\tau}}{\sum_{j\neq i} e^{z_i^s * b_j^s/\tau}}, \quad (8)$$

where τ denotes a temperature parameter and $*$ calculates the cosine similarity between graph and label embeddings of labeled source data. After the alignment, we can effectively generate protein embeddings that enrich label information.

Total Loss and Model Training

The final loss function is derived by combining the above losses as:

$$\mathcal{L} = \mathcal{L}_{sup} + \mathcal{L}_{adv} + \mathcal{L}_{con}. \quad (9)$$

We train the proposed model using Adam [42] with learning rate 1e-4 and we adopt SGD to train the domain discriminator with momentum 0.9 and learning rate 0.03. All modules are trained utilizing a single A100-PCIE 80GB graphics processing unit (GPU), with training times of approximately two hours using a batch size of 64. Moreover, the running times for several protein cases are provided in Table S6 of the supplementary materials.

Dataset

In our experiments, we utilize the same dataset, named PDBch, from DeepFRI [10] work, which consists of 36,641 experimentally solved protein structures from the PDB database [2] and their associated Gene Ontology (GO) terms sourced from SIFTS [43]. To ensure dissimilarity between our training and test sets, we employ the MMseqs [44] sequence clustering tool with a sequence identity threshold of 30%. What's more, the training, validation, and test sets are then selected from different clusters, with an approximate ratio of 8:1:1, ensuring that the sequence identity between samples from different sets is below 30%. While the sequence identity among different sets is low, the pivotal issue we are addressing is the transfer of Gene Ontology (GO) terms from the training set to the test set. After acquiring the sets, we proceed to assign functional labels to each protein sequence based on the Gene Ontology terms (GO terms) compiled by Gligorijević et al. These functional labels are categorized into three distinct groups: Molecular Function (MF), Biological Process (BP), and Cellular Components (CC) [45]. Each category serves as an independent prediction task during the training process.

We conduct additional experiment to assess whether recent advancements in protein structure prediction contribute to enhancing domain adaptation. Gligorijević et al. construct the SMch dataset through collecting homology models of the PDBch dataset with

at least one annotation from the SWISS-MODEL repository [46]. Following Gu et al.[27], we select 41,997 proteins from the SMch dataset with low-frequency GO terms (proteins with IC >10 from the PDBch dataset), and retrieve their structures predicted by AlphaFold2 (AF2) from the AlphaFold protein structure database [31]. When selecting a portion from the SWISS-MODEL repository to construct the AFch set, we take into account the highly imbalanced distributions of GO term labels in the PDBch set. For low-frequency GO terms in the PDBch set, we specifically choose proteins with the corresponding GO term labels from the SWISS-Model respository to form the AFch set. This approach helps mitigate the imbalance in PDBch labels to some extent. Detailed information about datasets can be found at **Table 1** and **Supplementary Section 1**.

Table 1. Number of sequences in the datasets.

Datasets	Number of sequences		
	Training set	Validation set	Test set
PDBch set	29304	3660	3665
AFch set	34135	3881	3981

Utilizing the frequency of each GO term in the combined training set (PDBch and AFch), we compute the information content (IC) for each GO term within this set. Higher information content indicates a more specialized GO term.

$$IC(GO_i) = -\log_2(P(GO_i)). \tag{10}$$

Evaluation Metrics

Protein function prediction poses a significant challenge as it involves a highly imbalanced multi-label classification task. Complicating matters, the Gene Ontology (GO) terms associated with these functions are not merely juxtaposed; instead, they exhibit a directed acyclic graph structure. To address the inherent bias and acknowledge the intricate relationships between these terms during model evaluation, we adopt several metrics proposed by the Critical Assessment of Functional Annotation algorithms (CAFA) challenge [13]. These metrics are widely acknowledged and utilized to assess the performance of protein function prediction models.

The first metric is function-centric AUPR, which is calculated by averaging the sum of all $AUPR_f$, where f denotes a certain GO term. AUPR can be formulated as follows:

$$AUPR_f = \sum_t (rc_f(t) - rc_f(t-1)) \times pr_f(t), \tag{11}$$

$$AUPR = \frac{1}{N_f} \sum_f AUPR_f, \tag{12}$$

where t is a cut-off value ranging from 0 to 1 with step size 0.01, rc_f and pr_f represents recall and precision score for the GO term f. Furthermore, N_f is the number of GO terms.

The second metric is protein-centric F_{max}, which is defined as:

$$AvgPr(t) = \frac{1}{m(t)} \sum_{i=1}^{m(t)} pr_i(t), \tag{13}$$

$$AvgRc(t) = \frac{1}{n} \sum_{i=1}^{n} rc_i(t), \tag{14}$$

$$F_{max} = \max_t \left\{ \frac{2 \cdot AvgPr(t) \cdot AvgRc(t)}{AvgPr(t) + AvgRc(t)} \right\}, \tag{15}$$

where $m(t)$ is the number of proteins on which at least one prediction is made above threshold t, $rc_i(t)$ and $pr_i(t)$ represents recall and precision score of protein i at threshold t, and n is the number of proteins.

Given the highly imbalanced nature of the GO term labels, we introduce the Matthews Correlation Coefficient (MCC), computed under a threshold that yields a maximum protein-centric measure F_{max} [8]. MCC value is calculated as follows:

$$MCC = \frac{TP \cdot TN - FP \cdot FN}{\sqrt{(TP+FP)(TP+FN)(TN+FP)(TN+FN)}}, \tag{16}$$

where TP is the number of true positives, FN is the number of false negatives, FP is the number of false positives and TN is the number of true positives.

Additionally, we propose S_{min} to denote the semantic distance between predicted and true annotations, considering information content of GO terms. S_{min} is computed using the following formula:

$$S_{min} = \min_t \sqrt{ru(t)^2 + mi(t)^2}, \tag{17}$$

where $ru(t)$ represents the average uncertainty under threshold t and $mi(t)$ is the average misinformation:

$$ru(t) = \frac{1}{n} \sum_{i=1}^{n} \sum_{c \in T_i - P_i(t)} IC(c|Pa(c)), \tag{18}$$

$$mi(t) = \frac{1}{n} \sum_{i=1}^{n} \sum_{c \in P_i(t) - T_i} IC(c|Pa(c)). \tag{19}$$

Here, T_i and $P_i(t)$ represent the ground truth and predicted labels for protein i, respectively. And $Pa(c)$ represents the parents of term c, and the information content is computed based on the posterior probability of term c, that is, $IC(c|P(c)) = -\log(P(c|Pa(c)))$.

Results

To thoroughly assess the efficiency of our proposed method, GALA, we conduct a comprehensive comparison with several baseline methods, which include a sequence alignment-based method (Blast) and five deep learning-based methods (DeepGOPlus, TALE+, DeepFRI, Struct2GO, and HEAL) under several test sets. To facilitate differentiation, the model obtained using our method trained solely on the PDBch training set is called GALA-PDB, while the model trained on both the PDBch and AFch training sets is called GALA. Given the lower sequence identity between the training and test sets, our objective is to evaluate the performance of these methods when training and test sets are dissimilar. For a fair comparison, all the compared methods are retrained on both the PDBch and AFch training sets. **Table 2** presents the training set and input information for the compared methods. Subsequently, their performance is evaluated on our designated sets.

Table 2. Several baseline methods for protein function prediction.

Methods	Training Set	Input Information
Blast	—	Sequence
DeepGOPlus	PDBch +AFch training set	Sequence
TALE+	PDBch + AFch training set	Sequence
DeepFRI	PDBch + AFch training set	Sequence and Structure
Struct2GO	PDBch + AFch training set	Sequence and Structure
HEAL	PDBch + AFch training set	Sequence and Structure
GALA-PDB	PDBch training set	Sequence and Structure
GALA	PDBch + AFch training set	Sequence and Structure

Table 3. Comparison of our model with several baseline methods on PDBch test set.[α]

Methods	AUPR (↑)			F_{max} (↑)			Smin (↓)			MCC (↑)		
	MF	BP	CC	MF	BP	CC	MF	BP	CC	MF	BP	CC
Blast	0.1260	0.0363	0.0395	0.4304	0.3815	0.2087	2.5372	10.4218	1.3022	0.3025	0.1701	0.1467
DeepGOplus	0.1356	0.0618	0.0844	0.3844	0.3810	0.3034	2.3199	10.1569	1.2450	0.2716	0.1742	0.1999
TALE+	0.1584	0.0701	0.1299	0.3834	0.3784	0.3152	2.3258	10.0628	1.1999	0.2820	0.1885	0.2268
DeepFRI	0.3206	0.1144	0.2225	0.4923	0.4274	0.3249	1.9660	9.7596	1.1112	0.4460	0.2332	0.2688
Struct2GO	0.5234	<u>0.2124</u>	<u>0.3248</u>	0.6417	0.5376	0.3741	1.4829	<u>8.7021</u>	<u>0.9759</u>	0.6453	0.3615	0.3344
HEAL	0.5156	0.1924	0.3176	0.6376	0.5218	<u>0.3830</u>	1.5213	9.1056	0.9892	0.6288	0.3582	**0.3715**
GALA-PDB	<u>0.5386</u>	0.2104	0.3032	<u>0.6710</u>	<u>0.5519</u>	0.3712	**1.4134**	8.8459	1.0193	<u>0.6592</u>	<u>0.3794</u>	<u>0.3676</u>
GALA	**0.5553**	**0.2529**	**0.3625**	**0.6730**	**0.5833**	**0.3854**	<u>1.4212</u>	**8.3964**	**0.9343**	**0.6730**	**0.4253**	0.3621

[α] The entry in bold indicates the best performance, while the underlined entry represents the second-best performance.

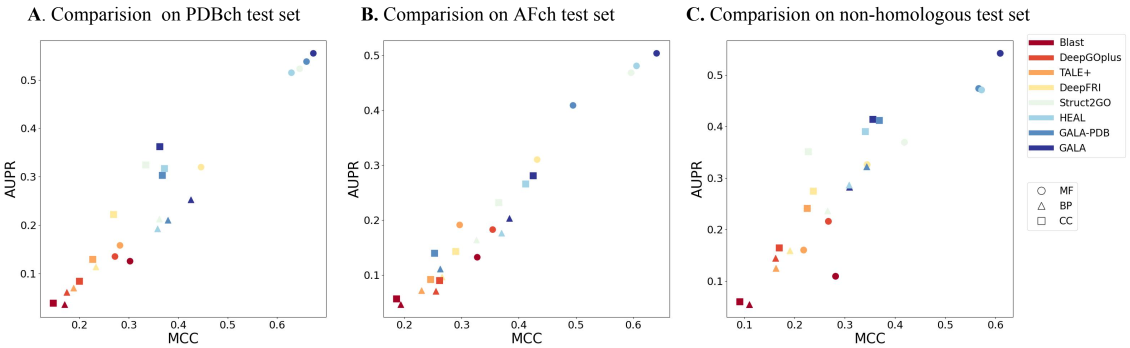

Figure 2. Comparison of GALA with several baseline methods based on two pairwise metrics, AUPR and MCC. In these figures, squares represent testing on MF GO terms, triangles represent testing on BP GO terms, and circles represent testing on CC GO terms. The left subfigure (A) shows the comparison between GALA and baseline methods on the PDBch test set, the subfigure (B) presents the comparison on the AFch test set, and the right subfigure (C) illustrates the comparison on the non-homologous PDBch test set.

Performance of protein function prediction and domain adaptation

Table 3 provides a summary of the overall results for all six protein prediction methods across three gene ontology domains (MF, BP and CC), and we show the best performance in bold and the second performance underline for better comparison. As we train GALA solely with PDBch training test, we name it as GALA-PDB. GALA-PDB achieves AUPR scores of 0.5386, 0.2104 and 0.3032, F_{max} scores of 0.6710, 0.5519 and 0.3712 on MF, BP and CC tasks, respectively. GALA-PDB performs exceptionally well in MF and BP terms, outperforming Blast and other deep learning based methods, including DeepGOPlus, TALE+, DeepFRI, Struct2GO and HEAL, in terms of AUPR,F_{max},S_{min} and MCC. However, it shows slightly lower performance than Struct2GO and HEAL on CC task, while still yielding comparable results. Despite the smaller training set and less available information to the model for GALA-PDB, it performs on par with other methods in areas such as feature extraction, protein function prediction and domain knowledge transfer. In fact, it may even outshine other methods slightly. When incorporating the AFch training set into the training process, the resulting GALA model achieves AUPR scores of 0.5553, 0.2529 and 0.3625, as well as F_{max} scores of 0.6730, 0.5833 and 0.3854 for three gene ontology prediction tasks, respectively. Furthermore, it demonstrates superior performance compared to GALA-PDB and comprehensively outperforms other baseline methods in predicting three different GO tasks, assessed through four distinct evaluation metrics AUPR,F_{max},S_{min} and MCC. As shown in **Figure 2A**, two versions of our method, GALA and GALA-PDB, exhibit superior performance compared to other baseline methods when tested on all GO terms.

Evidently, we observe that our method significantly enhances protein function prediction when compared to several state-of-the-art methods. It demonstrates strong performance even when the sequence identity between the training and test sets is minimal. What's more, it adeptly transfers information from the source domain, composed of the training set, to the target domain, which consists of the test set that is less similar to the source domain. The generalization ability of our method GALA is reflected to some extent.

Performance on GO terms with different specificity

The protein function prediction task is indeed a multi-classification problem, with Molecular Function (MF) comprising 489 terms, Biological Process (BP) comprising 1943 terms, and Cellular Component (CC) comprising 320 terms. It is imperative to address the issue of class imbalance, which can potentially lead to misleading classifications, particularly in deep learning-based methods. In the section of Dataset, we introduce information content metric to assess the specificity of different Gene Ontology (GO) terms, representing the rarity of each GO term. Combining MF, BP and CC terms, we apply a categorization based on information content, utilizing thresholds of 5 and 10. This categorization leads to the division of GO terms into three groups: IC < 5, 5 < IC < 10, and IC > 10. More importantly, this stratification allows for a more nuanced analysis of predictive performance across GO terms with varying degrees of specificity.

As shown in **Figure 3**, the left subfigure illustrates the distribution of information content in the combination of PDBch and AFch training sets. Simultaneously, the right subfigure depicts the performance of different methods across three categories of Gene Ontology (GO) terms, utilizing 10 bootstrap iterations on all test proteins. As the information content value increases, all methods exhibit a consistent downward trend. For commonly occurring terms (IC < 5), GALA and DeepFRI achieve average AUPR scores of 0.4396 and 0.2330, respectively. In the mid-range terms (5 < IC < 10), GALA

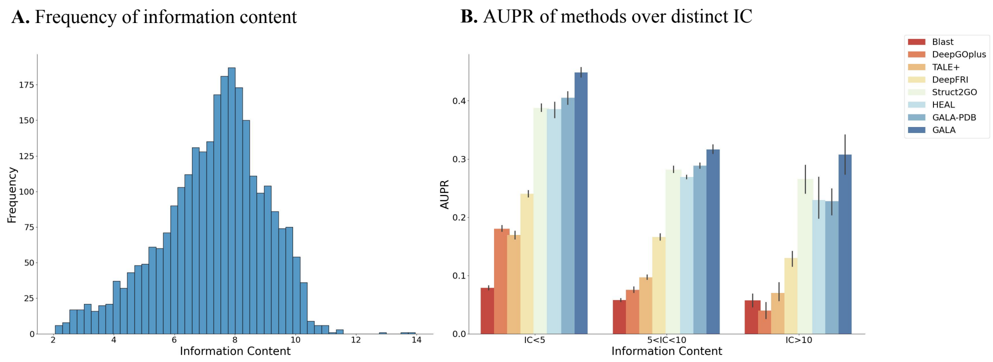

Figure 3. The left subfigure (A) shows the frequency of information content(IC) for protein functions over collection of three categories(MF, BP and CC) in the combination of PDBch training set and AFch training set. The right subfigure (B) shows AUPR of different methods over distinct IC.

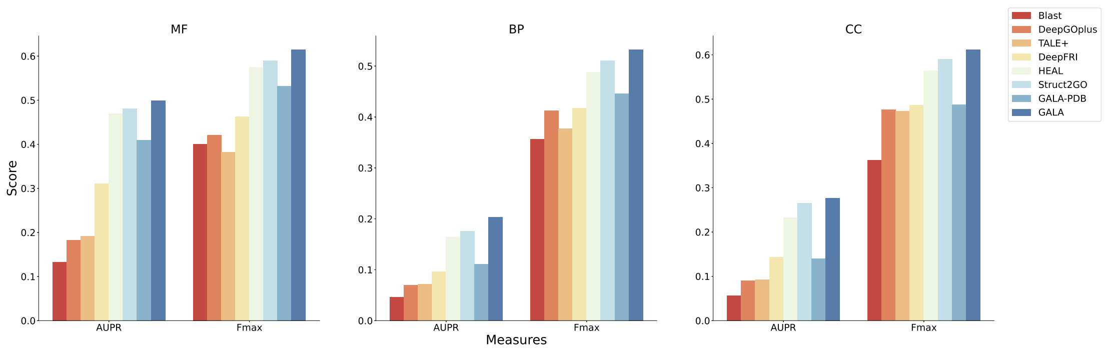

Figure 4. The figure shows AUPR and F_{max} scores of different methods on AFch test set. GALA outperforms all other methods significantly in Molecular Function (MF), Biological Process (BP), and Cellular Component (CC) tasks.

and DeepFRI attain average AUPR scores of 0.3269 and 0.1668, respectively. Meanwhile, on highly specific terms (IC > 10), GALA and DeepFRI achieve average AUPR scores of 0.2885 and 0.1309, respectively. Overall, GALA outperforms all other methods significantly across the three categories of GO terms. Moreover, as GO terms become more specific, GALA exhibits a much slower decrease in performance compared to other methods (**Supplementary Section 2**). Indeed, this observation to a certain extent demonstrates the robustness and superiority of GALA in predicting specific GO terms, aligning with our requirements for accurate and nuanced predictions in the context of protein function prediction.

Performance on AlphaFold2-Predicted structures

For proteins with experimentally resolved structures or highly similar annotated proteins, predicting their functions is relatively straightforward. Therefore, our focus and practical significance lie in proteins with unknown structures that lack homology to existing proteins. Extending functional annotation from well-characterized protein domains to those that are unknown can significantly impact the discovery and understanding of novel proteins in biomedicine and other fields. To assess the model's generalization ability, particularly its performance on proteins with unknown structures, we conduct experiments on the AFch test set whose structures are predicted by AlphaFold2. This set serves as a valuable measure of the model's capability to generalize.

During the dataset preprocessing stage, we selectively remove all protein sequences from the AFch test set with a sequence identity of more than 30% with the combined training set (comprising PDBch and AFch training sets). As illustrated in **Figure 4**, GALA outperforms all other methods significantly in Molecular Function (MF), Biological Process (BP), and Cellular Component (CC) tasks. The AUPR scores for MF, BP and CC are 0.5040, 0.2034 and 0.2813, respectively, while the corresponding F_{max} scores are 0.6140, 0.5340 and 0.6133 (**Supplementary Table S5**). The results are depicted in **Figure 2B, Figure 4**, highlighting the effectiveness of our approach. Notably, GALA-PDB performs poorly due to the exclusion of the AFch set during training, while GALA demonstrates superior performance compared to other methods.

Performance on non-homologous proteins

Given that protein structures diverge much more slowly than sequences, certain proteins with dissimilar sequences can still possess similar structures, indicating distant homology [47]. Furthermore, we utilize both sequence identity and TM-score to identify non-homologous proteins in the PDBch test set. Specifically, proteins in the PDBch test set with a sequence identity of less than 30% and a TM-score [48, 49] below 0.5 from sequences in the PDBch training set are classified as non-homologous. This stricter test set is referred to as the non-homologous PDBch test set. The performance comparison of GALA and several baseline methods are shown in the following table (**Table 4**). Notably, GALA demonstrates exceptional performance on non-homologous PDBch test set. Furthermore, as illustrated in **Figure 2C**, GALA outperforms several baseline methods on non-homologous PDBch test set, based on the pairwise metrics AUPR and MCC.

Performance on proteins newly annotated in the Swiss-Prot database since 2021

Conducting temporal validation is crucial as it provides a comprehensive assessment of our method's robustness over time. Specif-

Table 4. Comparison of our model with several baseline methods on non-homologous PDBch test set.[α]

Methods	AUPR (↑)			F_{max} (↑)			Smin (↓)			MCC (↑)		
	MF	BP	CC	MF	BP	CC	MF	BP	CC	MF	BP	CC
Blast	0.1095	0.0545	0.0601	0.3045	0.2417	0.1955	2.7853	10.5673	1.1936	0.2815	0.1099	0.0909
DeepGOplus	0.2159	0.1445	0.1646	0.3638	0.3014	0.3001	2.6467	10.3173	1.1699	0.2674	0.1620	0.1695
TALE+	0.1602	0.1246	0.2413	0.3100	0.2832	0.3227	2.6626	10.3933	1.1678	0.2175	0.1624	0.2251
DeepFRI	0.3264	0.1588	0.2749	0.3761	0.3188	0.3131	2.4628	9.9593	1.1309	0.3441	0.1908	0.2368
Struct2GO	0.3694	0.2362	0.3511	0.4554	0.3674	0.3690	2.2184	9.5941	**1.0258**	0.4187	0.2656	0.2274
HEAL	0.4709	<u>0.2868</u>	0.3905	0.5024	0.4093	<u>0.3843</u>	**1.9050**	9.4427	1.0462	<u>0.5719</u>	0.3085	0.3410
GALA-PDB	<u>0.4742</u>	**0.3221**	<u>0.4121</u>	**0.5147**	<u>0.4213</u>	0.3734	1.9398	<u>9.1878</u>	<u>1.0344</u>	0.5667	**0.3437**	**0.3685**
GALA	**0.5423**	0.2821	**0.4142**	<u>0.5082</u>	**0.4385**	**0.3951**	<u>1.9259</u>	**9.0331**	1.0599	**0.6092**	<u>0.3091</u>	<u>0.3558</u>

[α] The entry in bold indicates the best performance, while the underlined entry represents the second-best performance.

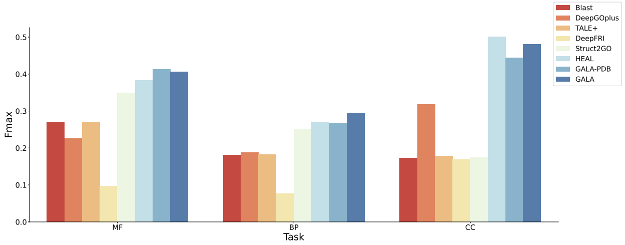

Figure 5. The figure shows F_{max} scores of different methods on the test set, composed of newly annotated proteins in the Swiss-Prot database since 2021.

ically, we collect proteins newly annotated and reviewed in the Swiss-Prot database [4, 50] between January 2021 and June 2024, resulting in a total of 7799 proteins. We then select proteins with sequence identity less than 30% of those in the combined PDBch and AFch training set, forming a newly annotated test set comprising 2270 proteins with predicted structures in AlphaFold database. Consequently, we evaluate the performance of different methods on this newly annotated test set, as shown in the following figure (**Figure 5**). Significantly, GALA exhibits outstanding performance on the newly annotated test set, confirming its robustness over time.

Key residues identification and analysis

To demonstrate the biological interpretability of GALA, we employ Grad-CAM [51] to identify the key residues contributing to the corresponding GO annotation function, effectively discerned by GALA. In our context, we utilize the output of the final graph convolution layer represented as $F \in \mathcal{R}^{L\times D}$, where L denotes the number of protein residues and D is the dimension of the feature space, as the feature map for this purpose. Then we take the derivative of the protein function y_l with respect to F as the gradient weight $W^l_{i,j}$:

$$W^l_{i,j} = \frac{\partial y_l}{\partial F_{i,j}} \tag{20}$$

The contribution score of the ith residue to the lth function CAM^l_i can be obtained as:

$$CAM^l_i = Relu(\frac{\sum_{j=1}^{D} W^l_{i,j} \cdot F_{i,j}}{D}) \tag{21}$$

which is subsequently normalized to fall within the range of 0 to 100 and then we generate heatmaps to illustrate the contribution scores. Furthermore, we project the heatmap onto the protein structure and observe sites with a strong signal, as depicted in **Figure 6**.

For MF-GO terms, we provide two cases where the generated heatmaps align with experimentally confirmed binding sites. In the first example, 3DNF (**Figure 6A**, **Supplementary Figure S2**), a protein associated with the function of iron-sulfur cluster binding (GO:0051536), exhibits strong signals in key residues binding with the iron-sulfur cluster. The second example, 2ZSC (**Figure 6B**, **Supplementary Figure S3**), a protein involved in monocarboxylic acid binding (GO:0033293), reveals regions of strong signal surrounding its binding sites. For BP-GO terms, an example is presented, namely, 1P4U (**Figure 6C**, **Supplementary Figure S4**), with the function of peptide transport (GO:0015833). The residues of 1P4U within the peptide binding interface demonstrate significant Grad-CAM signal.

We proceeded to extract the binding sites of the three proteins from the BioLiP database [52]. What's more, we compare the high-contribution residues identified by Grad-CAM with those experimentally verified in the binding sites. As illustrated in **Figure 6D**, the area under the ROC curve (AUC-ROC) demonstrates that our model possesses an excellent capability to capture functional residues, providing strong evidence for its biological interpretability.

Ablation study

To investigate the effectiveness of various modules in GALA to its enhanced performance, we design an ablation study. In this study, we systematically introduce various modules incrementally to construct advanced models. Specifically, we denote the models as M1 (corresponding to GALA-PDB), M2, M3, M4 and M5 (representing the complete GALA model). This experimental design allows us to analyze and understand the contribution of each module to the overall improvement in performance.

In M1, the PDBch set is solely employed as the training set. The network undergoes training with domain adaptation from the source domain to the target domain, coupled with the utilization of contrastive loss which aligns the protein embedding with the label embedding in the latent space. The key distinction between M1 and M5 lies in whether the AFch set is included in the training process. Moving on to M2, this model is trained with the combined set of PDBch and AFch without domain alignment module and label embedding alignment. Additionally, M3 incorporates transfer loss from source data to target data on the basis of M2. Building upon M3, M5 introduces alignment between protein and label embeddings from labeled source data, which is implemented on the foundation of M3, further refining the model's ability to capture and transfer information across different domains.To demonstrate the effectiveness of domain alignment module, we exclude the adversarial loss from training, as compared to M5.

Table 5 presents AUPR,F_{max},S_{min} values for five models across three GO aspects on the PDBch test set. Upon comparing M1 and M5, the significance of incorporating the AFch set into model training becomes evident, resulting in improved performance across all three GO terms. This observation suggests that the protein structures predicted by AlphaFold2 can enhance the efficiency of protein function prediction. Further comparisons between M2, M3, M4 and M5 reveal a progressive enhancement in the performance of

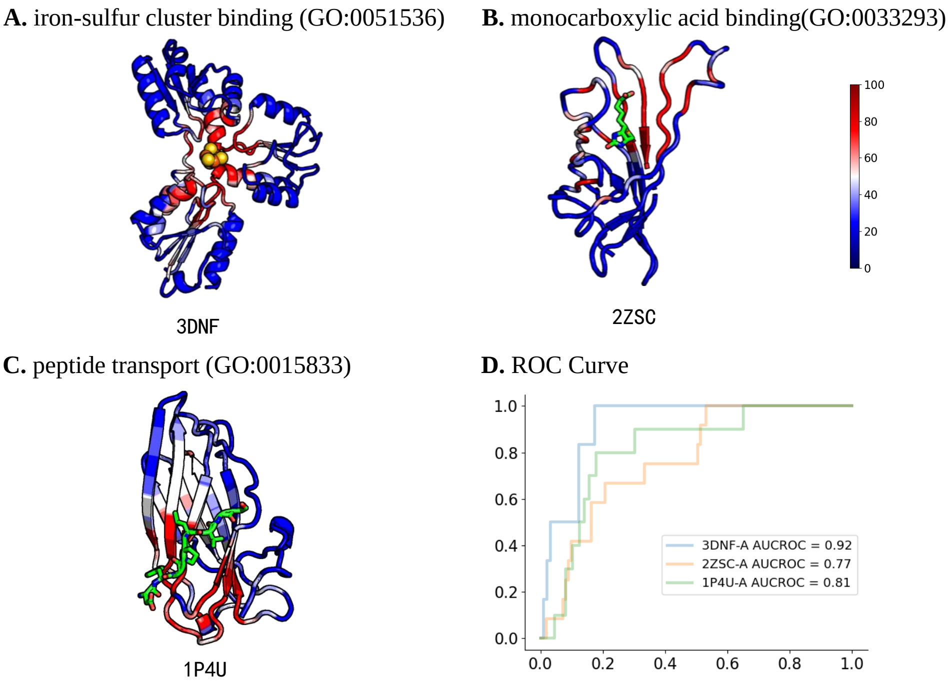

Figure 6. Four examples of the grad-CAM activation profiles mapped onto the experimentally solved structures. (A), (B) and (C) are protein structures colored by the contribution scores computed by Grad-CAM, (D) ROC curves indicate that contribution scores computed by grad-CAM overlap with binding sites retrieved from the BioLiP database.

Table 5. Ablation study of GALA on PDBch test set. $^{\alpha}$

	Modules			AUPR(↑)			F_{max}(↑)			S_{min}(↓)		
	AFch	adv	cl	MF	BP	CC	MF	BP	CC	MF	BP	CC
M1		✓	✓	0.5386	0.2104	0.3032	0.6710	0.5519	0.3712	1.4134	8.8459	1.0193
M2	✓			0.5269	0.1903	0.3160	0.6426	0.5292	0.3925	1.5131	8.9711	0.9875
M3	✓	✓		0.5338	0.2043	0.3207	0.6522	0.5372	0.3911	1.4947	8.9335	0.9866
M4	✓		✓	0.5482	0.2387	0.3234	0.6692	0.5736	0.3800	1.4662	8.4983	0.9682
M5	✓	✓	✓	0.5553	0.2529	0.3625	0.6730	0.5833	0.3854	1.4212	8.3964	0.9343

$^{\alpha}$ AFch, adv, and cl correspond to training with AFch set, adversarial learning for domain alignment, and label embedding alignment.

GALA. Notably, the inclusion of the domain alignment module and the protein-label embedding alignment module contribute to this improvement, which can be proved by experimental results on the Molecular Function (MF) and Biological Process (BP) aspects. While we acknowledge potential conflicts in the Fmax score for predicting CC terms across different methods, we posit that AUPR and Smin metrics for CC terms validate the utility of our diverse modules. Moreover, the three metrics employed for predicting MF and BP terms collectively underscore the comprehensive effectiveness of those modules.

Discussion

In this work, we have proposed GALA for protein prediction and generalization to proteins with dissimilar sequences, leveraging both sequence and structure information as input. For proteins dissimilar with known ones and lacking experimentally analyzed structures, utilizing AlphaFold2 to predict structures and then feeding them into our model aligns well with real-world scenarios. GALA employs adversarial learning and label embedding alignment to learn domain-invariant representations and enhance the model's generalization ability. More importantly, the model outperforms several state-of-the-art methods, showcasing superior generalization capabilities to novel proteins dissimilar to known ones. GALA also demonstrates the close relationship between protein functions and key residues, highlighting the interpretability and generalization ability of our model.

Looking ahead, we plan to incorporate the hierarchical directed acyclic structure of GO terms in order to optimize the training process, while many methods take an extra post-processing step during model evaluation to prevent hierarchy violations. Furthermore, as sequencing technology advances and protein structure-related methods develop, an increasing amount of protein-related information will become accessible. The integration of protein-protein interactions into the model can offer richer information for protein functional annotation, enhancing the overall generalizability of the model.

Additional Files

Supplementary file. (1) Detailed information about the construction of datasets, (2) Description of several baseline methods, (3) AUPR comparison for GO terms on PDBch test set, (4) Performance on PDBch test set under different specificity, (5) Performance on AFch test set, (6) Runtime for several cases and (7) Plots for interpretability of key redisues.

Supplementary Figure S1. Frequency of IC for protein functions over collection of three categories(MF, BP and CC) in the combination of PDBch training set.
Supplementary Figure S2. Contribution score computed by Grad-CAM of protein 3DNF with function of iron-sulfur cluster binding (GO:0051536).
Supplementary Figure S3. Contribution score computed by Grad-CAM of protein 2ZSC with function of monocarboxylic acid binding (GO:0033293).
Supplementary Figure S4. Contribution score computed by Grad-CAM of protein 1P4U with function of peptide transport (GO:0015833).
Supplementary Table S1. AUPR comparison for MF-GO terms on PDBch test set.
Supplementary Table S2. AUPR comparison for BP-GO terms on PDBch test set.
Supplementary Table S3. AUPR comparison for CC-GO terms on PDBch test set.
Supplementary Table S4. Performance of GALA and other baseline methods on PDBch test set under different specificity.
Supplementary Table S5. Performance of GALA and other baseline methods on AFch test set.
Supplementary Table S6. The running times for several cases.

Data Availability

Supporting datasets for this article are sourced from DeepFRI [10].The first dataset, named PDBch, is selected from the PDB database and clustered using MMseqs [53] at a sequence identity of 30%. The training, validation, and test sets are chosen from different clusters with an approximate ratio of 8:1:1. As for the second dataset, AFch, we initially select 41,997 proteins from SWISS-MODEL and then partition them into training, validation, and test sets similar to the PDBch dataset. For more detailed information, refer to Section Dataset. An archival copy of the code and other data further supporting this work is openly available in the GigaScience repository, GigaDB[54]. Furthermore, a link to DOME-ML (Data, Optimization, Model and Evaluation in Machine Learning) annotations is available via GigaDB[54].

Availability of supporting source code and requirements

- Project name: GALA
- Project home page: `https://github.com/fuyw-aisw/GALA`
- Operating system(s): Platform independent
- Programming language: Python
- Other requirements: not applicable
- License: MIT license
- RRID: SCR_025194
- Docker package: fuyw99/gala

Competing interests

No competing interest is declared.

Funding

This work was supported by the National Key Research and Development Program of China (2021YFF1200902) and the National Natural Science Foundation of China (32270689).

References

1. Eisenberg D, Marcotte EM, Xenarios I, Yeates TO. Protein function in the post-genomic era. Nature 2000;405(6788):823–826.
2. Berman HM, Westbrook J, Feng Z, Gilliland G, Bhat TN, Weissig H, et al. The protein data bank. Nucleic Acids Research 2000;28(1):235–242.
3. Apweiler R, Bairoch A, Wu CH, Barker WC, Boeckmann B, Ferro S, et al. UniProt: the universal protein knowledgebase. Nucleic Acids Research 2004;32:D115–D119.
4. Boutet E, Lieberherr D, Tognolli M, Schneider M, Bairoch A. UniProtKB/Swiss-Prot: the manually annotated section of the UniProt KnowledgeBase. In: Plant Bioinformatics: Methods and Protocols Springer; 2007.p. 89–112.
5. Consortium U. UniProt: a worldwide hub of protein knowledge. Nucleic Acids Research 2019;47(D1):D506–D515.
6. Zhou N, Jiang Y, Bergquist TR, Lee AJ, Kacsoh BZ, Crocker AW, et al. The CAFA challenge reports improved protein function prediction and new functional annotations for hundreds of genes through experimental screens. Genome Biology 2019;20(1):1–23.
7. You R, Zhang Z, Xiong Y, Sun F, Mamitsuka H, Zhu S. GOLabeler: improving sequence-based large-scale protein function prediction by learning to rank. Bioinformatics 2018;34(14):2465–2473.
8. Kulmanov M, Khan MA, Hoehndorf R. DeepGO: predicting protein functions from sequence and interactions using a deep ontology-aware classifier. Bioinformatics 2018;34(4):660–668.
9. You R, Yao S, Xiong Y, Huang X, Sun F, Mamitsuka H, et al. NetGO: improving large-scale protein function prediction with massive network information. Nucleic acids research 2019;47(W1):W379–W387.
10. Gligorijević V, Renfrew PD, Kosciolek T, Leman JK, Berenberg D, Vatanen T, et al. Structure-based protein function prediction using graph convolutional networks. Nature Communications 2021;12(1):3168.
11. Altschul SF, Gish W, Miller W, Myers EW, Lipman DJ. Basic local alignment search tool. Journal of Molecular Biology 1990;215(3):403–410.
12. Das S, Lee D, Sillitoe I, Dawson NL, Lees JG, Orengo CA. Functional classification of CATH superfamilies: a domain-based approach for protein function annotation. Bioinformatics 2015;31(21):3460–3467.
13. Radivojac P, Clark WT, Oron TR, Schnoes AM, Wittkop T, Sokolov A, et al. A large-scale evaluation of computational protein function prediction. Nature Methods 2013;10(3):221–227.
14. Fa R, Cozzetto D, Wan C, Jones DT. Predicting human protein function with multi-task deep neural networks. PloS One 2018;13(6):e0198216.
15. Zhang X, Wang L, Liu H, Zhang X, Liu B, Wang Y, et al. Prot2GO: predicting GO annotations from protein sequences and interactions. IEEE/ACM Transactions on Computational Biology and Bioinformatics 2021;p. 1–1.
16. Cao Y, Shen Y. TALE: Transformer-based protein function Annotation with joint sequence–Label Embedding. Bioinformatics 2021;37(18):2825–2833.
17. Kulmanov M, Hoehndorf R. DeepGOPlus: improved protein function prediction from sequence. Bioinformatics 2021;37(8):1187.
18. Sharan R, Ulitsky I, Shamir R. Network-based prediction of protein function. Molecular Systems Biology 2007;3(1):88.
19. Mostafavi S, Ray D, Warde-Farley D, Grouios C, Morris Q. GeneMANIA: a real-time multiple association network integration algorithm for predicting gene function. Genome Biology 2008;9(1):1–15.

20. Jiang JQ, McQuay LJ. Predicting protein function by multi-label correlated semi-supervised learning. IEEE/ACM Transactions on Computational Biology and Bioinformatics 2011;9(4):1059–1069.
21. Cho H, Berger B, Peng J. Compact integration of multi-network topology for functional analysis of genes. Cell Systems 2016;3(6):540–548.
22. You Y, Chen T, Shen Y, Wang Z. Graph Contrastive Learning Automated. In: Proceedings of the International Conference on Machine Learning; 2021. p. 12121–12132.
23. Gaudet P, Livstone MS, Lewis SE, Thomas PD. Phylogenetic-based propagation of functional annotations within the Gene Ontology consortium. Briefings in Bioinformatics 2011;12(5):449–462.
24. Konc J, Hodošček M, Ogrizek M, Trykowska Konc J, Janežič D. Structure-based function prediction of uncharacterized protein using binding sites comparison. PLoS Computational Biology 2013;9(11):e1003341.
25. Lai B, Xu J. Accurate protein function prediction via graph attention networks with predicted structure information. Briefings in Bioinformatics 2022;23(1):bbab502.
26. Ma W, Zhang S, Li Z, Jiang M, Wang S, Lu W, et al. Enhancing protein function prediction performance by utilizing AlphaFold-predicted protein structures. Journal of Chemical Information and Modeling 2022;62(17):4008–4017.
27. Gu Z, Luo X, Chen J, Deng M, Lai L. Hierarchical graph transformer with contrastive learning for protein function prediction. Bioinformatics 2023;39(7):btad410.
28. Verspoor KM. Roles for text mining in protein function prediction. Biomedical Literature Mining 2014;p. 95–108.
29. Yao S, You R, Wang S, Xiong Y, Huang X, Zhu S. NetGO 2.0: improving large-scale protein function prediction with massive sequence, text, domain, family and network information. Nucleic acids research 2021;49(W1):W469–W475.
30. Zhou X, Zheng W, Li Y, Pearce R, Zhang C, Bell EW, et al. I-TASSER-MTD: a deep-learning-based platform for multi-domain protein structure and function prediction. Nature Protocols 2022;17(10):2326–2353.
31. Varadi M, Anyango S, Deshpande M, Nair S, Natassia C, Yordanova G, et al. AlphaFold Protein Structure Database: massively expanding the structural coverage of protein-sequence space with high-accuracy models. Nucleic Acids Research 2022;50(D1):D439–D444.
32. Baek M, DiMaio F, Anishchenko I, Dauparas J, Ovchinnikov S, Lee GR, et al. Accurate prediction of protein structures and interactions using a three-track neural network. Science 2021;373(6557):871–876.
33. Lin Z, Akin H, Rao R, Hie B, Zhu Z, Lu W, et al. Evolutionary-scale prediction of atomic-level protein structure with a language model. Science 2023;379(6637):1123–1130.
34. Wang S, You R, Liu Y, Xiong Y, Zhu S. NetGO 3.0: protein language model improves large-scale functional annotations. Genomics, Proteomics & Bioinformatics 2023;21(2):349–358.
35. Zhang C, Zheng W, Freddolino PL, Zhang Y. MetaGO: Predicting Gene Ontology of non-homologous proteins through low-resolution protein structure prediction and protein–protein network mapping. Journal of molecular biology 2018;430(15):2256–2265.
36. Rives A, Meier J, Sercu T, Goyal S, Lin Z, Liu J, et al. Biological structure and function emerge from scaling unsupervised learning to 250 million protein sequences. Proceedings of the National Academy of Sciences 2021;118(15):e2016239118.
37. Xu K, Hu W, Leskovec J, Jegelka S. How powerful are graph neural networks? In: Proceedings of the International Conference on Learning Representations; 2019. .
38. Gilmer J, Schoenholz SS, Riley PF, Vinyals O, Dahl GE. Message passing neural networks. In: Machine learning meets quantum physics Springer; 2020.p. 199–214.
39. Baek J, Kang M, Hwang SJ. Accurate Learning of Graph Representations with Graph Multiset Pooling. In: International Conference on Learning Representations; 2021. https://openreview.net/forum?id=JHcqXGaqiGn.
40. Vaswani A, Shazeer N, Parmar N, Uszkoreit J, Jones L, Gomez AN, et al. Attention is all you need. In: Proceedings of the Conference on Neural Information Processing Systems; 2017. .
41. Long M, Cao Z, Wang J, Jordan MI. Conditional adversarial domain adaptation. Advances in Neural Information Processing Systems 2018;31.
42. Kingma DP, Ba J. Adam: A method for stochastic optimization. arXiv preprint arXiv:14126980 2014;.
43. Dana JM, Gutmanas A, Tyagi N, Qi G, O'Donovan C, Martin M, et al. SIFTS: updated Structure Integration with Function, Taxonomy and Sequences resource allows 40-fold increase in coverage of structure-based annotations for proteins. Nucleic Acids Research 2019;47(D1):D482–D489.
44. Mirdita M, Steinegger M, Breitwieser F, Söding J, Levy Karin E. Fast and sensitive taxonomic assignment to metagenomic contigs. Bioinformatics 2021;37(18):3029–3031.
45. Ashburner M, Ball CA, Blake JA, Botstein D, Butler H, Cherry JM, et al. Gene ontology: tool for the unification of biology. Nature Genetics 2000;25(1):25–29.
46. Waterhouse A, Bertoni M, Bienert S, Studer G, Tauriello G, Gumienny R, et al. SWISS-MODEL: homology modelling of protein structures and complexes. Nucleic Acids Research 2018;46(W1):W296–W303.
47. Liu W, Wang Z, You R, Xie C, Wei H, Xiong Y, et al. PLM-Search: Protein language model powers accurate and fast sequence search for remote homology. Nature communications 2024;15(1):2775.
48. Zhang Y, Skolnick J. TM-align: a protein structure alignment algorithm based on the TM-score. Nucleic acids research 2005;33(7):2302–2309.
49. van Kempen M, Kim SS, Tumescheit C, Mirdita M, Gilchrist CL, Söding J, et al. Foldseek: fast and accurate protein structure search. Biorxiv 2022;p. 2022–02.
50. Boutet E, Lieberherr D, Tognolli M, Schneider M, Bansal P, Bridge AJ, et al. UniProtKB/Swiss-Prot, the manually annotated section of the UniProt KnowledgeBase: how to use the entry view. Plant bioinformatics: methods and protocols 2016;p. 23–54.
51. Selvaraju RR, Cogswell M, Das A, Vedantam R, Parikh D, Batra D. Grad-CAM: Visual Explanations from Deep Networks via Gradient-Based Localization. International Journal of Computer Vision 2020;128(2):336–359.
52. Yang J, Roy A, Zhang Y. BioLiP: a semi-manually curated database for biologically relevant ligand–protein interactions. Nucleic Acids Research 2012 10;41(D1):D1096–D1103.
53. Steinegger M, Söding J. Clustering huge protein sequence sets in linear time. Nature communications 2018;9(1):2542.
54. Fu Y, Gu Z, Luo X, Guo Q, Lai l, Deng M, Supporting data for "Learning A Generalized Graph Transformer for Protein Function Prediction in Dissimilar Sequences". GigaScience Database; 2024. https://doi.org/10.5524/102588.

Click here to access/download
Supplementary Material
GALA_SI_revision_3.pdf

Click here to access/download;Personal Cover;CoverLetter_DA.pdf

Peking University
Beijing 100871
China

Dear editors,

We hereby submit a manuscript entitled "**GALA: A Generalized Graph-based Method for Protein Function Prediction in Dissimilar Sequences**" to be considered for publication as a *Research Paper* in *GigaScience.*

Modern data-driven approaches are capable of facilitating fast and accurate predictions of protein functions. However, the inherent statistical nature of deep learning techniques may limit their generalization capabilities when applied to new protein sequences dissimilar to existing ones. This paper presents a new approach named HEAL for this problem, which improves the quality of graph assignments from the perspectives of class balancing and uncertainty mining. Extensive experiments on a variety of benchmarks demonstrate the effectiveness of our approach over competitive baselines with high generalizability on AUPR and Fmax. Moreover, our GALA holds excellent interpretability in finding key functional residues. We believe that our work presents significant technical advances, and will be critical to the fields of protein understanding and computational biology. As such, it should attract the broad readership of *GigaScience.*

The final manuscript has been seen and approved by all authors, and we declare no competing financial interests. The source code to implement our approach is publicly available at https://github.com/fuyw-aisw/GALA.

Thanks again for your consideration, and we look forward to your comments.

With Best Regards,
Dr. Minghua Deng
