# Supplementary material for: Learning a generalized graph transformer for protein function prediction in dissimilar sequences
Source: Gigascience. 2024 Dec 5;13:giae093. doi: 10.1093/gigascience/giae093 (PMC11734293; doi:10.1093/gigascience/giae093)
Supplement: giae093_Response_to_Reviewer_Comments_Original_Submission [file giae093_response_to_reviewer_comments_original_submission.pdf]

Author's Response To Reviewer Comments

Hans Zauner, Ph.D.
GigaScience
Dear Editor:

Thanks for forwarding the valuable comments on our manuscript “Learning A Generalized Graph Transformer for Protein Function Prediction in Dissimilar Sequences” (GIGA-D-24-00109). We have made extensive revisions based on these valuable comments, which we briefly summarize below for your convenience, and have accordingly revised our article with minor changes highlighted in red.

Summary of Major Changes
1.We incorporated additional baseline methods to more thoroughly evaluate GALA's performance.

2.We imposed structural similarity constraints to minimize homology between the training set and the test set, thereby providing a more rigorous assessment of GALA's generalization capability.

3.We selected reviewed and newly annotated proteins from the UniProtKB/Swiss-Prot database that are dissimilar to the training set to evaluate GALA's potential for real-world applications.

We believe our work presents significant technical advances in protein analysis, and hope the paper is now acceptable for publication in GigaScience. Once again, we greatly appreciate your effort and time spent revising our manuscript.

With Best Regards,
Dr. Minghua Deng
Peking University
