# Supplementary material for: Learning a generalized graph transformer for protein function prediction in dissimilar sequences
Source: Gigascience. 2024 Dec 5;13:giae093. doi: 10.1093/gigascience/giae093 (PMC11734293; doi:10.1093/gigascience/giae093)
Supplement: giae093_Response_to_Reviewer_Comments_Revision_1 [file giae093_response_to_reviewer_comments_revision_1.pdf]

# Author's Response To Reviewer Comments

Hans Zauner, Ph.D.
GigaScience
Dear Editor:

Thanks for forwarding the valuable comments on our manuscript “Learning A Generalized Graph Transformer for Protein Function Prediction in Dissimilar Sequences” (GIGA-D-24-00109_R1). We have made extensive revisions based on these valuable comments, which we briefly summarize below for your convenience, and have accordingly revised our article with minor changes highlighted in red. And we have attached a letter named “GALA_reviewer_response_2” with a detailed point-by-point response.

Summary of Major Changes
We included the results of all seven baseline methods in the tables and figures to provide a more comprehensive evaluation.
We added a comparison based on the two metrics, AUPR and MCC, to convincingly demonstrate GALA’s performance across all testing GO terms.

We believe our work presents significant technical advances in protein analysis, and hope the paper is now acceptable for publication in GigaScience. Once again, we greatly appreciate your effort and time spent revising our manuscript.

With Best Regards,
Dr. Minghua Deng
Peking University
