# Supplementary material for: Learning a generalized graph transformer for protein function prediction in dissimilar sequences
Source: Gigascience. 2024 Dec 5;13:giae093. doi: 10.1093/gigascience/giae093 (PMC11734293; doi:10.1093/gigascience/giae093)
Supplement: giae093_Response_to_Reviewer_Comments_Revision_2 [file giae093_response_to_reviewer_comments_revision_2.pdf]

Author's Response To Reviewer Comments

Hans Zauner, Ph.D.
GigaScience
Dear Editor:

Thanks for forwarding the valuable comments on our manuscript "Learning A Generalized Graph Transformer for Protein Function Prediction in Dissimilar Sequences" (GIGA-D-24-00109_R3). We have revised our manuscript accordingly.

1. The GigaDB dataset has been cited in the "Data Availability" section.

2. DOME-ML annotations have been mentioned accordingly.

3. The URLs for data sources have been correctly revised in the text.

4. ORCIDs have been added to the title page.

5. All tracked changes marked in red have been removed from the manuscript.

6.The LaTeX source files have been attached.

Additionally, the RRID from SciCrunch.org databases has been included in the "Availability of Supporting Source Code and Requirements" section, with the RRID being "SCR_025194".

We believe our work presents significant technical advances in protein analysis, and hope the paper is now acceptable for publication in GigaScience. Once again, we greatly appreciate your effort and time spent revising our manuscript.

With Best Regards,
Dr. Minghua Deng
Peking University
