# Supplementary material for: Learning a generalized graph transformer for protein function prediction in dissimilar sequences
Source: Gigascience. 2024 Dec 5;13:giae093. doi: 10.1093/gigascience/giae093 (PMC11734293; doi:10.1093/gigascience/giae093)
Supplement: giae093_Reviewer_1_Report_Original_Submission — Xiaogen Zhou -- 5/7/2024 [file giae093_reviewer_1_report_original_submission.pdf]

Reviewer Report

Title: Learning A Generalized Graph Transformer for Protein Function Prediction in Dissimilar Sequences

Version: Original Submission **Date:** 5/7/2024

Reviewer name: Xiaogen Zhou

Reviewer Comments to Author:

This manuscript introduces a protein function prediction method called GALA. This method leverages adversarial learning and label embedding alignment to ensure representation consistency across diverse environments and dissimilar protein sequences. The authors also compare this method with several state-of-the-art methods, and results indicate the superiority over alternative approaches. However, to further enhance the clarity and depth of the manuscript, I would suggest seeking clarification from the authors regarding a few specific queries before recommending it for publication.1. Methods for function prediction through partner-homology based protein-protein interaction mapping should be reviewed in Introduction. For example, MetaGO and the I-TASSER-MTD employed pipeline.2. It seems that ESM-1b can only accept protein sequences shorter than 1024. How did the authors handle longer sequences?3. In Equation 9, the authors directly sum multiple losses. Would this approach potentially lead to one particular loss dominating during training?4. The difference between GALA-PDB and GALA needs further clarification.5. Authors have compared their method with TALE+, DeepFRI, Struct2GO, and HEAL, I strongly suggest them compare the method with NetGO 3.0, which is a top ranked method in CAFA5.6. Although the required time varies depending on the structure, providing an estimate or an example of the runtime for a specific case would be valuable.7. Since AlphaFold2 trained on all proteins from PDB, it is difficult to remove the target protein itself or homologous proteins when modeling the 3D structure. How does the author handle this situation need further discussion.

Level of Interest

Please indicate how interesting you found the manuscript: Choose an item.

Quality of Written English

Please indicate the quality of language in the manuscript: Choose an item.

Declaration of Competing Interests

Please complete a declaration of competing interests, considering the following questions:

- Have you in the past five years received reimbursements, fees, funding, or salary from an organisation that may in any way gain or lose financially from the publication of this manuscript, either now or in the future?

- Do you hold any stocks or shares in an organisation that may in any way gain or lose financially from the publication of this manuscript, either now or in the future?
- Do you hold or are you currently applying for any patents relating to the content of the manuscript?
- Have you received reimbursements, fees, funding, or salary from an organization that holds or has applied for patents relating to the content of the manuscript?
- Do you have any other financial competing interests?
- Do you have any non-financial competing interests in relation to this paper?

If you can answer no to all of the above, write 'I declare that I have no competing interests' below. If your reply is yes to any, please give details below.

I declare that I have no competing interests.

I agree to the open peer review policy of the journal. I understand that my name will be included on my report to the authors and, if the manuscript is accepted for publication, my named report including any attachments I upload will be posted on the website along with the authors' responses. I agree for my report to be made available under an Open Access Creative Commons CC-BY license (http://creativecommons.org/licenses/by/4.0/). I understand that any comments which I do not wish to be included in my named report can be included as confidential comments to the editors, which will not be published.

Choose an item.

To further support our reviewers, we have joined with Publons, where you can gain additional credit to further highlight your hard work (see: https://publons.com/journal/530/gigascience). On publication of this paper, your review will be automatically added to Publons, you can then choose whether or not to claim your Publons credit. I understand this statement.

Yes Choose an item.
