# Supplementary material for: Learning a generalized graph transformer for protein function prediction in dissimilar sequences
Source: Gigascience. 2024 Dec 5;13:giae093. doi: 10.1093/gigascience/giae093 (PMC11734293; doi:10.1093/gigascience/giae093)
Supplement: giae093_Reviewer_2_Report_Original_Submission — Amarda Shehu -- 5/11/2024 [file giae093_reviewer_2_report_original_submission.pdf]

Reviewer Report

Title: Learning A Generalized Graph Transformer for Protein Function Prediction in Dissimilar Sequences

Version: Original Submission **Date:** 5/11/2024

Reviewer name: Amarda Shehu

Reviewer Comments to Author:

Overview:The authors noticed that the generic deep learning mechanisms, such as a simple fully connected classifier, may hinder models' generalizability when annotating proteins with a set of functions due to the inherent domain divergence in protein universe. Therefore, the authors adopt three components, with some additional benefits, towards solving protein function prediction tasks, 1) a graph multiset transformer pooling mechanism to learn protein (graph) level representation 2) a multilinear conditional domain adaptation mechanism to better align source and target protein domains, and 3) a label embedding module to learn label representation in the same space. Overall, the authors proposed a novel architecture, namely Graph Adversarial Learning with Alignment (GALA), for protein function prediction by integrating these components.Strengths:. Overall, the model architecture is a novel contribution to the best of my knowledge whereas the different components (such as conditional domain adaptation mechanism, and graph multiset transformer pooling) have been proposed before. However, this adaptation, with proper citation, will progress the protein function prediction problem domain.. This research steps towards biological domain adaptation issue, particularly in protein domain.. By utilizing the structures predicted by AlphaFold2, GALA holds significant potential for function annotation in newly discovered sequences.. The analysis, particularly the ablation study and the key residue identification, shows promise of the proposed model architecture towards protein function prediction. However, Table 3 evident a point that only adversarial component does not contribute significantly.. The sequence clustering-based train/validation/test split is applied to serve the realistic biological condition to show the model's actual ability to learn effective features to solve the downstream prediction tasks.Major revisions:. GALA is a protein function predictor, based on protein sequence and structural input information. It is always notable that only sequence-based models have an upperhand over sequence-plus-structure based models. Therefore, it is highly recommended that Table-2 should summarize which models are sequence based and which are sequence -plus- structure based.. Please consider reporting Smin metrics?. The performance of HEAL in the original paper is different than what the authors reported in this current work, and the discrepancies are huge, for instance Fmax-CCs are 0.687 (HEAL) vs 0.3830 (GALA). What are the differences between HEAL dataset vs GALA dataset generation procedure? What are the number of BP, CC and MF labels utilized after the preprocessing steps?. Why did the authors split the datasets on their own? When training the existing models, how are the hyperparameters optimized?. The training mechanism is not clear, the proposed architecture is not trained end-to-end (I assume), as such please describe how the independent modules are trained/validated in detail.Minor: Source classifier is denoted by "G" in the text whereas in fig1 it is denoted by "H". Eq-7 has two extra first brackets (")").

Level of Interest

Please indicate how interesting you found the manuscript: Choose an item.

Quality of Written English

Please indicate the quality of language in the manuscript: Choose an item.

Declaration of Competing Interests

Please complete a declaration of competing interests, considering the following questions:

- Have you in the past five years received reimbursements, fees, funding, or salary from an organisation that may in any way gain or lose financially from the publication of this manuscript, either now or in the future?
- Do you hold any stocks or shares in an organisation that may in any way gain or lose financially from the publication of this manuscript, either now or in the future?
- Do you hold or are you currently applying for any patents relating to the content of the manuscript?
- Have you received reimbursements, fees, funding, or salary from an organization that holds or has applied for patents relating to the content of the manuscript?
- Do you have any other financial competing interests?
- Do you have any non-financial competing interests in relation to this paper?

If you can answer no to all of the above, write 'I declare that I have no competing interests' below. If your reply is yes to any, please give details below.

I declare that I have no competing interests.

I agree to the open peer review policy of the journal. I understand that my name will be included on my report to the authors and, if the manuscript is accepted for publication, my named report including any attachments I upload will be posted on the website along with the authors' responses. I agree for my report to be made available under an Open Access Creative Commons CC-BY license (http://creativecommons.org/licenses/by/4.0/). I understand that any comments which I do not wish to be included in my named report can be included as confidential comments to the editors, which will not be published.

Choose an item.

To further support our reviewers, we have joined with Publons, where you can gain additional credit to further highlight your hard work (see: https://publons.com/journal/530/gigascience). On publication of

this paper, your review will be automatically added to Publons, you can then choose whether or not to claim your Publons credit. I understand this statement.

Yes Choose an item.
