# Supplementary material for: Learning a generalized graph transformer for protein function prediction in dissimilar sequences
Source: Gigascience. 2024 Dec 5;13:giae093. doi: 10.1093/gigascience/giae093 (PMC11734293; doi:10.1093/gigascience/giae093)
Supplement: giae093_Reviewer_3_Report_Original_Submission — Cen Wan, Ph.D. -- 5/13/2024 [file giae093_reviewer_3_report_original_submission.pdf]

Reviewer Report

Title: Learning A Generalized Graph Transformer for Protein Function Prediction in Dissimilar Sequences

Version: Original Submission **Date:** 5/13/2024

Reviewer name: Cen Wan, Ph.D.

Reviewer Comments to Author:

This paper proposed a novel graph transformer-based protein function prediction method exploiting both protein sequence and structural information. Although the computational experiments show encouraging results, there still exist some concerns as below.Major concerns:1. In terms of the proposed method, it is not clear whether the so-called domain-invariant representations would bring extra discriminative power for predicting different domains of GO terms. I suspect almost all the predictive information could have already been learned by the Graph transformer. As shown in Table 3, the Fmax score obtained by M2 is higher than M3 and M4 for predicting CC terms. Although the authors revealed more information in the ablation study section, there weren't any pairwise comparisons between different modules. For example, the final loss function consists of three terms (i.e. L_sup, L_adv and L_con) - the authors need to justify the usefulness of those three individual terms.2. In terms of the ablation study, as shown in Table 3, the Fmax score obtained by M1 is almost the same as the one obtained by M4 for predicting MF terms. It suggests exploiting the AlphaFold2-predicted structures merely slightly improved the protein-centric predictive accuracy. However, according to the GO term-centric AUPRC values, the AlphaFold2-predicted structures do lead to a greater increase in accuracy. The authors need to further investigate this pattern.3. The authors need to compare GALA with other well-known protein function prediction methods which also exploit both sequence and structural information like MetaGO [1]. It is also necessary to compare GALA with other SOTA methods, e.g. DeepGOPlus [2] and NetGO 3.0 [3].[1] Zhang et al. (2018) MetaGO: Predicting Gene Ontology of non-homologous proteins through low-resolution protein structure prediction and protein-protein network mapping. Journal of Molecular Biology, 430:2256-2265.[2] Kulmanov, M. and Hoehndorf, R. (2020) DeepGOPlus: improved protein function prediction from sequence, Bioinformatics, 36(2):422-429.[3] Wang et al. (2023) NetGO 3.0: Protein Language Model Improves Large-scale Functional Annotations, Genomics Proteomics Bioinformatics, 21(2):349-358.4. Using 30% sequence similarity could be considered a standard approach to avoid data leaking problems between training and testing protein sequence datasets. However, it is not rigorous to claim the outstanding performance of GALA when handling previously unseen sequences. In fact, more importantly, the authors need to evaluate the performance of GALA in predicting non-homologous proteins' functions.5. The experimental datasets were acquired in 2021. The authors need to demonstrate the contribution of GALA by conducting the so-called temporal validation, i.e. using year 2021's database to predict the newly annotated protein functions in the UniProt database up to the present.Minor concerns:6. Since AUPRC is used as a GO term-centric metric, it is necessary to reveal more information regarding each GO term's predictive accuracy, rather than merely showing the summarised numbers in Table 2.7. The authors also need to use more metrics

to evaluate the performance of GALA, e.g. MCC value, due to the highly imbalanced distributions of GO term labels.8. In terms of the biological interpretations, the authors discussed three cases, but no further information was provided regarding selection criteria. Also, one of the cases GO:0043177 is a generic term (merely has three ancestor terms) - it will be better to discuss other terms bearing more specific MF definisions.

Level of Interest

Please indicate how interesting you found the manuscript: Choose an item.

Quality of Written English

Please indicate the quality of language in the manuscript: Choose an item.

Declaration of Competing Interests

Please complete a declaration of competing interests, considering the following questions:

- Have you in the past five years received reimbursements, fees, funding, or salary from an organisation that may in any way gain or lose financially from the publication of this manuscript, either now or in the future?
- Do you hold any stocks or shares in an organisation that may in any way gain or lose financially from the publication of this manuscript, either now or in the future?
- Do you hold or are you currently applying for any patents relating to the content of the manuscript?
- Have you received reimbursements, fees, funding, or salary from an organization that holds or has applied for patents relating to the content of the manuscript?
- Do you have any other financial competing interests?
- Do you have any non-financial competing interests in relation to this paper?

If you can answer no to all of the above, write 'I declare that I have no competing interests' below. If your reply is yes to any, please give details below.

I declare that I have no competing interests

I agree to the open peer review policy of the journal. I understand that my name will be included on my report to the authors and, if the manuscript is accepted for publication, my named report including any attachments I upload will be posted on the website along with the authors' responses. I agree for my report to be made available under an Open Access Creative Commons CC-BY license (http://creativecommons.org/licenses/by/4.0/). I understand that any comments which I do not wish to be included in my named report can be included as confidential comments to the editors, which will not be published.

Choose an item.

To further support our reviewers, we have joined with Publons, where you can gain additional credit to further highlight your hard work (see: https://publons.com/journal/530/gigascience). On publication of this paper, your review will be automatically added to Publons, you can then choose whether or not to claim your Publons credit. I understand this statement.

Yes Choose an item.
